# Supplementary material for: A machine learning approach for online automated optimization of super-resolution optical microscopy
Source: Nat Commun. 2018 Dec 7;9:5247. doi: 10.1038/s41467-018-07668-y (PMC6286316; doi:10.1038/s41467-018-07668-y)
Supplement: Supplementary file 1 — Supplementary Information [file 41467_2018_7668_MOESM1_ESM.pdf]

Supplementary Material

A machine learning approach for automated online optimization of super-resolution optical microscopy

Durand A. et al. 2018

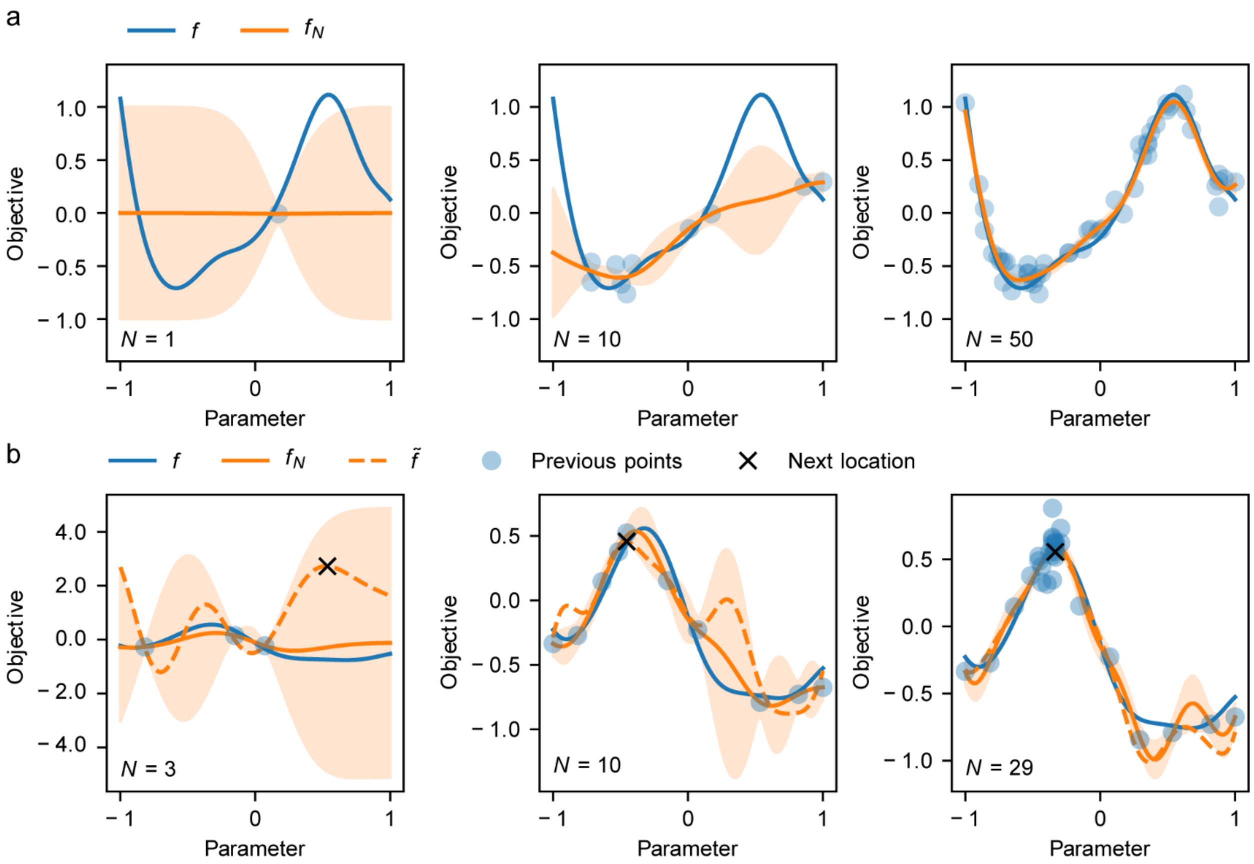

Supplementary Figure 1: Kernel TS for single objective optimization a) Examples of posterior mean and standard deviation conditioned on N previous observations. b) Examples of functions sampled from posterior distributions conditioned on N previous observations. The point maximizing the sampled function would be the next parameter selected.

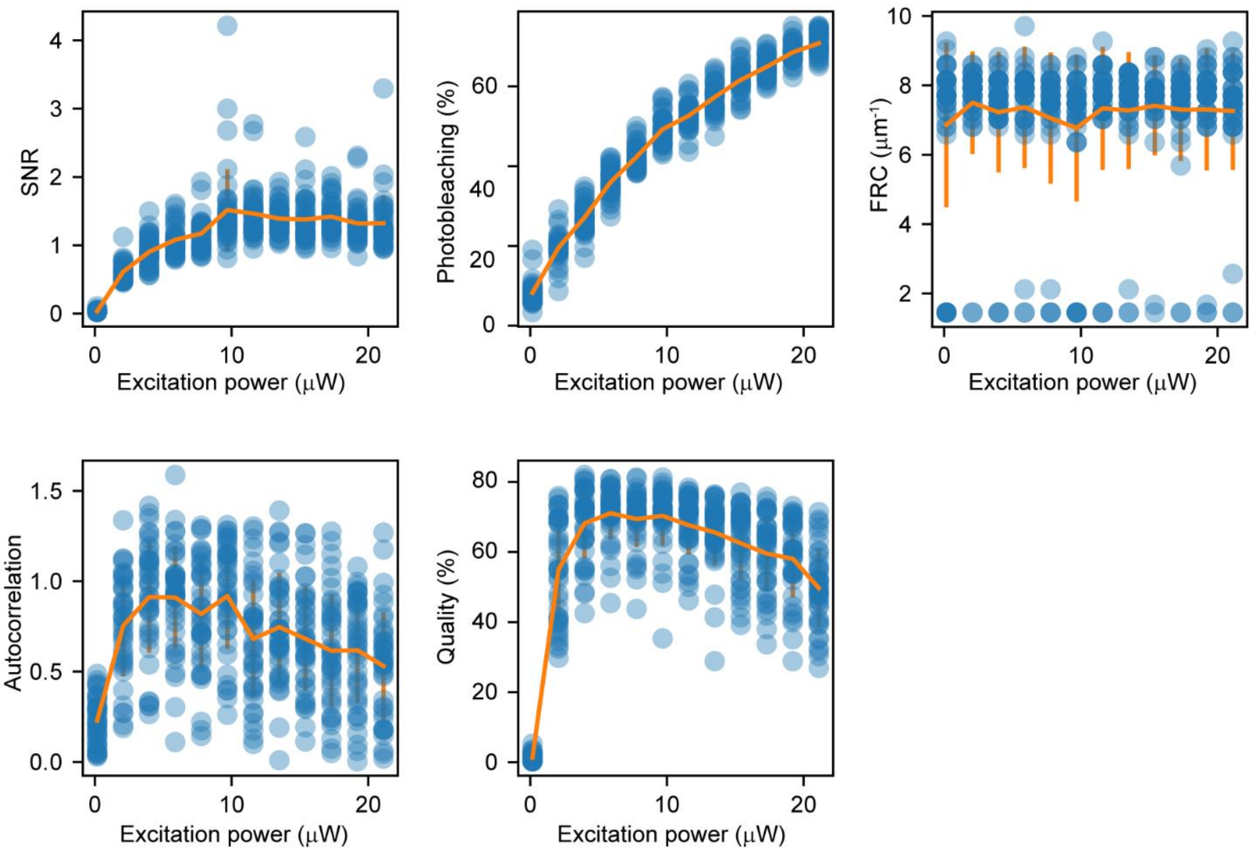

Supplementary Figure 2: Measurement evaluation in the dataset of 468 axonal periodic lattice images used for the replay experiment. Each point correspond to a measurement of the given objective at the given excitation power. A darker color indicates that several points overlap.

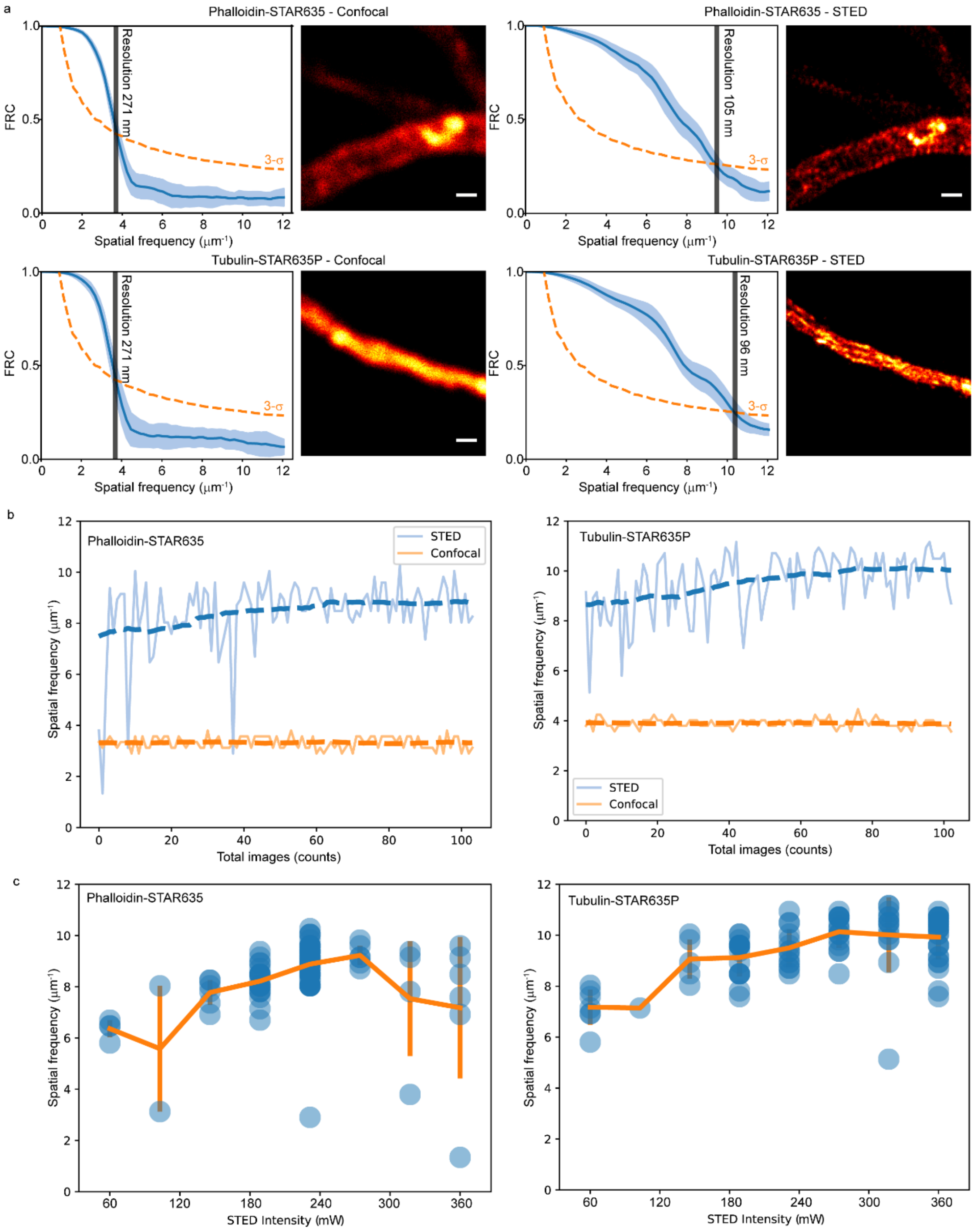
a
Phalloidin-STAR635 - Confocal
Phalloidin-STAR635 - STED
Tubulin-STAR635P - Confocal
Tubulin-STAR635P - STED
FRC
Spatial frequency (µm-1)
Resolution 271 nm
Resolution 105 nm
Resolution 271 nm
Resolution 96 nm
3-σ
b
Phalloidin-STAR635
Tubulin-STAR635P
STED
Confocal
Spatial frequency (µm-1)
Total images (counts)
c
Phalloidin-STAR635
Tubulin-STAR635P
Spatial frequency (µm-1)
STED Intensity (mW)

Supplementary Figure 3: Evaluation of the FRC measurements on our experimental datasets

a) Typical examples obtained with the FRC measurements. The blue line is the averaged trace of the 6 possible pixel combinations (see Methods) and the pale blue region is the standard deviation. b) Evolution of the STED and confocal images resolution throughout the optimization. Raw data (pale lines) is smoothed using a moving average filter with a half width of size 25, showing an increase of STED images resolution along time (dotted lines). c) Resolution of STED images given by a FRC as a function of the STED intensity. Orange lines represent the average and standard deviation of every STED intensity distribution. Scale bars 500 nm.

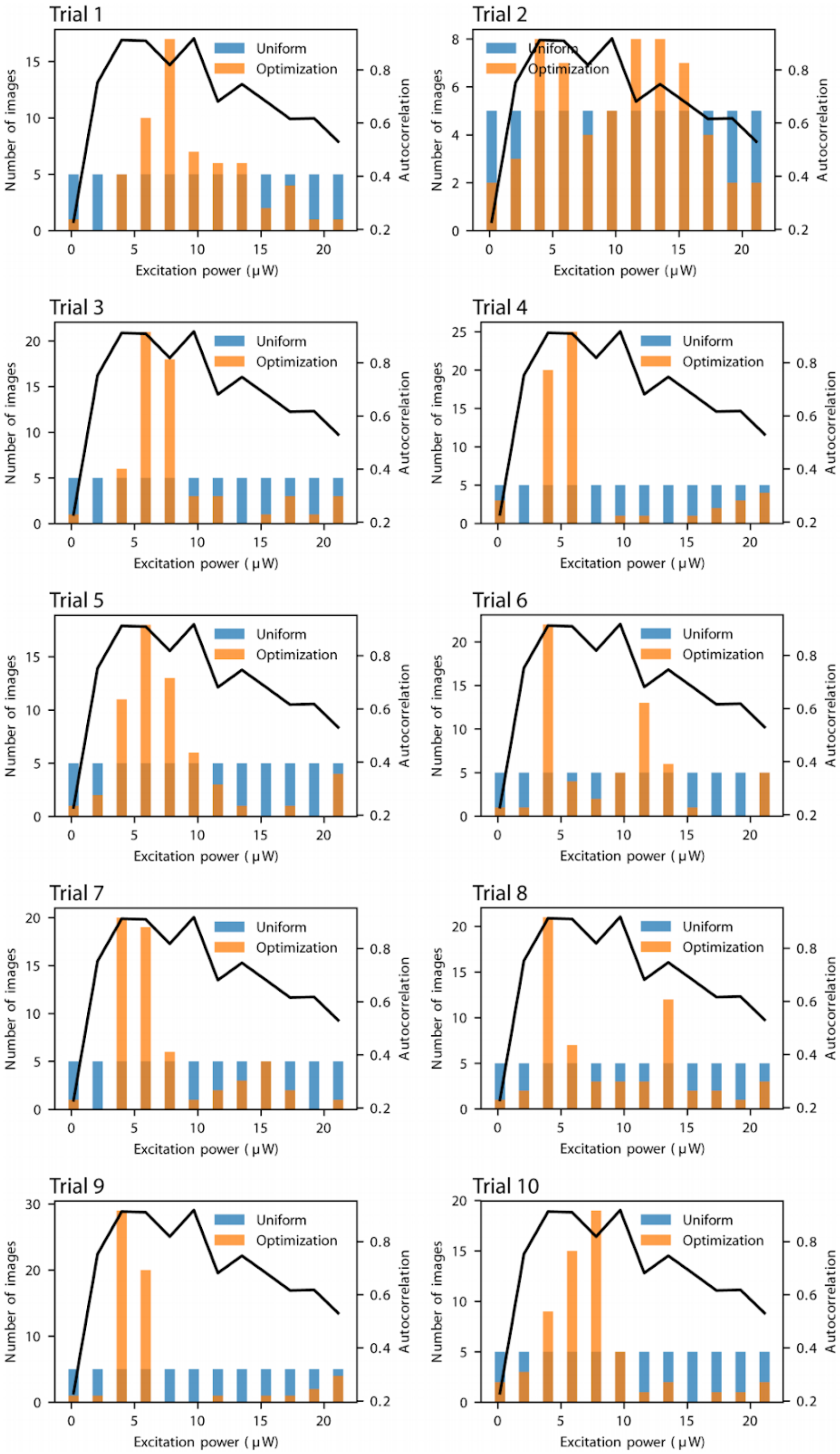

Supplementary Figure 4: Distribution of the 60 images obtained in the 10 first replay trials for grid search and Kernel TS optimization when evaluating the autocorrelation.

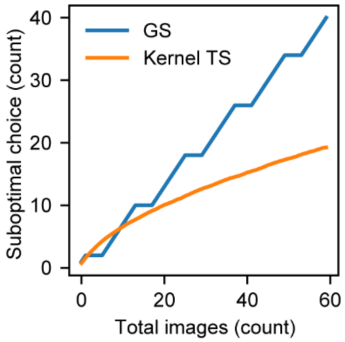

Supplementary Figure 5: Average cumulative regret of images acquired with parameters outside the relevant region (given targeted function) when optimizing the autocorrelation in the replay experiment. Note that the stairs pattern is due to the fact that all parameters inside the relevant region incur no regret.

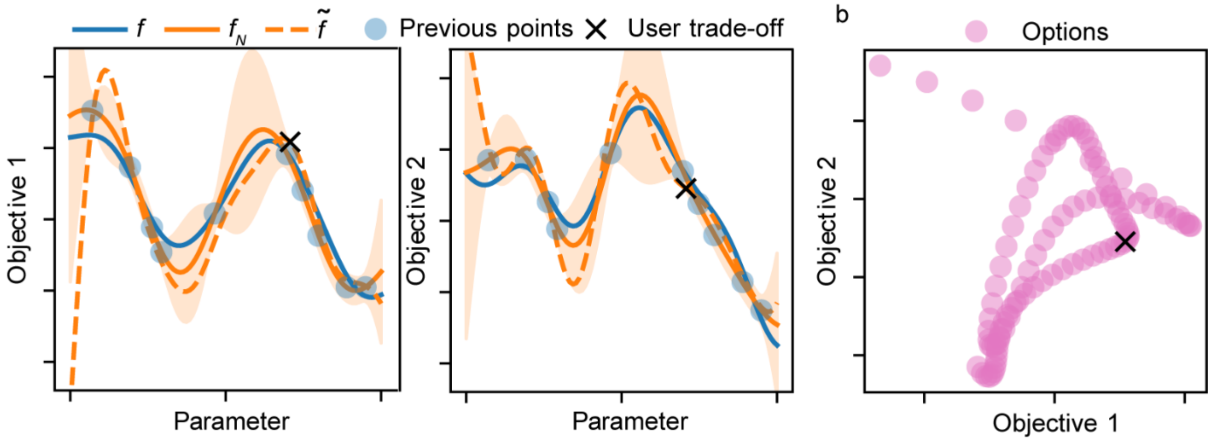

Supplementary Figure 6: Kernel TS for multi-objective optimization a) Examples of sampled objective functions (orange dotted line) from the posterior distribution (mean as orange line, standard deviation as orange area) associated with two objectives (blue line) given N previous observations. b) Multi-objective options resulting from combining the two sampled functions in a). The cross indicates a possible option that could be selected on b) and the corresponding parameters and objective values for each sampled function on a).

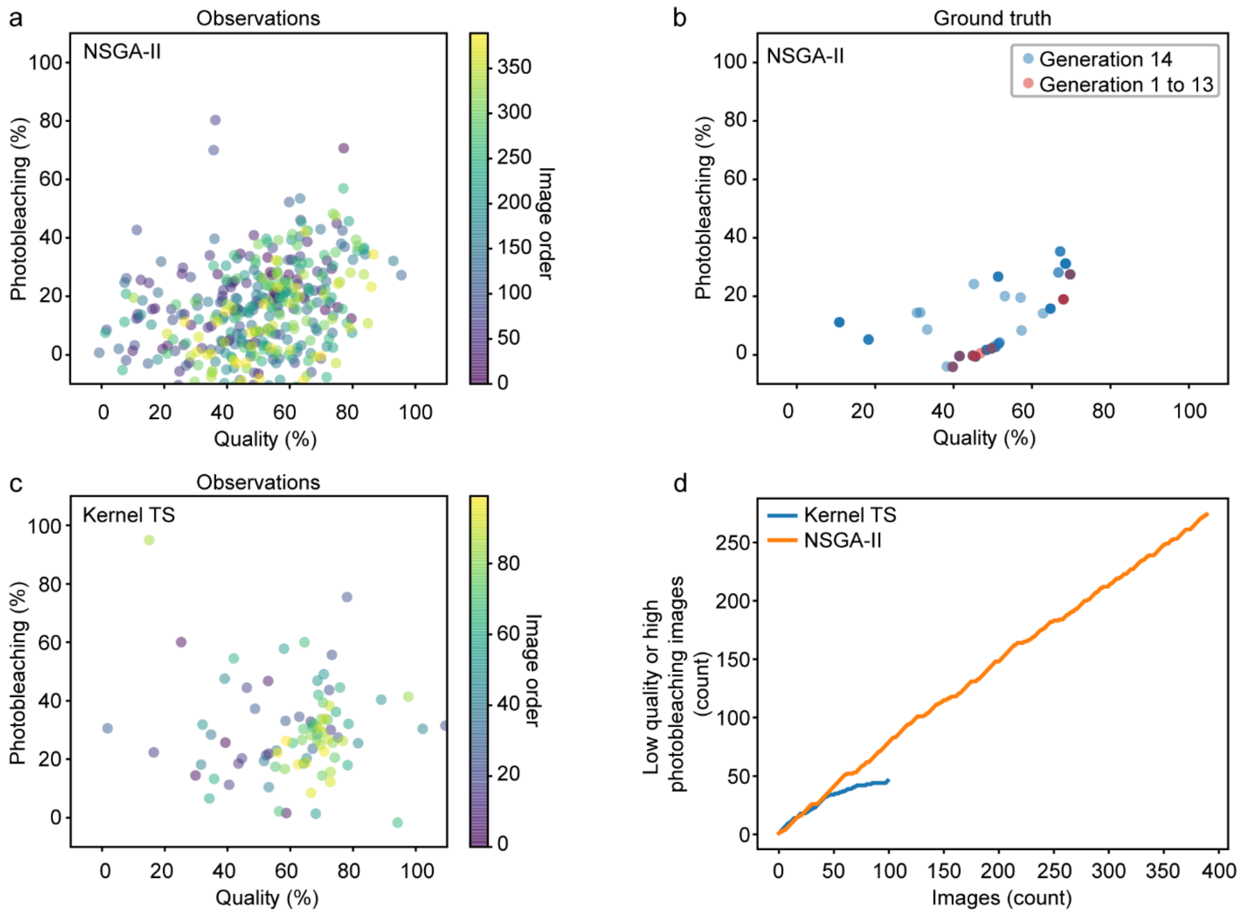

Supplementary Figure 7: Simulation results of NSGA-II and Kernel TS optimization. We simulated a three parameters/two objectives experiment using previously acquired data of the actin cytoskeleton stained with phalloidin-STAR635. a) Evolution of objective values obtained in a simulated optimization run with NSGA-II (see Methods - Offline optimization). b) Expected objective values given the simulator (ground truth) for the last generation compared with previous generations. c) Evolution of objective values obtained in a simulated optimization run with Kernel TS. d) Cumulative regret (quantity of either low quality or highly photobleached images) obtained during simulated optimization sequences shown in a) and c).

Figure interpretation: Results show that the extensive search for the Pareto front prevents NSGA-II from simultaneously increasing the number of good images acquired during the search.

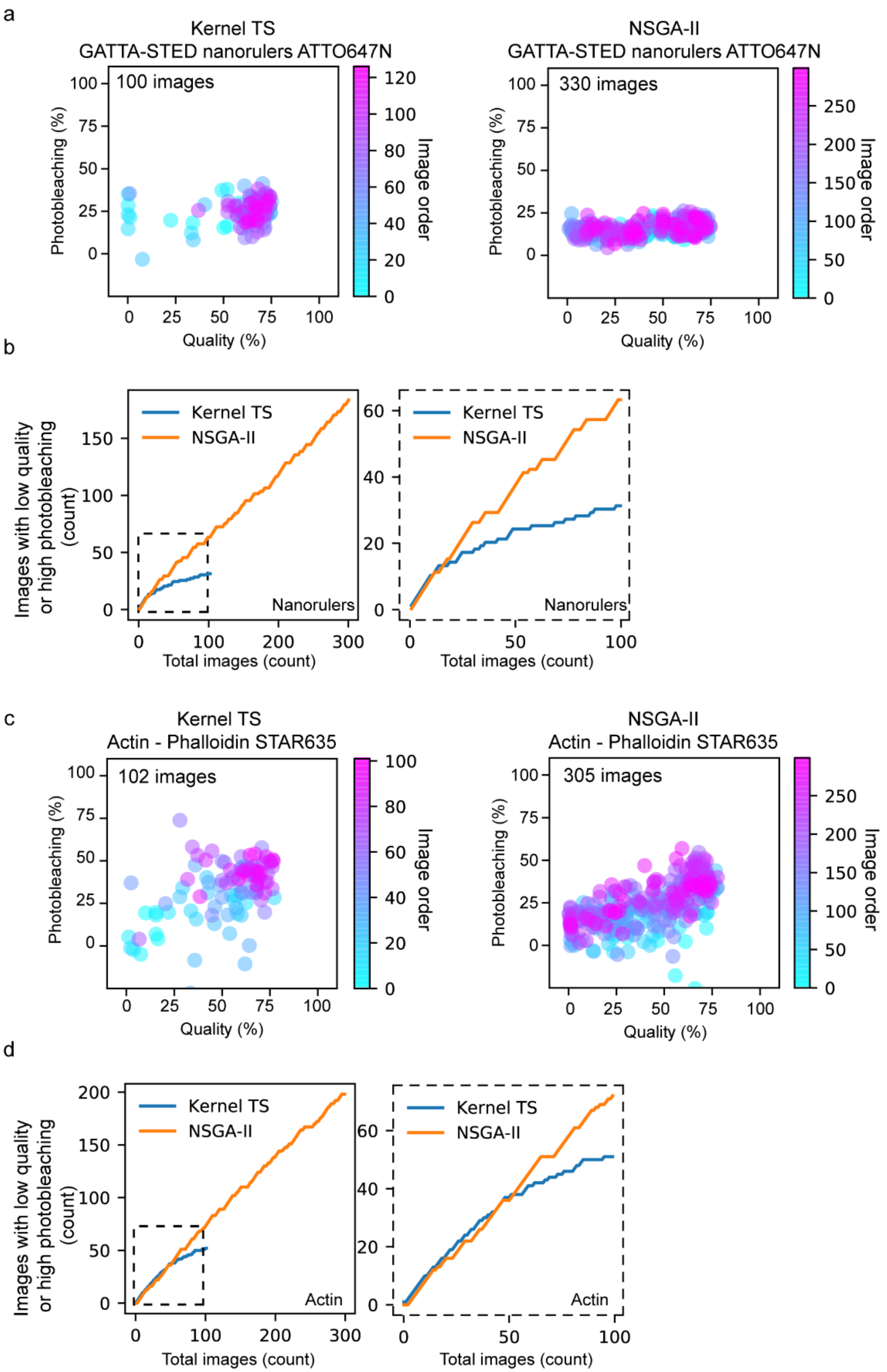
a
Kernel TS
GATTA-STED nanorulers ATTO647N
100 images
Photobleaching (%)
Quality (%)
Image order
NSGA-II
GATTA-STED nanorulers ATTO647N
330 images
b
Kernel TS
NSGA-II
Images with low quality or high photobleaching (count)
Total images (count)
Nanorulers
c
Kernel TS
Actin - Phalloidin STAR635
102 images
NSGA-II
Actin - Phalloidin STAR635
305 images
d
Actin

Supplementary Figure 8: Comparison between Kernel TS and NSGA-II optimization on GATTA-STED nanorulers (70 nm) ATTO 647N and actin stained with phalloidin-STAR635. We conducted real NSGA-II and Kernel-TS optimization experiments with three parameters (i.e.~excitation laser power, depletion laser power and pixel dwell time) and two objectives (i.e.~maximizing the image quality and minimizing photobleaching). For this experiment, we tested two types of samples: 1) the actin cytoskeleton labeled with phalloidin-STAR635 in fixed hippocampal neurons and 2) a reference sample in super-resolution microscopy, GATTA-STED Nanorulers (70nm) labelled with ATTO647N. a) Evolution of objective values over the image sequence of GATTA-STED nanorulers acquired during optimization runs with Kernel TS and NSGA-II. b) Cumulative regret (quantity of either low quality (below 60 %) or highly photobleached images (more than 40 %)) obtained during optimization sequences shown in a). c) Evolution of objective values over the sequence of images of actin stained with phalloidin-STAR635 acquired during optimization runs with Kernel TS and NSGA-II. d) Cumulative regret (quantity of either low quality (below 60 %) or highly photobleached images (more than 60 %)) obtained during optimization sequences shown in c). Low quality corresponds to images that obtained a score below 60%.

Figure interpretation: In our experiments using GATTA-STED Nanorulers, Kernel TS generated 69 out of 100 images with high image quality (above 60%) in comparison with only 37 for NSGA-II. When imaging the actin cytoskeleton tagged with Phalloidin STAR635, Kernel TS generated 46 high quality images out 100 images, while it was only 27 for NSGA-II. Even when pursuing the NSGA-II optimization up to 330 images for the GATTA-STED Nanorulers, only 129 (39%) high quality images were obtained, and for the actin cytoskeleton it was only 100 (33%) out of 300 images.

Experimental results corroborate previous simulation results (Supplementary Fig. 7) showing that by diluting its attention on the whole Pareto front, NSGA-II is not able to maximize the acquisition of good images while simultaneously searching the parameters space. Moreover, while the number of images required by NSGA-II is much lower than GS, it is still much higher than Kernel TS (see Methods). Therefore, this makes NSGA-II unsuitable for live-cell imaging or multi-modal experiments. This strongly supports the use of online, as opposed to offline, optimization for tuning parameters *on a regular basis*, enabling the system to adapt to the imaging task at hand and to biological context.

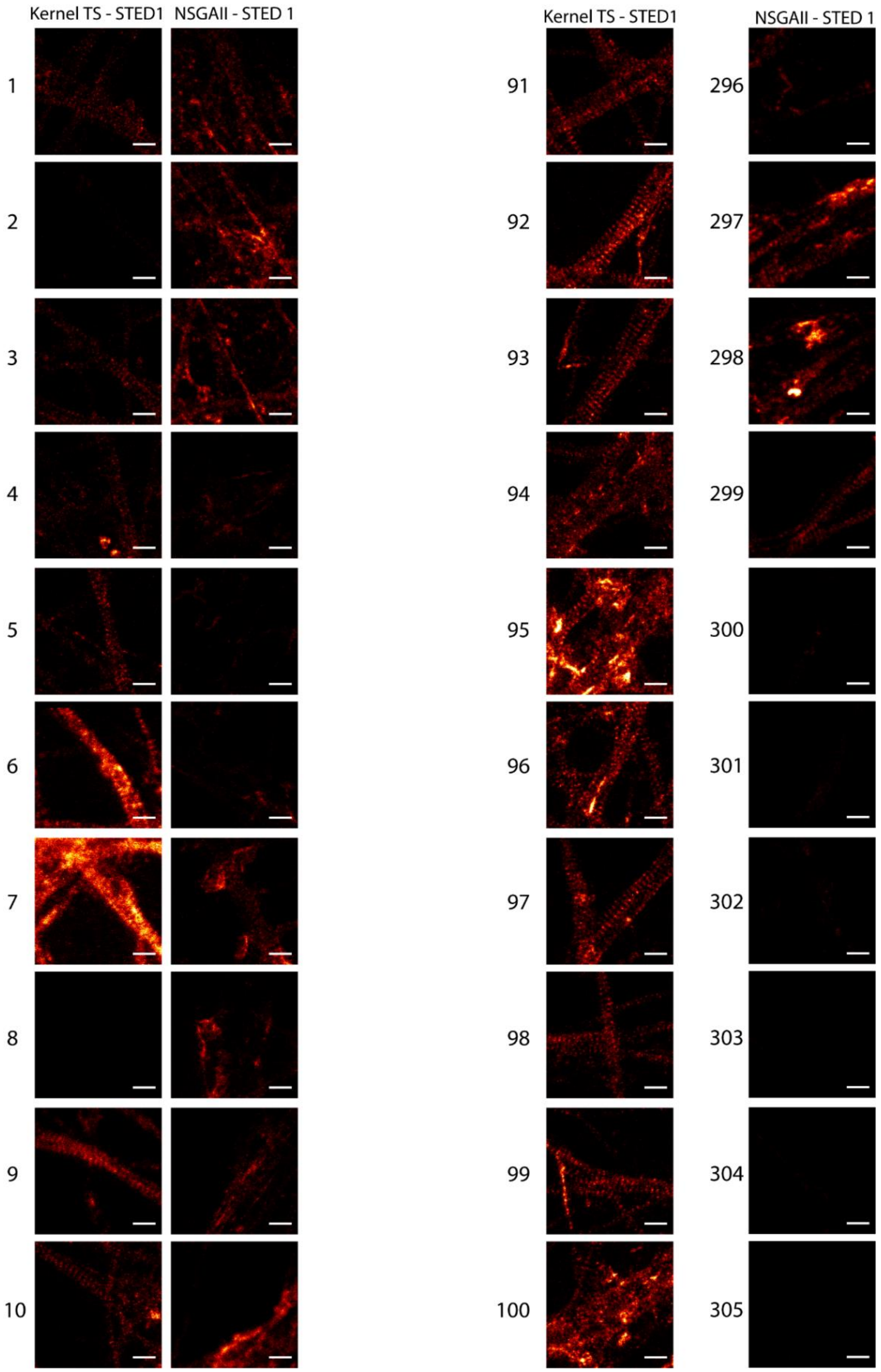

Supplementary Figure 9: Example images obtained at the beginning and at the end of Kernel TS and NSGA-II optimization for the same sample of fixed neurons stained with Phalloidin-STAR635. Note the improvement in image quality (higher signal to noise ratio and higher resolution) between images 1 to 10 and 91 to 100 in the Kernel TS optimization sequence. For NSGA-II optimization, even after 300 images, very low signal is obtained (images 300 to 305). Scale bars 1 µm. The intensity scale was kept constant for all presented STED images.

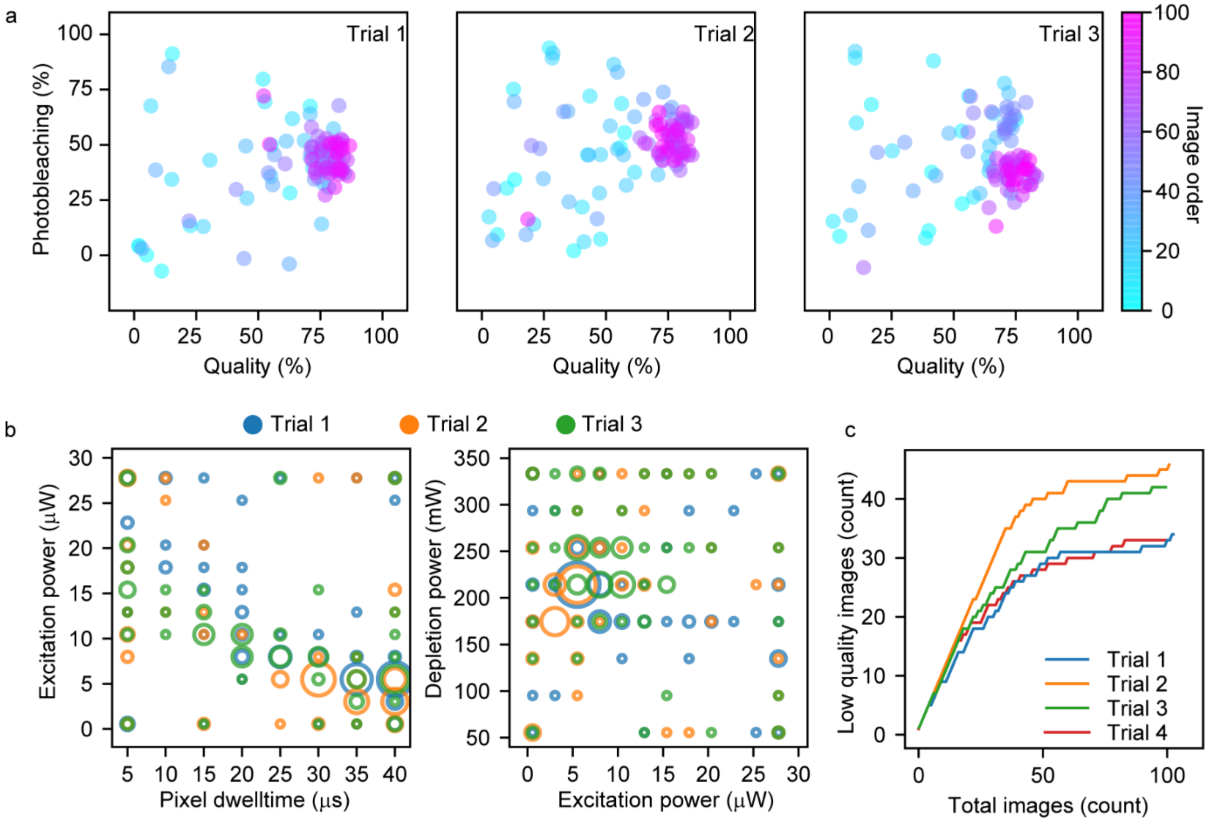

Supplementary Figure 10: Results for multiple optimization trials of the actin cytoskeleton labelled with phalloidin-STAR635. a) Evolution of the objectives along three optimization trials (on three different days). b) Parameters selected during the optimization trials from a). c) Cumulative regret of image quality for four optimization trials (on three different days).

Figure interpretation: We characterized the repeatability of the proposed online optimization approach based on Kernel TS by conducting independent optimization sequences on different samples of the actin cytoskeleton labeled with phalloidin-STAR635 in fixed hippocampal neurons. We observed that all optimization sequences led to sublinear trend of the cumulative regret curve of image quality, and to good trade-offs between image quality and photobleaching.

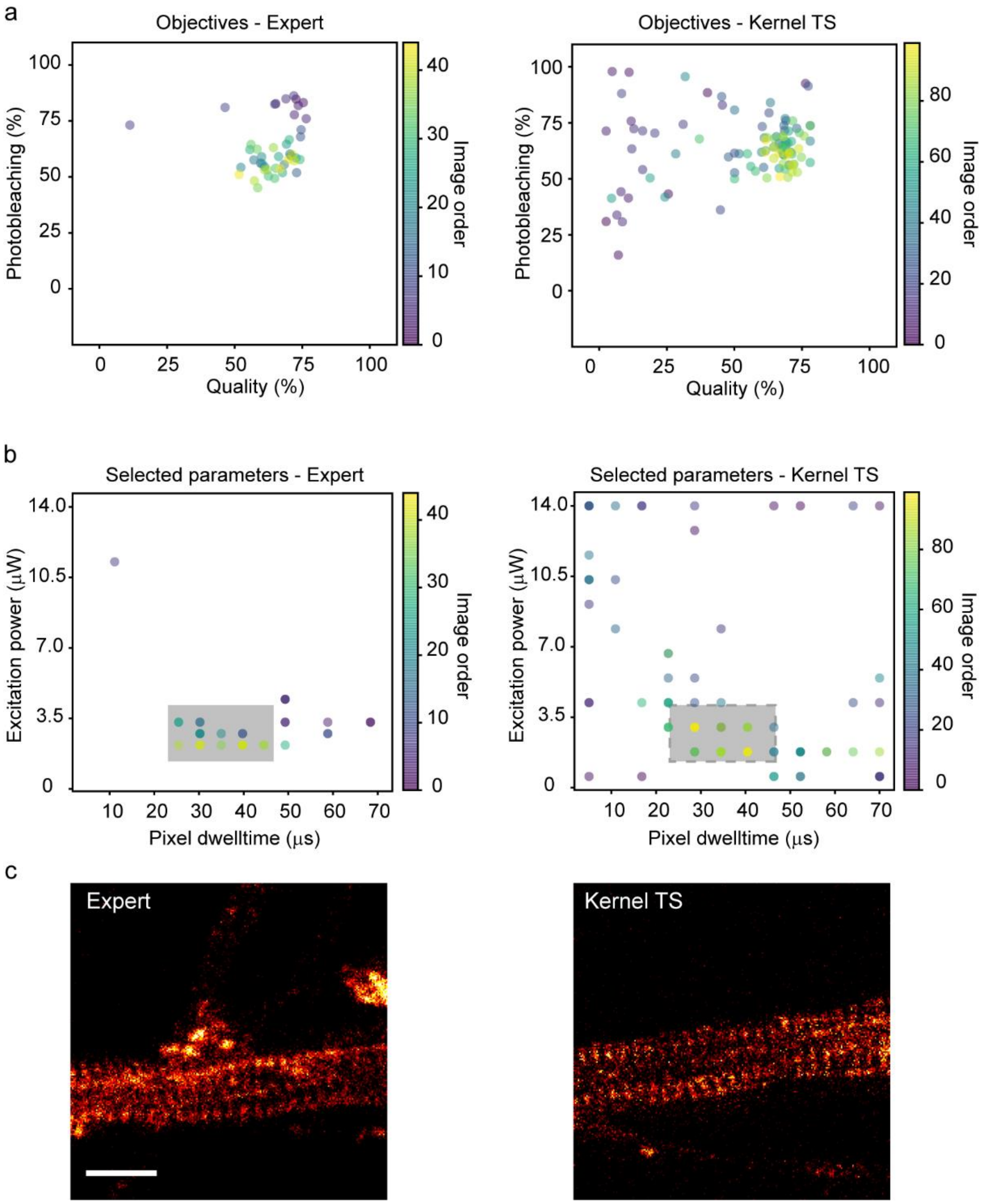

Supplementary Figure 11: Benchmarking Kernel TS optimization with expert user. Results in the objective space and selection of parameters in time for parameter combinations manually selected by an expert user or by user-driven Kernel TS optimization for the imaging of actin tagged with phalloidin-Oregon Green 488. a) User-driven Kernel TS is able to achieve objectives trade-offs similar as what an informed expert would get given prior knowledge of the task. b) During the optimization sequence Kernel TS converges to a similar region (yellow points in the rectangular gray areas) in the parameter space as the one that was identified by an expert to obtain the best trade-offs, even though Kernel TS had to search a much wider space. c) Example of good images of the actin cytoskeleton that were obtained during the manual exploration by an expert user (left) and towards the end of Kernel TS optimization (image 80 out of 100). Scale bar: 1 μm

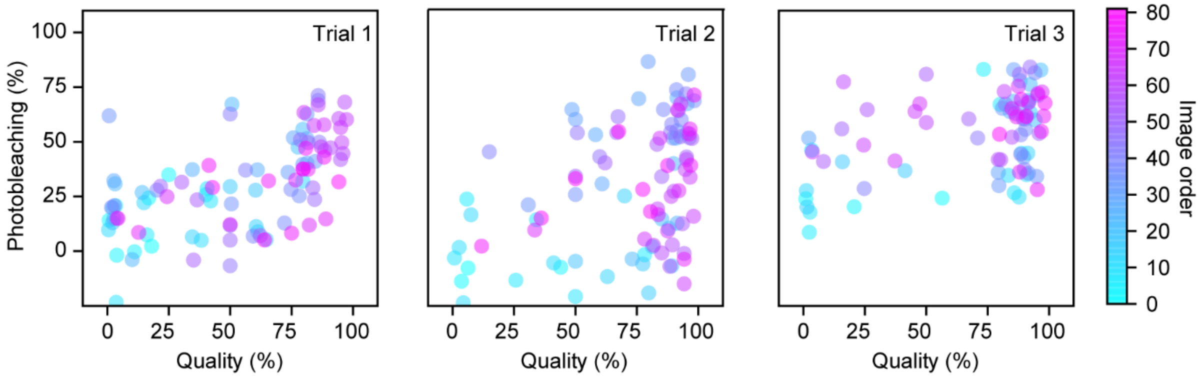

Supplementary Figure 12: Optimization results in the objectives space for the three different optimization trials of CaMKII-SNAP-SiR in living neuron shown in Fig. 2. Objective evaluation for the 80 images of Kernel TS optimization sequences. Due to biological variability, the possible trade-offs between objectives vary strongly between trials.

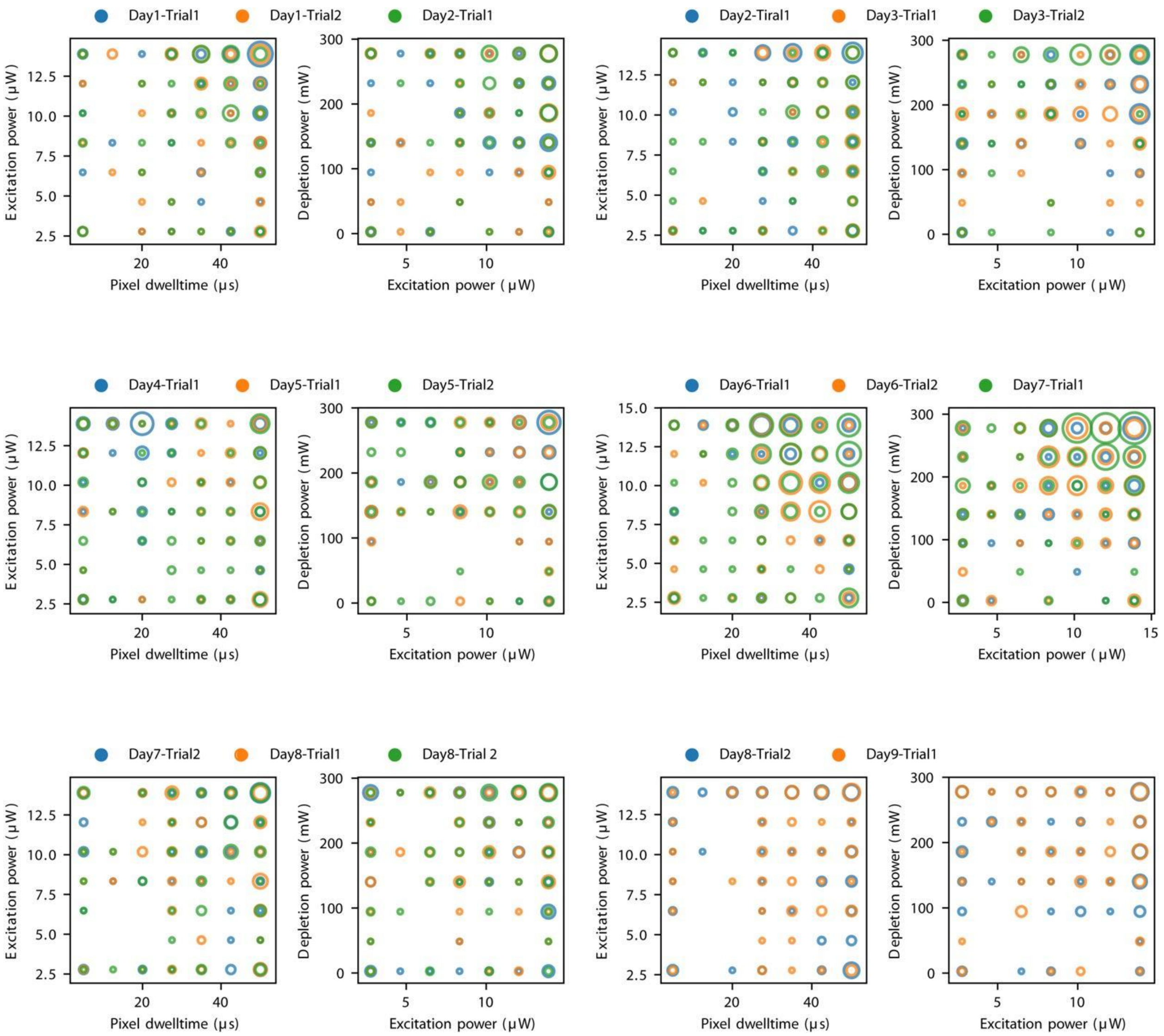

Supplementary Figure 13: Parameters selected by Kernel-TS for 17 optimization trials of CaMKII-SNAP-SiR in living neurons. Variations among the parameters that lead to the best objective trade-offs, depending on the neurons and culture preparation.

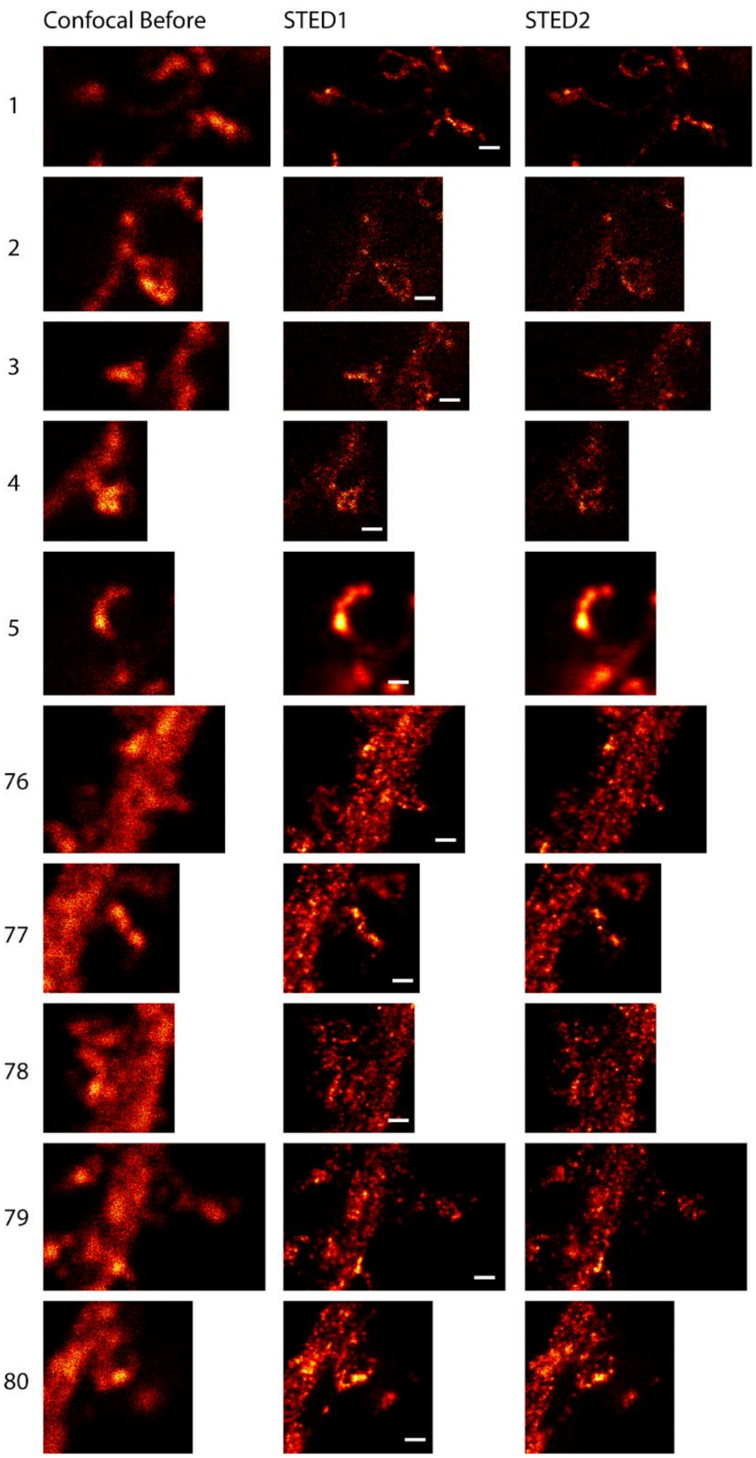

Supplementary Figure 14: Examples of original confocal and STED images obtained at the beginning and the end of live-cell multi-objective optimization for the protein αCaMKII-SNAP-SiR. Note the improvement in image quality (higher signal to noise ratio and higher resolution) between images 1 to 5 and 76 to 80 during the Kernel TS optimization sequence. Nanometric clusters of α CaMKII-SNAP-SiR inside spines and dendrites are detected in images 76 to 80 and in image 1. The intensity scale was kept constant for all presented STED images. Scale bars 500 nm.

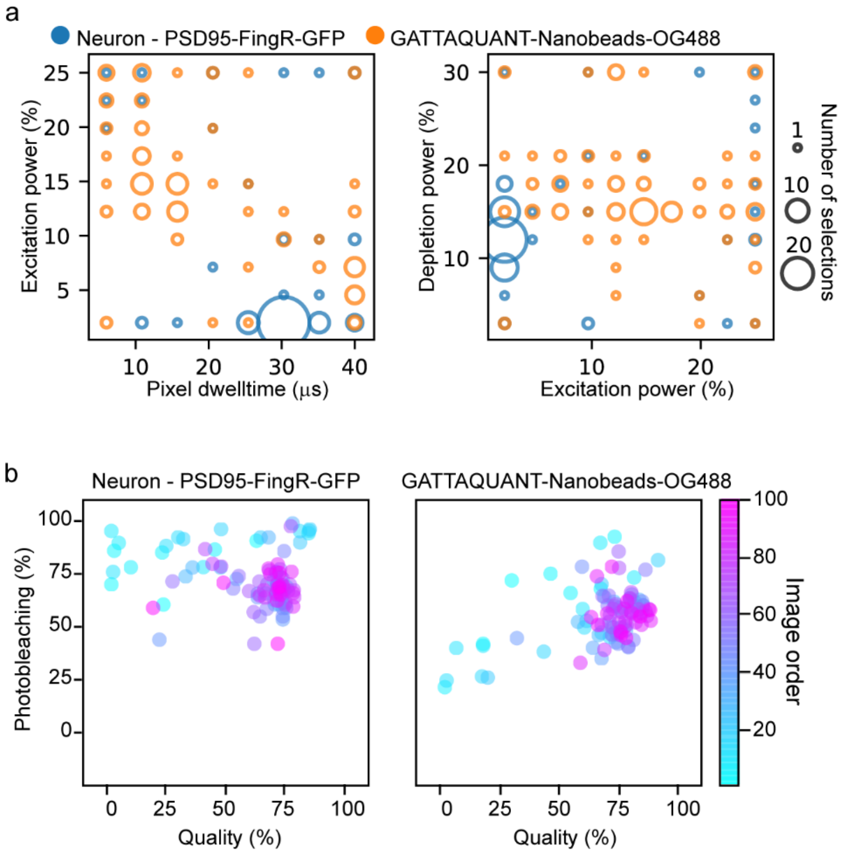

Supplementary Figure 15: Kernel TS optimization on two reference samples. Three objective (image quality, photobleaching, imaging speed) and three parameter (excitation laser power, depletion laser power and pixel dwell time) Kernel TS optimization was performed on PSD95-FingR-GFP in living neuron and GATTAQUANT Nanobeads labelled with Oregon Green 488. The previous knowledge acquired in those optimization sequences was used as a reference to evaluate whether it could accelerate the optimization process on other samples. a) Parameter combinations that were sampled with Kernel TS optimization for PSD95-FingR-GFP (blue) and GATTAQUANT Nanobeads OG488 (orange). Shown are two planes of the three dimensional parameter space. b) Evolution of the quality and photobleaching objectives for the 100 images taken during Kernel TS optimization.

Figure interpretation: While short pixel dwell time (between 10 and 20μs) with high excitation power were optimal for the Nanobeads, longer pixel dwell time with low excitation power were necessary for PSD95-FingR-GFP to obtain a good trade-off between image quality and bleach as showed in b). In comparison to PSD95-FingR-GFP, slightly higher image quality and lower photobleaching was achievable for the Nanobeads. We used these two optimization trials in the following experiments on GFP imaging optimization as the reference samples (Supplementary Figures 18-31).

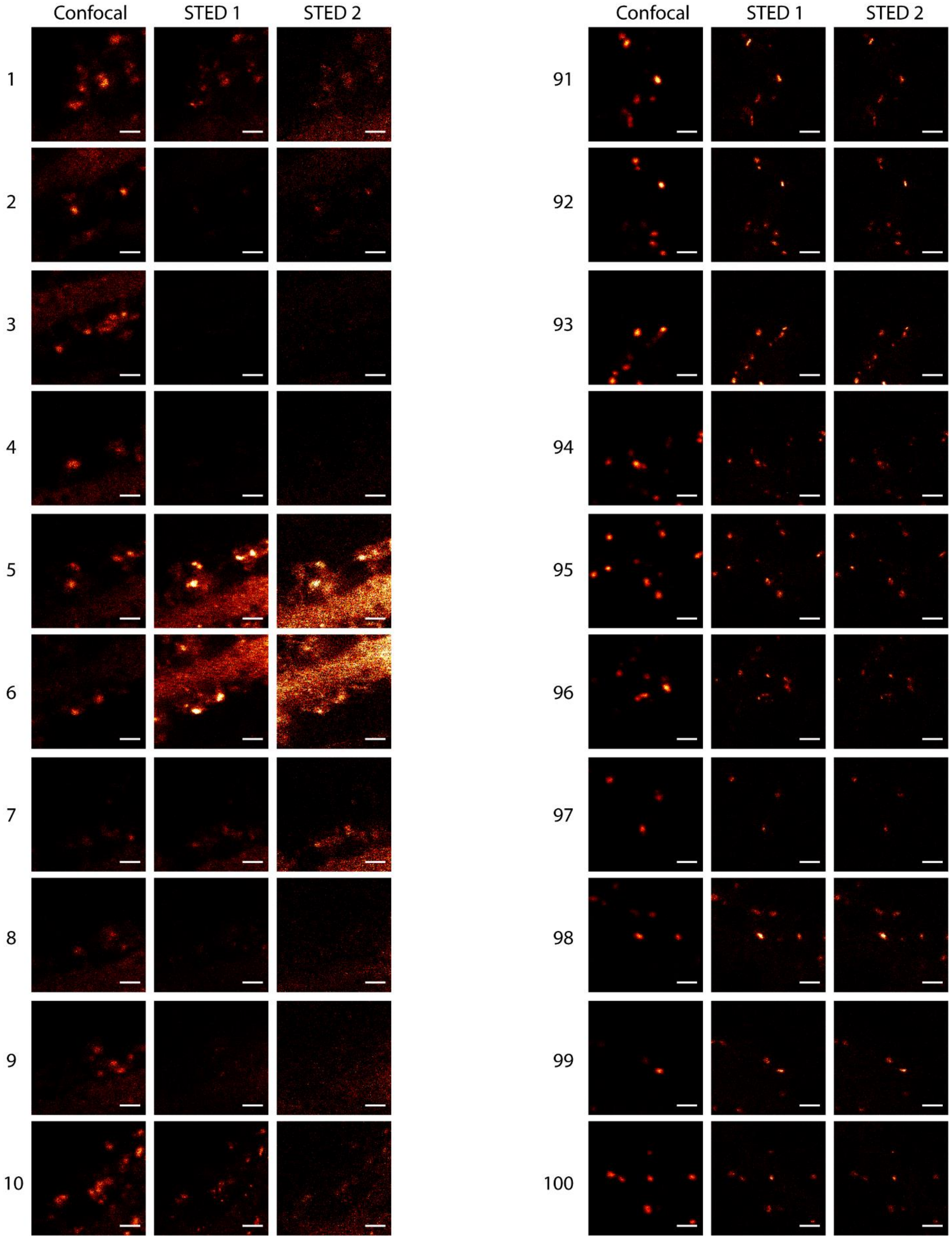

Supplementary Figure 16: Example images obtained in one Kernel TS optimization sequence of PSD95-FingR-GFP in living neurons. Note the improvement in image quality (higher signal to noise ratio and higher resolution) and photobleaching between images 1 to 10 and 91 to 100 during the Kernel TS optimization sequence. For images 91 to 100, two high quality images with nanometric PSD95 clusters are obtained. Scale bars 1 µm. The intensity scale was kept constant for all presented STED images.

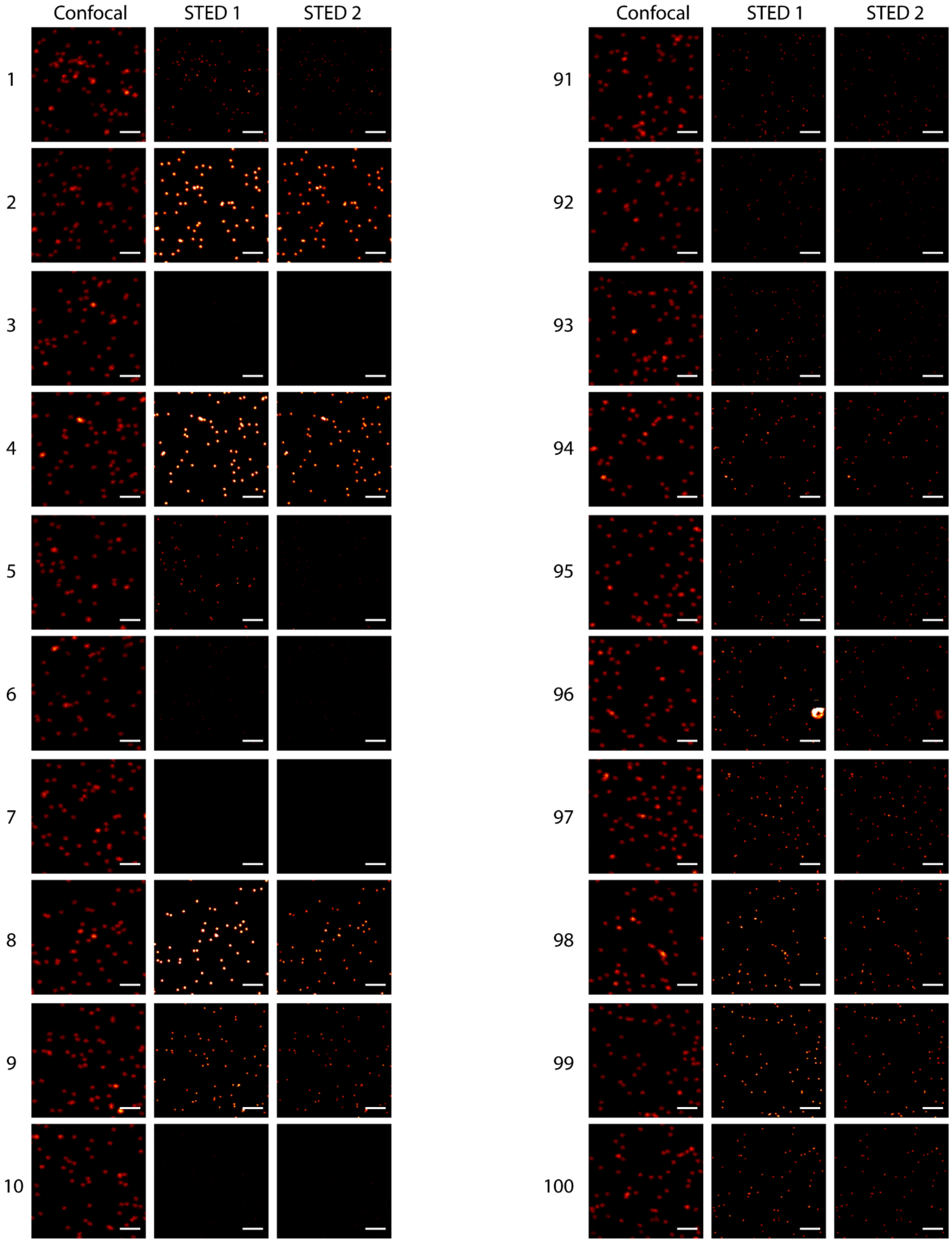

Supplementary Figure 17: Example images of GATTA-Beads OG488 during one optimization sequence using Kernel TS. Note the improvement in image quality (higher signal to noise ratio and higher resolution) between images 1 to 10 and 91 to 100 during the Kernel TS optimization sequence. Scale bars 1 µm. The intensity scale was kept constant for all presented STED images.

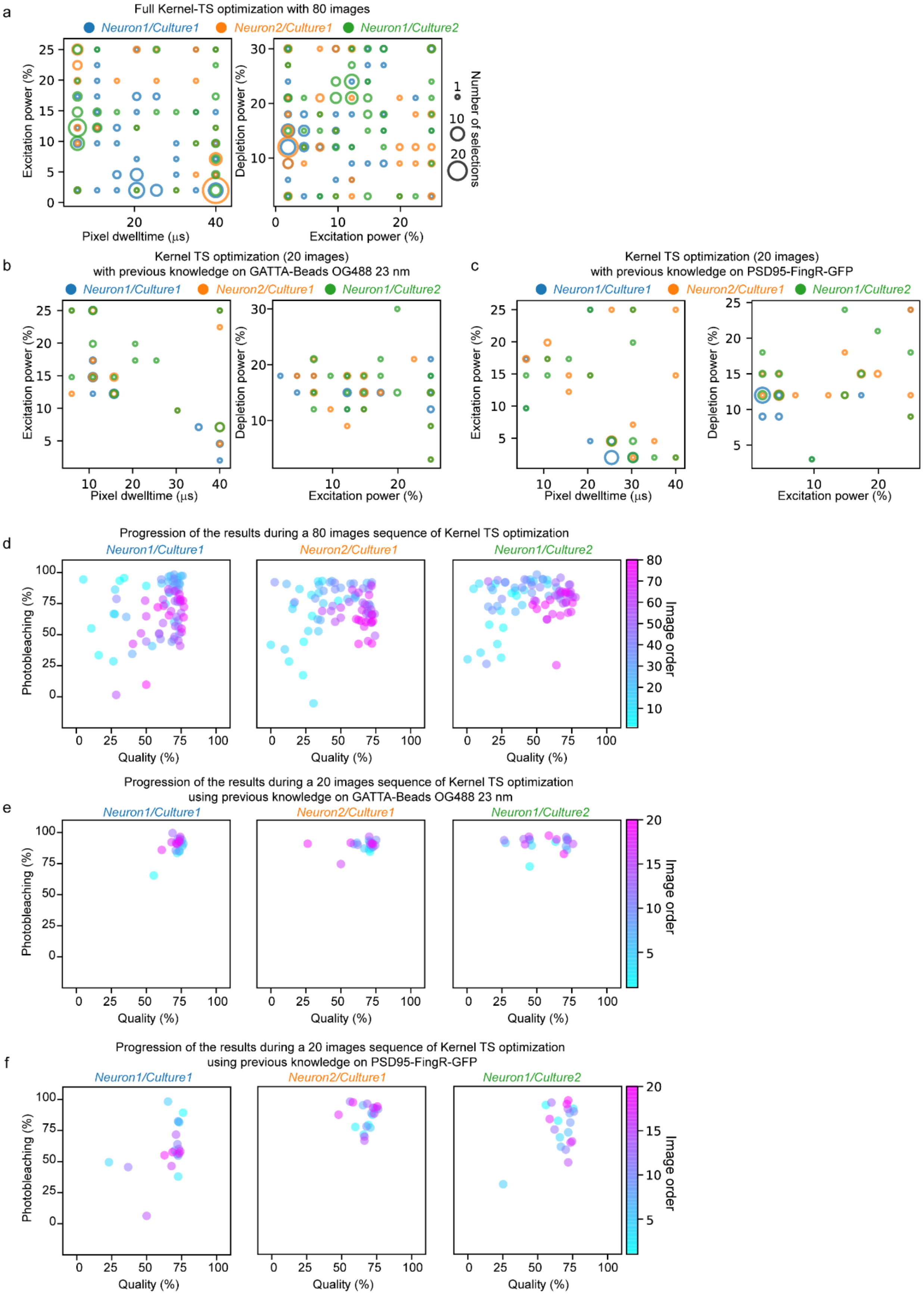

a
Full Kernel-TS optimization with 80 images
Neuron1/Culture1
Neuron2/Culture1
Neuron1/Culture2
Excitation power (%)
Pixel dwelltime (µs)
Depletion power (%)
Excitation power (%)
Number of selections
1
10
20
b
Kernel TS optimization (20 images)
with previous knowledge on GATTA-Beads OG488 23 nm
c
Kernel TS optimization (20 images)
with previous knowledge on PSD95-FingR-GFP
d
Progression of the results during a 80 images sequence of Kernel TS optimization
Photobleaching (%)
Quality (%)
Image order
e
Progression of the results during a 20 images sequence of Kernel TS optimization
using previous knowledge on GATTA-Beads OG488 23 nm
f
Progression of the results during a 20 images sequence of Kernel TS optimization
using previous knowledge on PSD95-FingR-GFP

Supplementary Figure 18: Kernel TS optimization on GFP-α CaMKII for three independent neurons with and without previous knowledge.

Three objective (image quality, photobleaching, imaging speed) and three parameter (excitation laser power, depletion laser power and pixel dwell time) Kernel TS optimization was performed on GFP-α CaMKII in living neurons. The results obtained for three different neurons from two separate cultures are compared. a) Imaging parameter combinations during Kernel TS optimization trial (80 images, living neurons) without previous knowledge. b,c) Imaging parameter combinations selected for 20 images that were acquired using the previous knowledge obtained with the reference samples GATTAQUANT Nanobeads OG 488 (b) or PSD95-FingR-GFP in living neurons (c) (Supplementary Figure 15). d) Evolution of the quality and photobleaching objectives for the 80 images taken during Kernel TS optimization, without previous knowledge (the parameters used to obtain those results are shown in panel a). e,f) Evolution of the quality and photobleaching objectives for 20 images taken during Kernel TS optimization, using the previous knowledge obtained during Kernel TS optimization on the reference samples GATTAQUANT Nanobeads OG 488 (e) or PSD95-FingR-GFP in living neurons (f) (Supplementary Figure 15). See Methods for a detailed description of the experimental design and procedures.

Figure interpretation: Panel a) shows that the required pixel dwell time and excitation power that led to good quality images combined to reduced photobleaching varied considerably from one neuron to another. For *Neuron1/Culture2* (green), moderate excitation power (10-20 % - 5.2-10.7 μW) with short pixel dwell time (6 μs) resulted in the best trade-offs (image quality, photobleaching and imaging speed). For *Neuron2/Culture1* (blue) low excitation power (1 % - 0.4 μW) and moderate pixel dwell time (20 μs) resulted in the best trade-offs. Finally, for *Neuron1/Culture1* (orange) longer pixel dwell time (40 μs) and low excitation power (1 % - 0.4 μW) resulted in the best trade-offs. A good trade-off between image quality and photobleaching was reached for the three neurons toward the last 20 images (pink circles in panel d). The image quality was between 60 % and 80 % while the photobleaching was between 50 % and 75 % for most of these last 20 images. The reduced photobleaching enabled two consecutive usable STED images of GFP-α CaMKII in living neurons (e.g. last 10 images of one trial showed in Suppl. Fig. 20). When using the nanobeads as reference (panel e), the image quality was generally high but the photobleaching was almost 100 % for all trials. The parameters that were determined to be optimal for the nanobeads (fast pixel dwell time, high excitation power, and moderate depletion power) were not suitable for the observed neurons tagged with GFP-α CaMKII. Using the previous knowledge acquired with PSD95-FingR-GFP led to better results (panel f) compared to the nanobeads, but for *Neuron2/Culture1* the photobleaching was higher than in the last 20 images of the full optimization run without prior knowledge.

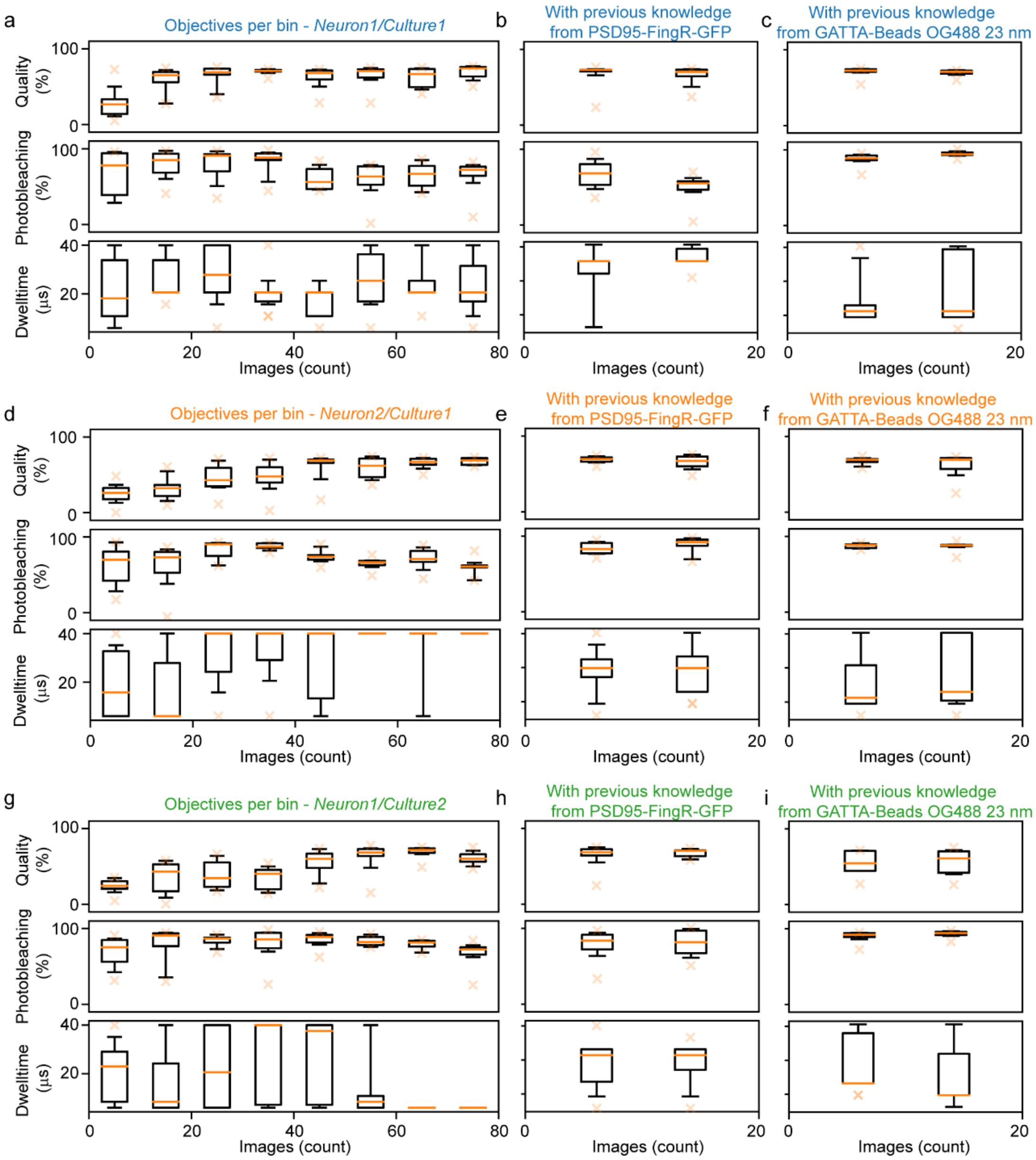

Supplementary Figure 19: Evolution of the objective values during Kernel TS optimization sequences on GFP-α CaMKII-GFP in 3 living neurons with and without previous knowledge. Alternative representation of the data from Supplementary Fig. 18 d,e,f) to show the progression of all three objectives quantitatively. Each box corresponds to the bined objective values of 10 images. a,d,g) Complete sequences of 80 images without previous knowledge. b,e,h) Results obtained for the 20 images that were taken using previous knowledge from the reference sample PSD95-FingR-GFP. c,f,i) Results obtained for the 20 images that were taken using previous knowledge from the reference sample GATTAQUANT Nanobeads OG 488.

Figure interpretation: With this representation we can evaluate all three objectives simultaneously. For each neuron tested, at least one objective was less successful when using prior knowledge, compared to the last 20 images of the full optimization sequence. These results emphasize the limitations of using prior knowledge from reference samples for the presented optimization task. Orange line indicates the median, box covers the 1st to the 3rd quartiles, and whiskers extend from 10th to 90th percentiles.

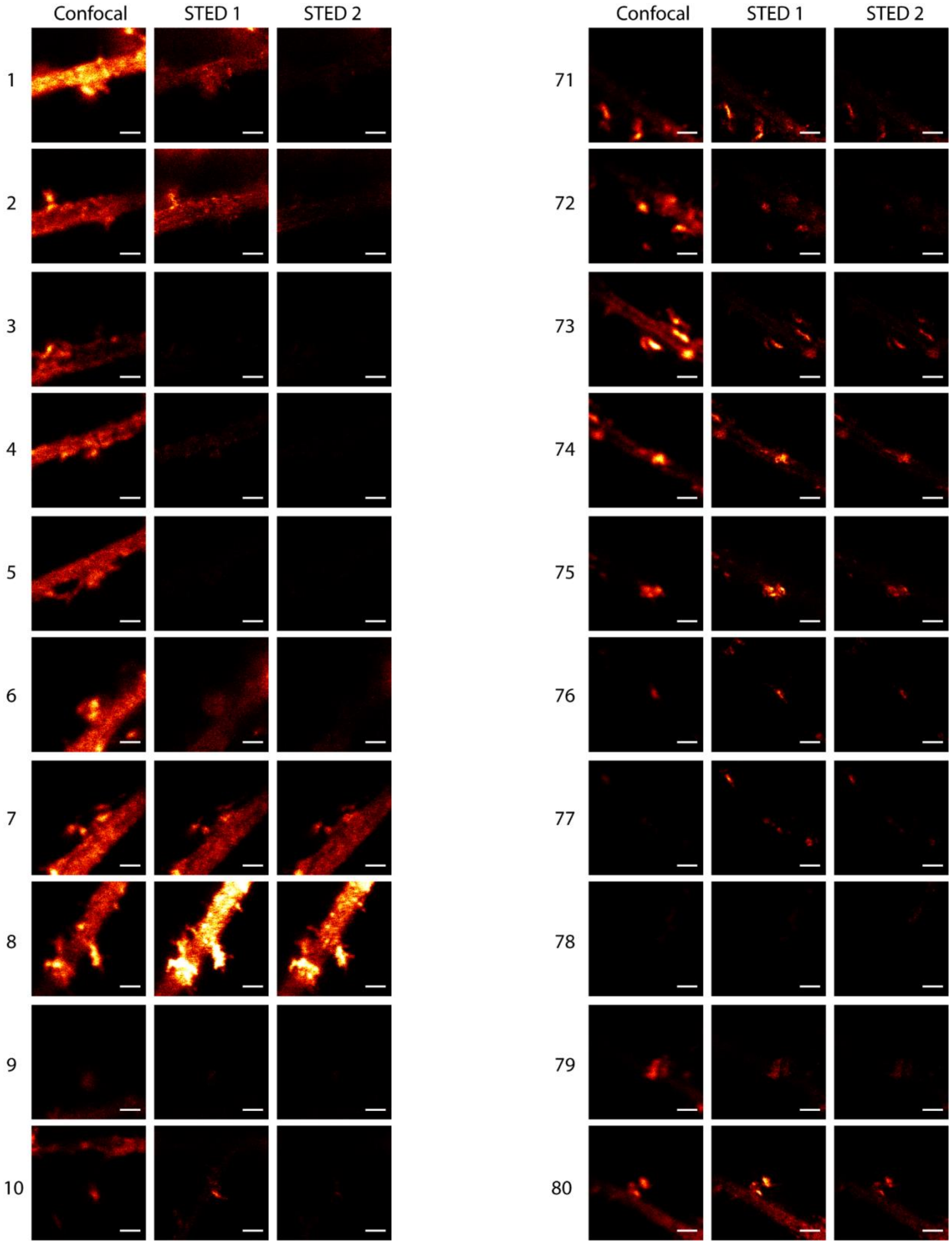

Supplementary Figure 20: Example images obtained during a Kernel TS optimization sequence on GFP-αCaMKII in living neurons. Note the improvement in image quality (higher signal to noise ratio and higher resolution) and photobleaching between images 1 to 10 and 71 to 80 during the Kernel TS optimization sequence. Two high quality images of the distribution of α CaMKII in dendritic spines were obtained for 70% of the last 10 images of the sequence (compared to none of the first 10 images). Scale bars 1 µm. The intensity scale was kept constant for all presented STED images.

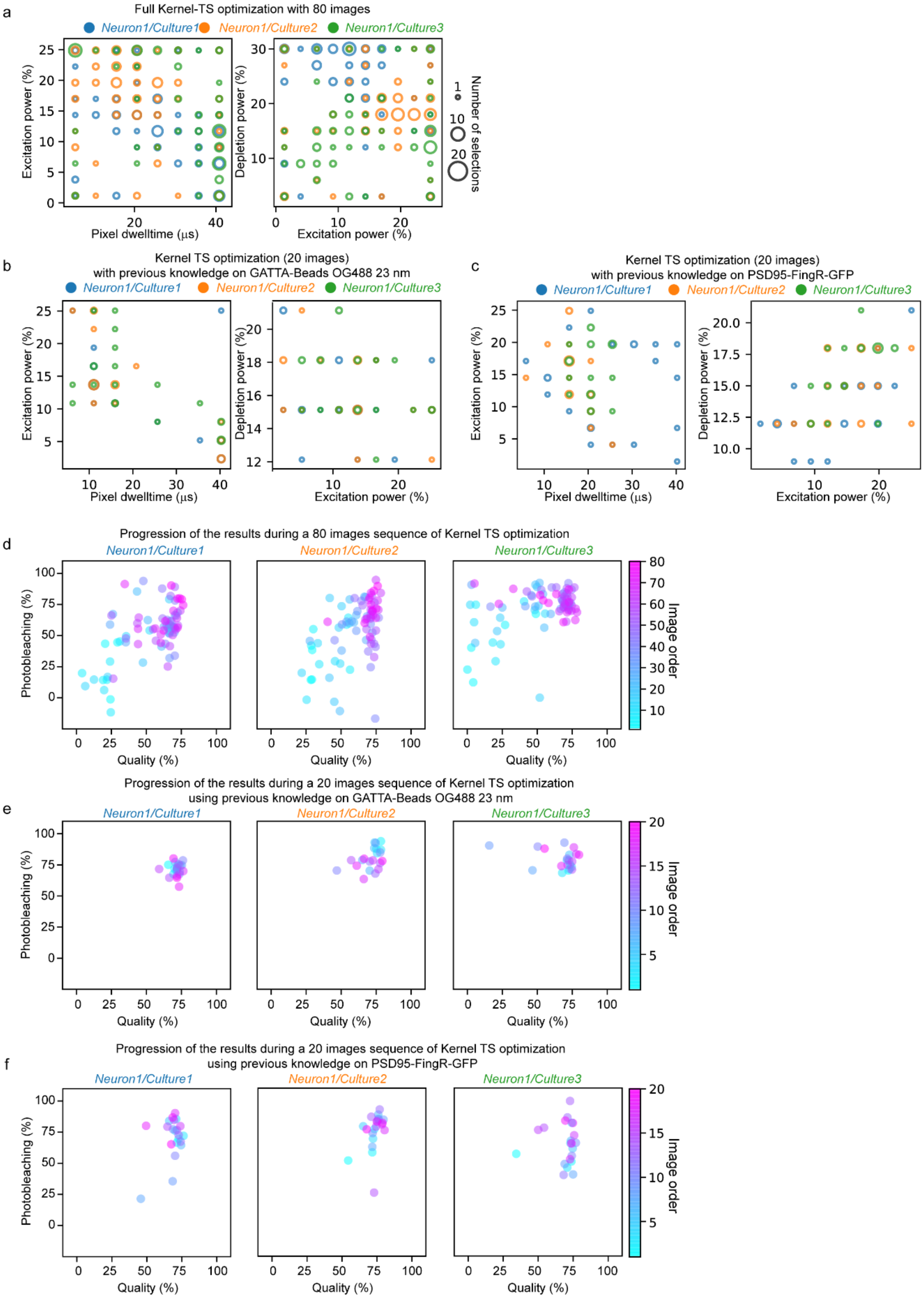

a
Full Kernel-TS optimization with 80 images
Neuron1/Culture1
Neuron1/Culture2
Neuron1/Culture3
Excitation power (%)
Pixel dwelltime (μs)
Depletion power (%)
Excitation power (%)
Number of selections
1
10
20
b
Kernel TS optimization (20 images)
with previous knowledge on GATTA-Beads OG488 23 nm
Neuron1/Culture1
Neuron1/Culture2
Neuron1/Culture3
Excitation power (%)
Pixel dwelltime (μs)
Depletion power (%)
Excitation power (%)
c
Kernel TS optimization (20 images)
with previous knowledge on PSD95-FingR-GFP
Neuron1/Culture1
Neuron1/Culture2
Neuron1/Culture3
Excitation power (%)
Pixel dwelltime (μs)
Depletion power (%)
Excitation power (%)
d
Progression of the results during a 80 images sequence of Kernel TS optimization
Neuron1/Culture1
Neuron1/Culture2
Neuron1/Culture3
Photobleaching (%)
Quality (%)
Image order
e
Progression of the results during a 20 images sequence of Kernel TS optimization
using previous knowledge on GATTA-Beads OG488 23 nm
Neuron1/Culture1
Neuron1/Culture2
Neuron1/Culture3
Photobleaching (%)
Quality (%)
Image order
f
Progression of the results during a 20 images sequence of Kernel TS optimization
using previous knowledge on PSD95-FingR-GFP
Neuron1/Culture1
Neuron1/Culture2
Neuron1/Culture3
Photobleaching (%)
Quality (%)
Image order

Supplementary Figure 21: Kernel TS optimization on LifeAct-GFP for three independent neurons with and without previous knowledge. Three objective (image quality, photobleaching, imaging speed) and three parameter (excitation laser power, depletion laser power and pixel dwell time) Kernel TS optimization was performed on LifeAct-GFP in living neurons. The results obtained for three different neurons from three separate cultures are compared. a) Imaging parameter combinations during a full Kernel TS optimization trial (80 images, living neurons) without previous knowledge. b,c) Imaging parameter combinations selected for 20 images that were acquired using the previous knowledge obtained with the reference samples GATTAQUANT Nanobeads OG 488 (b) or PSD95-FingR-GFP in living neurons (c) (see Supplementary Figure 15). d) Evolution of the quality and photobleaching objectives for the 80 images taken during Kernel TS optimization, without previous knowledge (the parameters used to obtain those results are shown in panel a). e,f) Evolution of the quality and photobleaching objectives for 20 images taken during Kernel TS optimization, using the previous knowledge obtained during Kernel TS optimization on the reference samples GATTAQUANT Nanobeads OG 488 (e) or PSD95-FingR-GFP in living neurons (f) (Supplementary Figure 15). See Methods for a detailed description of the experimental design and procedures.

Figure interpretation: Panel a) shows that the required pixel dwell time, excitation power, and depletion power that led to good quality images combined to reduced photobleaching varied considerably from one neuron to another. For *Neuron1/Culture3* (green), low to moderate excitation power (1-10 % - 0.4-5.2 µW) with long pixel dwell time (40 µs) and relatively low depletion power (12-15% - 40-50 mW) resulted in the best trade-offs (image quality, photobleaching and imaging speed). For *Neuron1/Culture1* (blue) moderate excitation power (12-18 % - 6.4-9.4 µW), high depletion power (22-30 % - 72-99 mW) and moderate pixel dwell time (15-25 µs) resulted in the best trade-offs. Finally, for *Neuron1/Culture2* (orange) moderate pixel dwell time (15-25 µs) but higher excitation power (15-25 % - 8-13 µW) and moderate depletion power (18 % - 59 mW) resulted in the best trade-offs. A good trade-off between image quality and photobleaching was reached for the three neurons toward the last 20 images (pink circles in panel d). The image quality was between 60 % and 80 % while the photobleaching was very variable between the neurons but could be reduced below 75 % for most of these last 20 images. The reduced photobleaching enabled two consecutive usable STED images of LifeAct-GFP in living neuron (e.g. last 10 images of one trial showed in Supplementary Fig. 23). In the case of LifeAct-GFP (but not for GFP-α CaMKII), the use of the previous knowledge acquired with the GATTAQUANT Nanobeads OG 488 lead to acceptable trade-offs (panel e). Using the previous knowledge acquired with PSD95-FingR-GFP led to higher photobleaching for two of the three neurons (panel f).

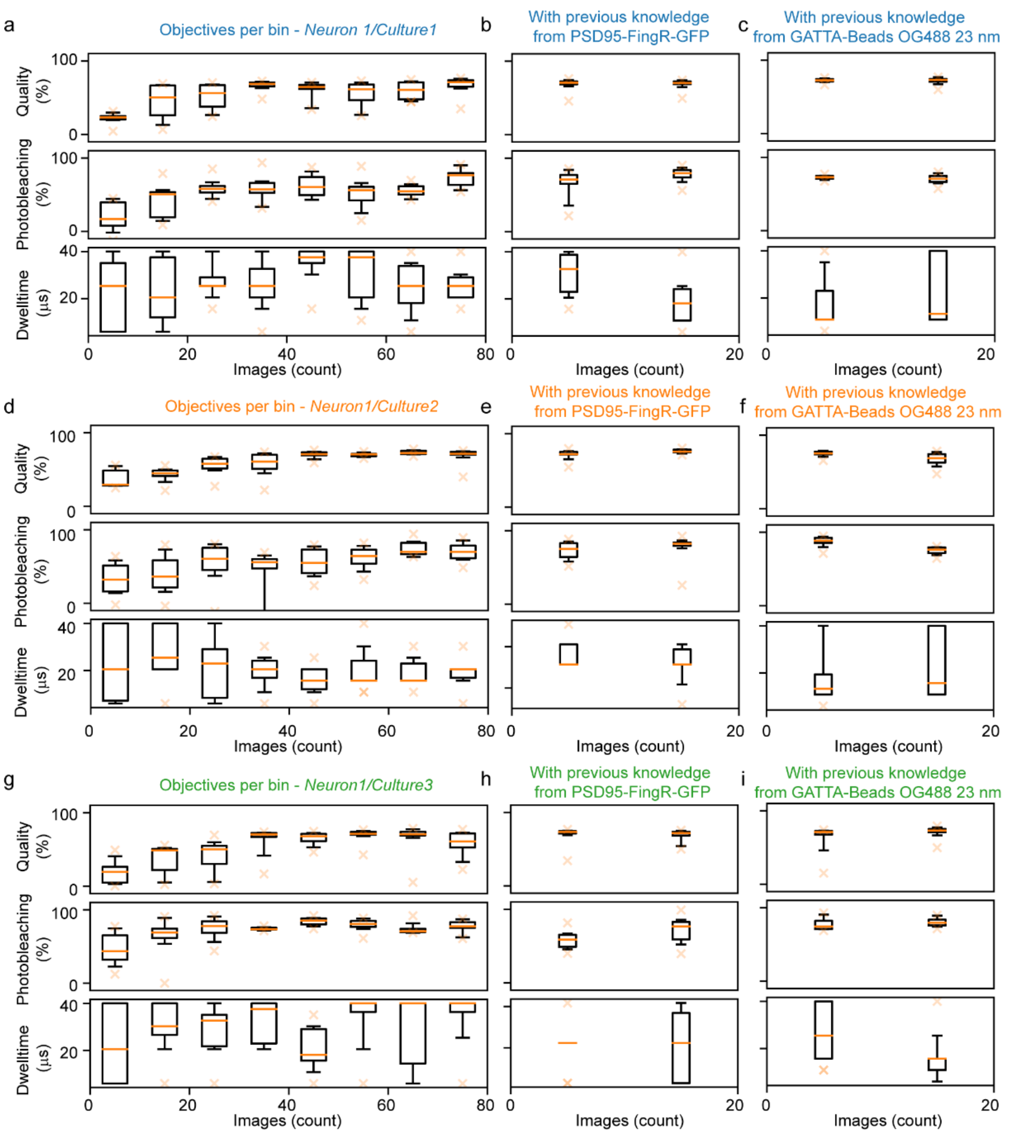

Supplementary Figure 22: Evolution of the objective values during Kernel TS optimization sequences on LifeAct-GFP in 3 living neurons with and without previous knowledge. Alternative representation of the data from Supplementary Fig. 21 d,e,f) to show the progression of all three objectives quantitatively. Each box corresponds to the bined objective values of 10 images. a,d,g) Complete sequences of 80 images without previous knowledge. b,e,h) Results obtained for the 20 images that were taken using previous knowledge from the reference sample PSD95-FingR-GFP. c,f,i) Results obtained for the 20 images that were taken using previous knowledge from the reference sample GATTAQUANT Nanobeads OG 488.

Figure interpretation: With this representation, we can evaluate all three objectives simultaneously. For two out of three neurons tested, at least one objective was less successful when using prior knowledge, compared to the last 20 images of the full optimization sequence. For *Neuron1/Culture3*, using previous knowledge from PSD95-FingR-GFP was successful. For *Neuron1/Culture2*, with both reference samples, the results from the full optimization sequence without prior knowledge were comparable to the sequences with prior knowledge. For *Neuron1/Culture1*, the photobleaching was slightly increased when using the prior knowledge from reference samples. Orange line indicates the median, box covers the 1st to the 3rd quartiles, and whiskers extend from 10th to 90th percentiles.

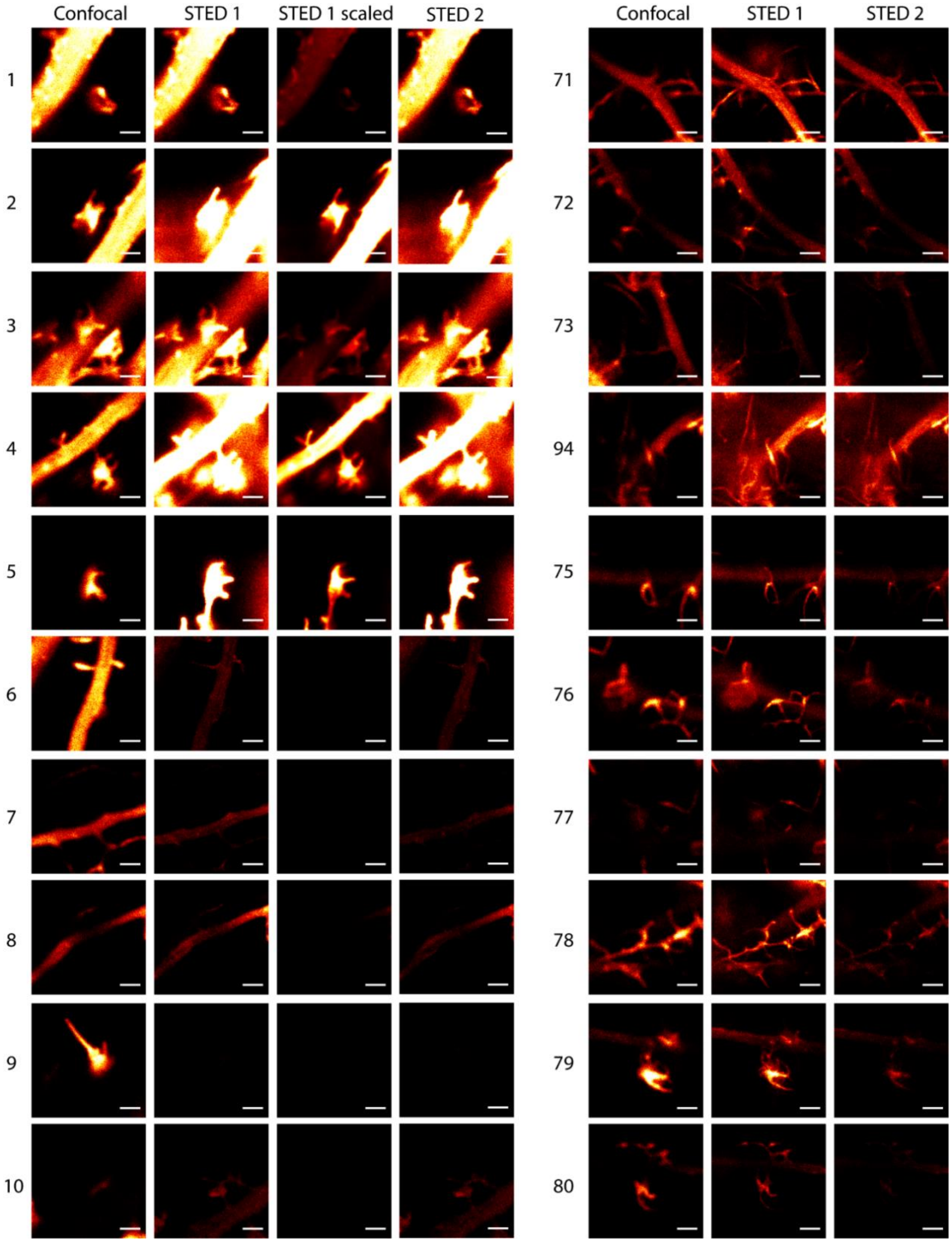

Supplementary Figure 23: Example images obtained during a Kernel TS optimization sequence on LifeAct-GFP on living neurons. Note the improvement in image quality (higher resolution) and photobleaching between images 1 to 10 and 71 to 80 during the Kernel TS optimization sequence. Two high quality images of the LifeAct dendritic spines were obtained for 70% of the last 10 images of the sequence (compared to none of the first 10 images). Scale bars 1 µm. The intensity scale was kept constant for all presented STED images.

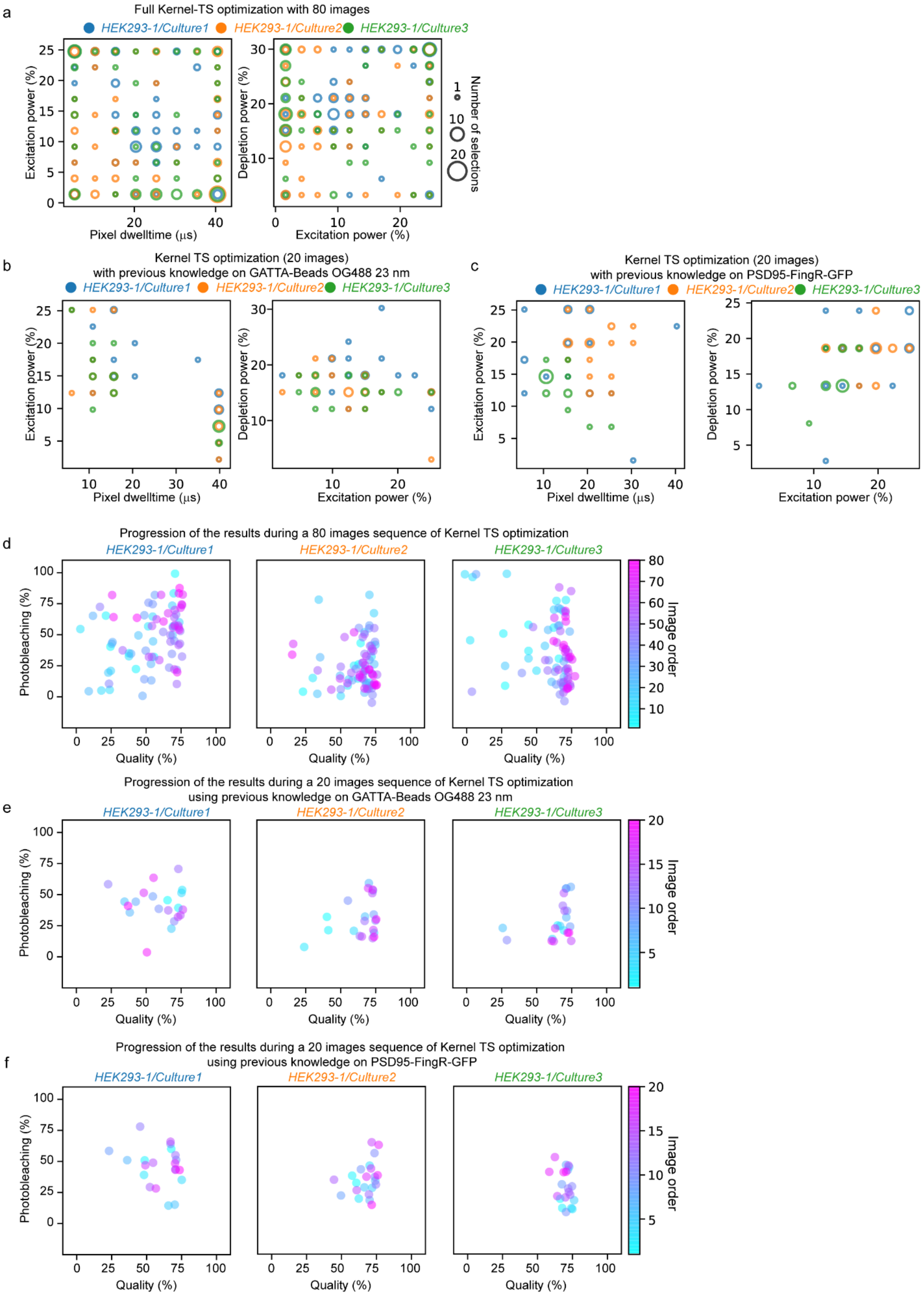
a
Full Kernel-TS optimization with 80 images
HEK293-1/Culture1
HEK293-1/Culture2
HEK293-1/Culture3
Excitation power (%)
Pixel dwelltime (µs)
Depletion power (%)
Excitation power (%)
Number of selections
1
10
20
b
Kernel TS optimization (20 images)
with previous knowledge on GATTA-Beads OG488 23 nm
c
Kernel TS optimization (20 images)
with previous knowledge on PSD95-FingR-GFP
d
Progression of the results during a 80 images sequence of Kernel TS optimization
Photobleaching (%)
Quality (%)
Image order
e
Progression of the results during a 20 images sequence of Kernel TS optimization
using previous knowledge on GATTA-Beads OG488 23 nm
f
Progression of the results during a 20 images sequence of Kernel TS optimization
using previous knowledge on PSD95-FingR-GFP

Supplementary Figure 24: Kernel TS optimization on LifeAct-GFP in 3 samples of HEK293 cells for three independent samples with and without previous knowledge. Three objective (image quality, photobleaching, imaging speed) and three parameter (excitation laser power, depletion laser power and pixel dwell time) Kernel TS optimization was performed on LifeAct-GFP in living HEK293 cells. The results obtained for three different samples from three separate cultures are compared. a) Imaging parameter combinations during a full Kernel TS optimization trial (80 images, living HEK293 cells) without previous knowledge. b,c) Imaging parameter combinations selected for 20 images that were acquired using the previous knowledge obtained with the reference samples GATTAQUANT Nanobeads OG 488 (b) or PSD95-FingR-GFP in living neurons (c) (see Supplementary Figure 15). d) Evolution of the quality and photobleaching objectives for the 80 images taken during Kernel TS optimization, without previous knowledge (the parameters used to obtain those results are shown in panel a). e,f) Evolution of the quality and photobleaching objectives for 20 images taken during Kernel TS optimization, using the previous knowledge obtained during Kernel TS optimization on the reference samples GATTAQUANT Nanobeads OG 488 (e) or PSD95-FingR-GFP in living neurons (f) (Supplementary Figure 15). See Methods for a detailed description of the experimental design and procedures.

Figure interpretation: Panel a) shows that the required pixel dwell time, excitation power and depletion power that led to good quality images combined to reduced photobleaching varied considerably from one neuron to another. For *HEK293-1/Culture2* (orange), low excitation power (1 % - 0.4 µW) with long pixel dwell time (40 µs) resulted in the best trade-offs (image quality, photobleaching and imaging speed). For *HEK293-1/Culture1* (blue) moderate excitation power (8-15 % - 4.4-8.0 µW) and moderate pixel dwell time (20-30 µs) resulted in the best trade-offs. Finally, for *HEK293-1/Culture3* longer pixel dwell time (25-40 µs) and low excitation power (1 % - 0.4 µW) resulted in the best trade-offs. A good trade-off between image quality and photobleaching was reached for *HEK293-1/Culture2* and *HEK293-1/Culture3* toward the last 20 images (pink circles in panel d). In the case of *HEK293-1/Culture1* increased image quality led to increased photobleaching towards the end of the optimization sequence. The image quality was around 75 % for all samples while the photobleaching was below 50 % for *HEK293-1/Culture2* and *HEK293-1/Culture3* and between 50 % and 75 % for *HEK293-1/Culture1* in most of these last 20 images. The reduced photobleaching enabled two consecutive usable STED images of LifeAct-GFP in living HEK293 cells (e.g. last 5 images of one trial showed in Supplementary Fig. 28). When using the nanobeads as reference, the image quality was reduced for *HEK293-1/Culture1* and *HEK293-1/Culture2* but the photobleaching was comparable to the full optimization run without previous knowledge for all trials (panel e). The parameters that were determined to be optimal for the nanobeads (fast pixel dwell time, high excitation power, and moderate depletion power) were not suitable for all observed HEK293 cells tagged with LifeAct-GFP. Using the previous knowledge acquired with PSD95-FingR-GFP led to better results (panel f) compared to the nanobeads, but for *HEK293-1/Culture1* the image quality was lower and for *HEK293-1/Culture2* the photobleaching was higher than in the last 20 images of the full optimization run.

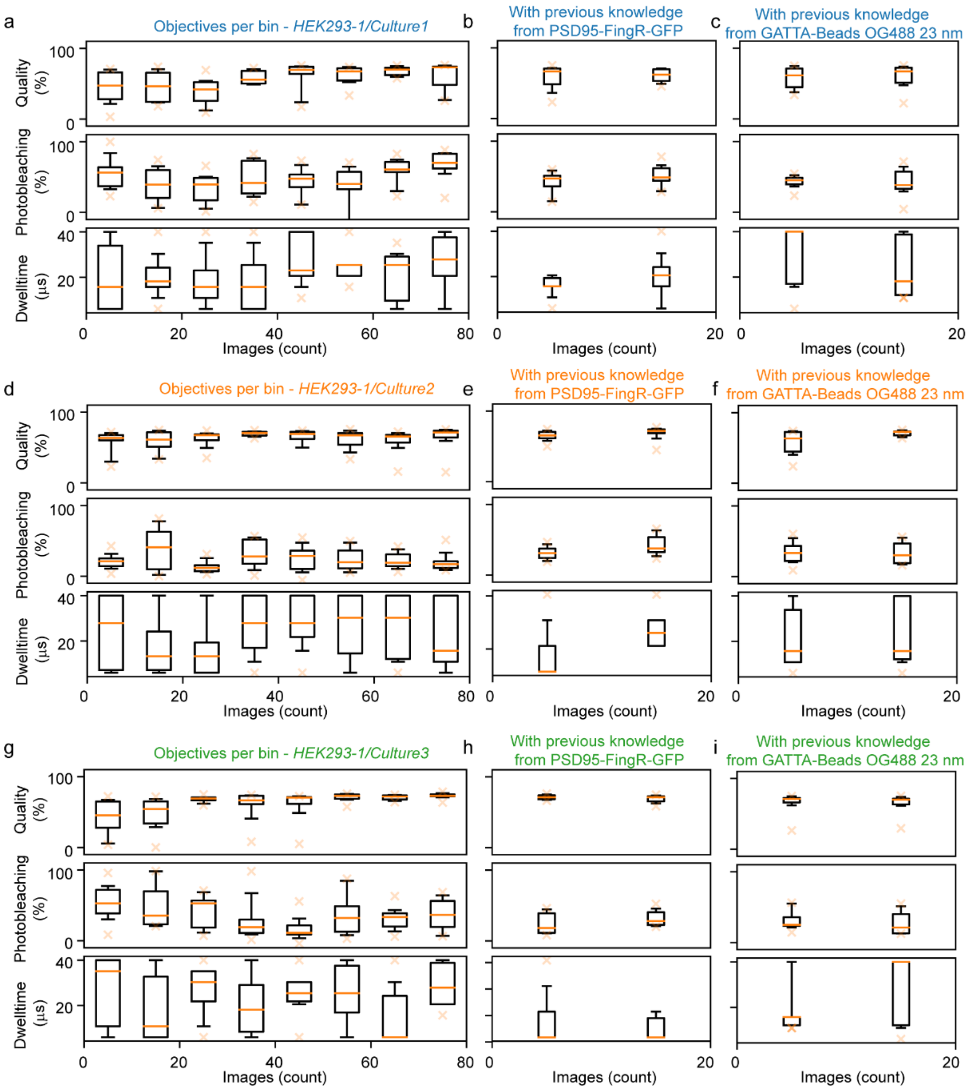

Supplementary Figure 25: Evolution of the objective values during Kernel TS optimization sequences on LifeAct-GFP in 3 samples of HEK293 cells with and without previous knowledge. Alternative representation of the data from fig. A to show the progression of all three objectives quantitatively. Each box corresponds to the bined objective values of 10 images. a,d,g) Complete sequences of 80 images without previous knowledge. b,e,h) Results obtained for the 20 images that were taken using previous knowledge from the reference sample PSD95-FingR-GFP. c,f,i) Results obtained for the 20 images that were taken using previous knowledge from the reference sample GATTAQUANT Nanobeads OG 488.

Figure interpretation: With this representation we can evaluate all three objectives simultaneously. Depending on the sample, good results could be obtained when using the previous knowledge obtained from PSD95-FingR-GFP for *HEK293-1/Culture3*, while for *HEK293-1/Culture2* the photobleaching was higher and for *HEK293-1/Culture1* the quality was slightly reduced when using previous knowledge. When using prior knowledge from the nanobeads, lower image quality was obtained for *HEK293-1/Culture1* and *HEK293-1/Culture2*. These results emphasize the variability in the results that can be obtained when using prior knowledge from reference samples for the presented optimization task. Orange line indicates the median, box covers the 1st to the 3rd quartiles, and whiskers extend from 10th to 90th percentiles.

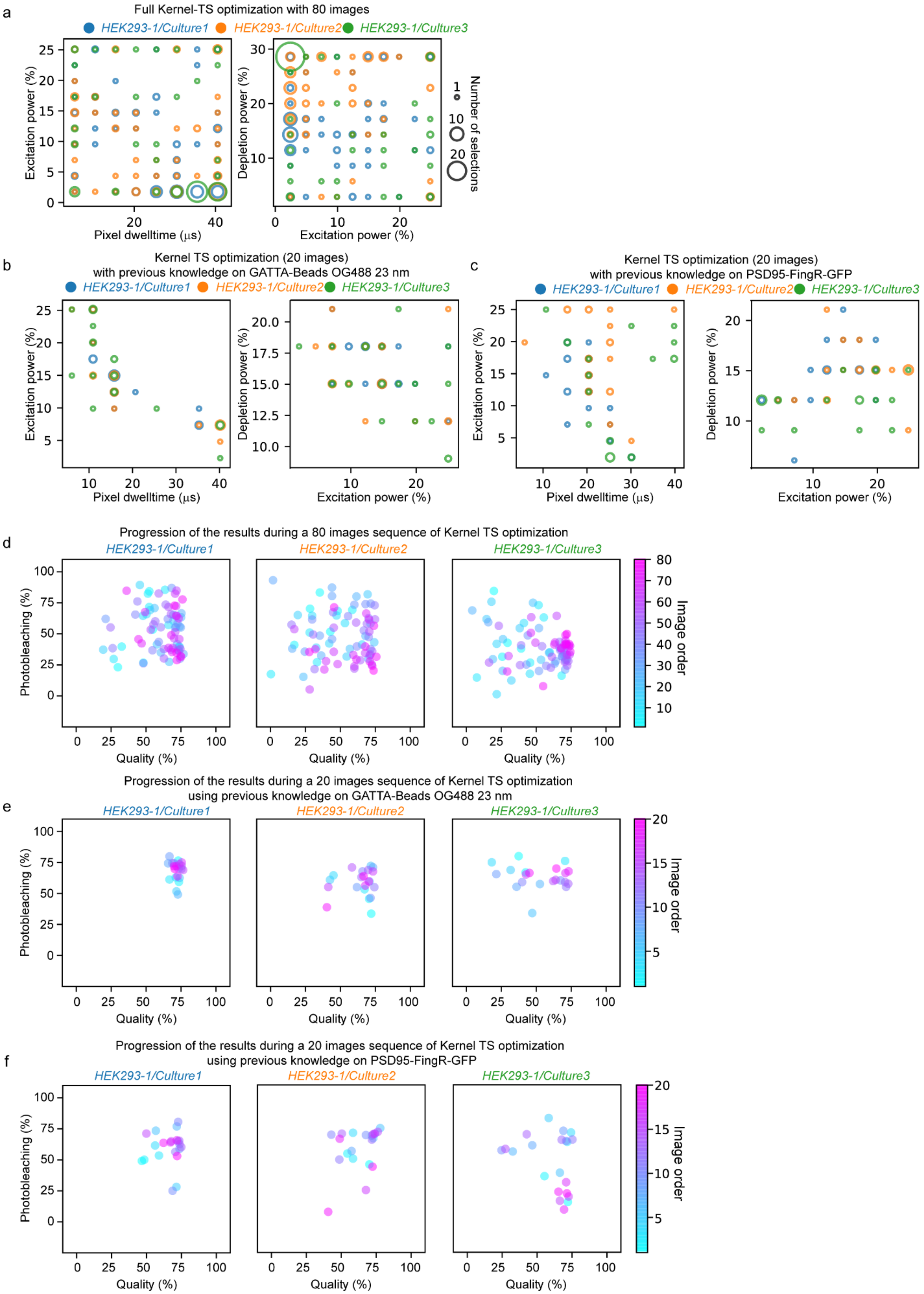

a
Full Kernel-TS optimization with 80 images
HEK293-1/Culture1
HEK293-1/Culture2
HEK293-1/Culture3
Excitation power (%)
Pixel dwelltime (µs)
Depletion power (%)
Excitation power (%)
Number of selections
b
Kernel TS optimization (20 images)
with previous knowledge on GATTA-Beads OG488 23 nm
c
Kernel TS optimization (20 images)
with previous knowledge on PSD95-FingR-GFP
d
Progression of the results during a 80 images sequence of Kernel TS optimization
Photobleaching (%)
Quality (%)
Image order
e
Progression of the results during a 20 images sequence of Kernel TS optimization
using previous knowledge on GATTA-Beads OG488 23 nm
f
Progression of the results during a 20 images sequence of Kernel TS optimization
using previous knowledge on PSD95-FingR-GFP

Supplementary Figure 26: Kernel TS optimization on Membrane-GFP for three independent samples of HEK293 cells with and without previous knowledge. Three objective (image quality, photobleaching, imaging speed) and three parameter (excitation laser power, depletion laser power and pixel dwell time) Kernel TS optimization was performed on Membrane-GFP in living HEK293 cells. The results obtained for three different samples from three separate cultures are compared. a) Imaging parameter combinations during a full Kernel TS optimization trial (80 images, living HEK293) without previous knowledge. b,c) Imaging parameter combinations selected for 20 images that were acquired using the previous knowledge obtained with the reference samples GATTAQUANT Nanobeads OG 488 (b) or PSD95-FingR-GFP in living neurons (c) (see Supplementary Figure 15). d) Evolution of the quality and photobleaching objectives for the 80 images taken during Kernel TS optimization, without previous knowledge (the parameters used to obtain those results are shown in panel a). e,f) Evolution of the quality and photobleaching objectives for 20 images taken during Kernel TS optimization, using the previous knowledge obtained during Kernel TS optimization on the reference samples GATTAQUANT Nanobeads OG 488 (e) or PSD95-FingR-GFP in living neurons (f) (Supplementary Figure 15). See Methods for a detailed description of the experimental design and procedures.

Figure interpretation: Panel a) shows that, preferably, long pixel dwell time (higher than 20 µs) and low excitation power (1%) led to good quality images combined to reduced photobleaching, but that the required depletion power that could be used to fulfill the objectives varies between samples. High depletion power (30 % - 99 mW) for *HEK293-1/Culture3* (green), moderate depletion power (around 25 % - 82 mW) for *HEK293-1/Culture2* (orange) and slightly lower depletion power (around 15 % - 50 mW) for *HEK293-1/Culture1* (blue) resulted in the best trade-offs (image quality, photobleaching and imaging speed). A good trade-off between image quality and photobleaching was reached for the three neurons toward the last 20 images (pink circles in panel d) but biological variability between regions led to some failure in all three samples (even towards the end of the optimization sequence). The image quality was around 75 % while the photobleaching was below 50 % for most of these last 20 images. The reduced photobleaching enabled two consecutive usable STED images of Membrane-GFP in living HEK293 cells (e.g. last 5 images of one trial showed in Supplementary Fig.8). When using the nanobeads as reference, the image quality is high only for *HEK293-1/Culture1* and the photobleaching was higher for all trials. The parameters that were determined to be optimal for the nanobeads (fast pixel dwell time, high excitation power, and moderate depletion power) were not suitable for the observed Membrane-GFP in living HEK293 cells. Using the previous knowledge acquired with PSD95-FingR-GFP led to better results concerning the photobleaching (panel f) compared to the nanobeads, but for the majority of the last 20 images better results for both the image quality and the photobleaching were obtained during the full optimization run without previous knowledge.

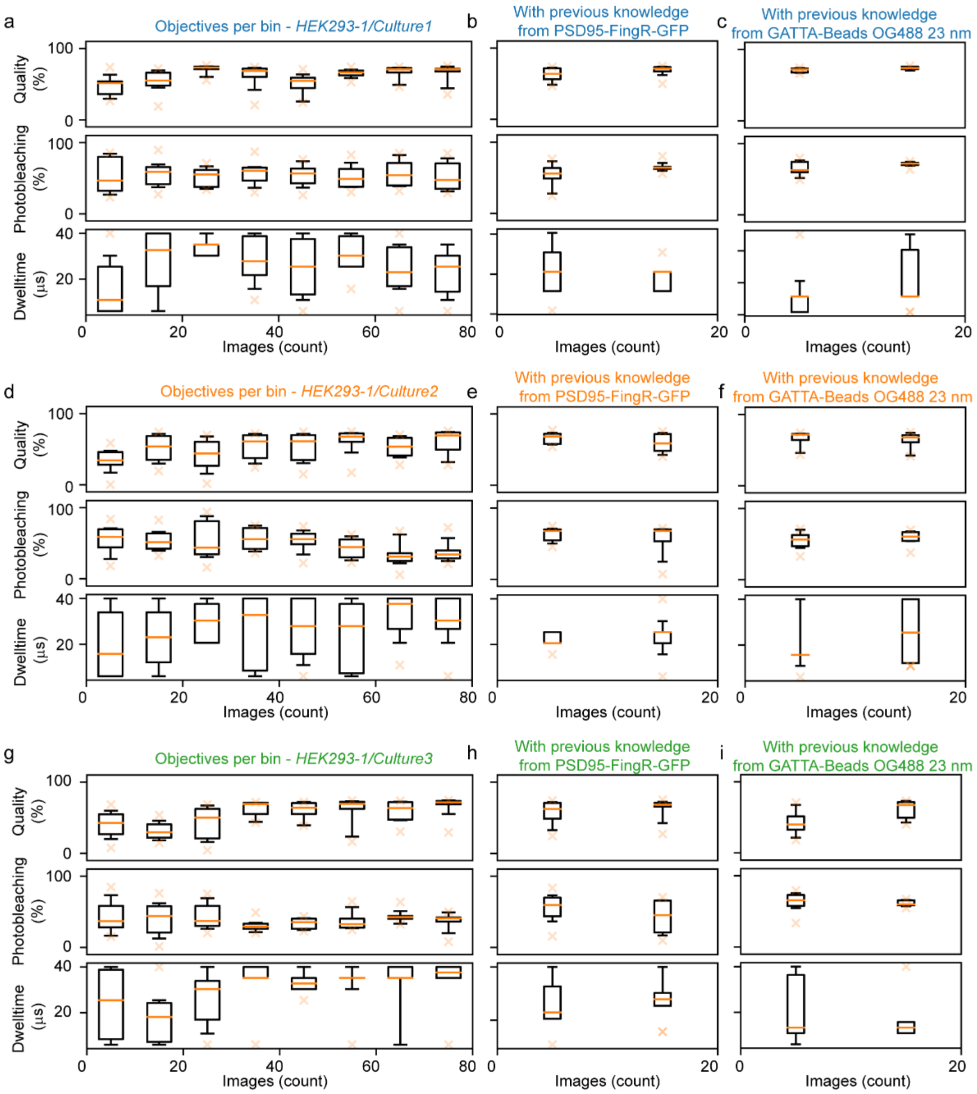

Supplementary Figure 27: Evolution of the objective values during Kernel TS optimization sequences on Membrane-GFP in 3 different samples of HEK293 cells with and without previous knowledge. Alternative representation of the data from Supplementary Fig. 26 d,e,f) to show the progression of all three objectives quantitatively. Each box corresponds to the bined objective values of 10 images. a,d,g) Complete sequences of 80 images without previous knowledge. b,e,h) Results obtained for the 20 images that were taken using previous knowledge from the reference sample PSD95-FingR-GFP. c,f,i) Results obtained for the 20 images that were taken using previous knowledge from the reference sample GATTAQUANT Nanobeads OG 488.

Figure interpretation: With this representation we can evaluate all three objectives simultaneously. For each sample tested, at least one objective was less successful when using prior knowledge, compared to the last 20 images of the full optimization sequence without previous knowledge. For Membrane-GFP in HEK293 cells more specifically, we observe that the photobleaching was optimized successfully below 50 % for the majority of the last 20 images of the full optimization run without previous knowledge, while using previous knowledge from the references sample led to higher photobleaching values. These results emphasize the limitations of using prior knowledge from reference samples for the presented optimization task. Orange line indicates the median, box covers the 1st to the 3rd quartiles, and whiskers extend from 10th to 90th percentiles.

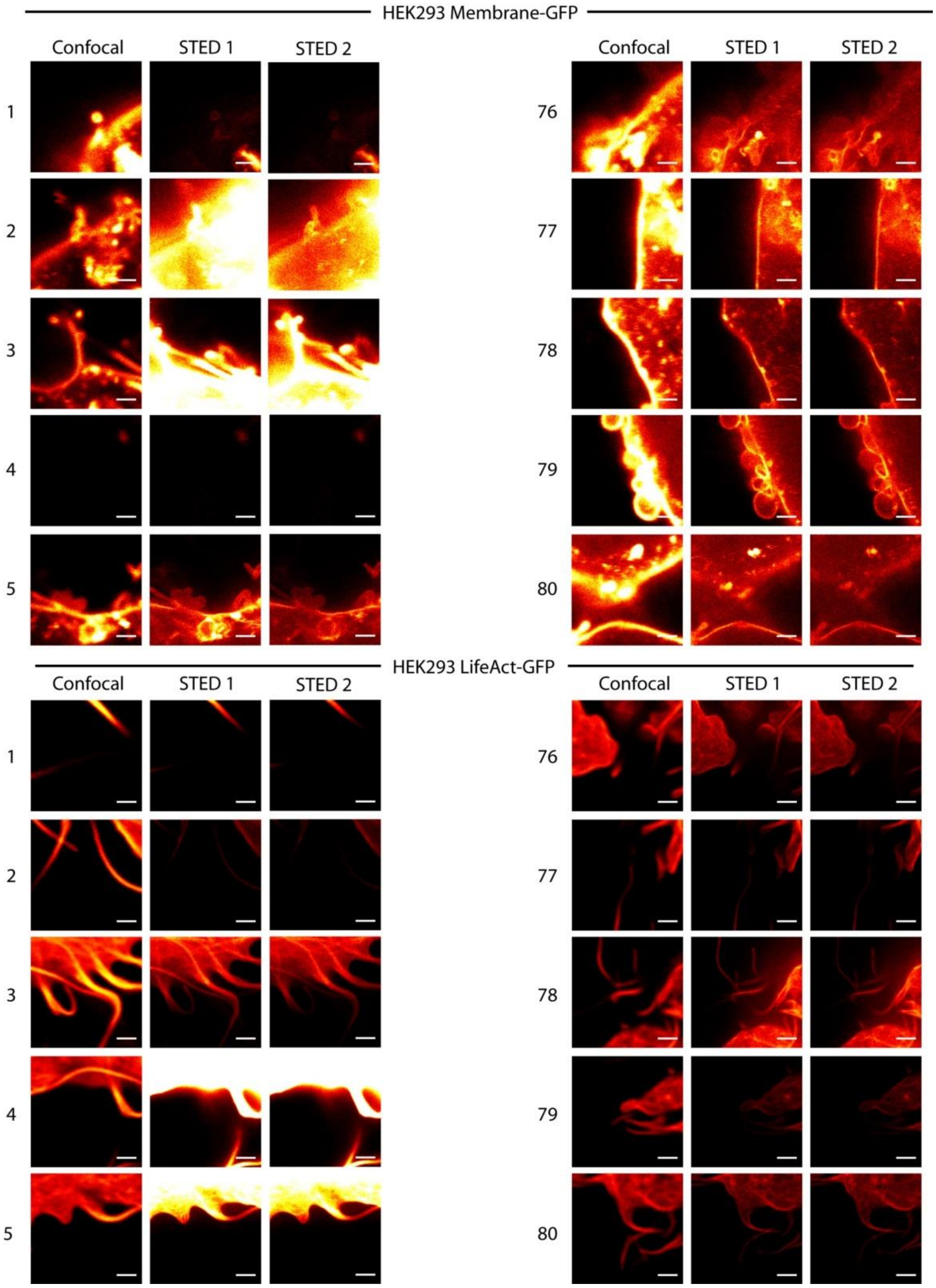

Supplementary Figure 28: Example of images obtained during optimization sequences on Membrane-GFP (top) and LifeAct-GFP (botton) expressed in HEK293 cells. Note the improvement in image quality (resolution) between images 1 to 5 and 76 to 80 during the Kernel TS optimization sequence. Two high quality images of the cell membrane (top) and of LifeAct in HEK cells were obtained for all of the last 5 images of the sequence. In most of the first 5 images of each sequence, low resolution or very low signal to noise ratio was obtained. Scale bars 1 µm. The intensity scale was kept constant for all presented STED images.

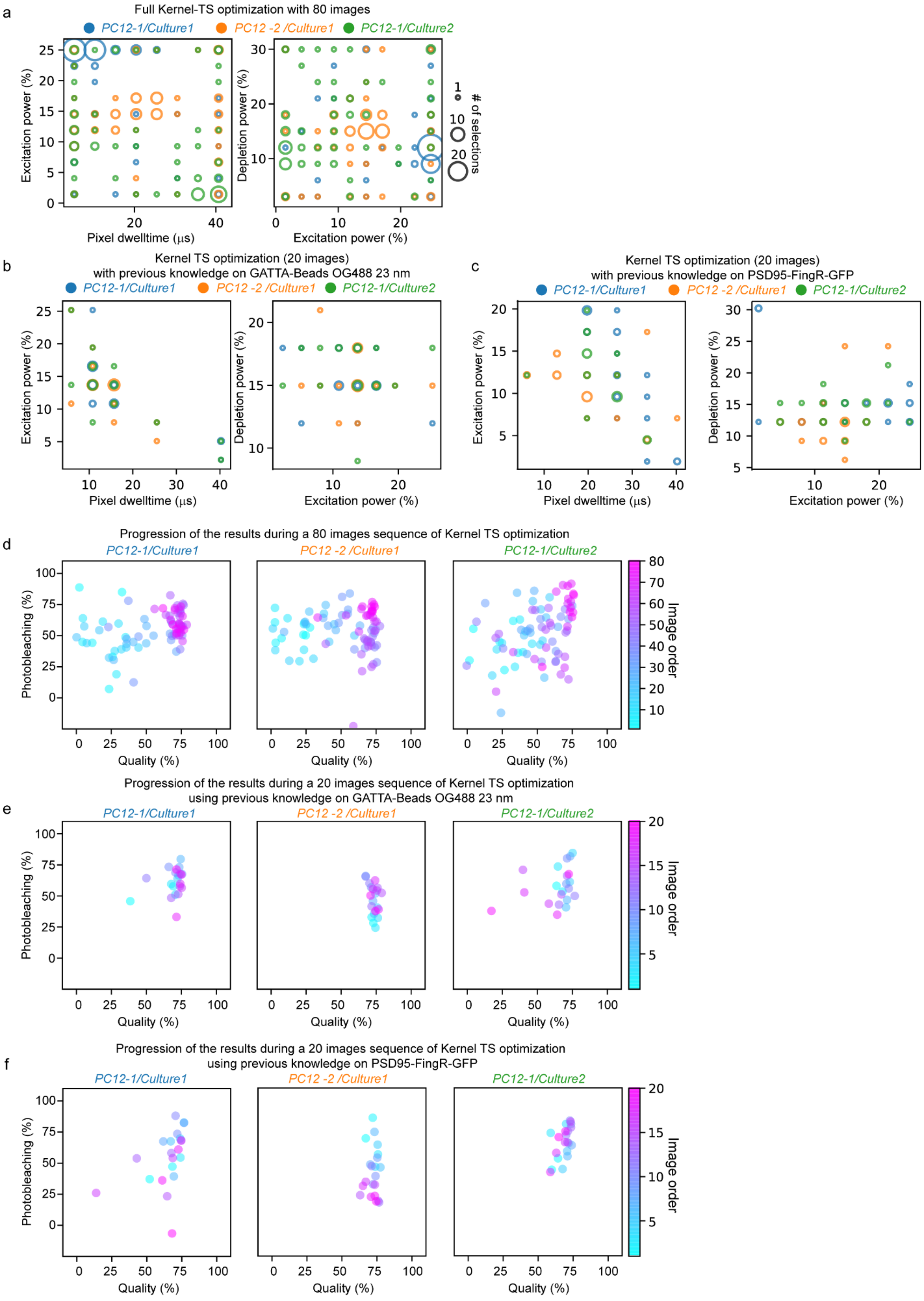
a
Full Kernel-TS optimization with 80 images
PC12-1/Culture1
PC12 -2 /Culture1
PC12-1/Culture2
Excitation power (%)
Pixel dwelltime (μs)
Depletion power (%)
Excitation power (%)
# of selections
b
Kernel TS optimization (20 images)
with previous knowledge on GATTA-Beads OG488 23 nm
c
Kernel TS optimization (20 images)
with previous knowledge on PSD95-FingR-GFP
d
Progression of the results during a 80 images sequence of Kernel TS optimization
Photobleaching (%)
Quality (%)
Image order
e
Progression of the results during a 20 images sequence of Kernel TS optimization
using previous knowledge on GATTA-Beads OG488 23 nm
f
Progression of the results during a 20 images sequence of Kernel TS optimization
using previous knowledge on PSD95-FingR-GFP

Supplementary Figure 29: Kernel TS optimization on LifeAct-GFP for three independent samples of PC12 cells with and without previous knowledge. Three objective (image quality, photobleaching, imaging speed) and three parameter (excitation laser power, depletion laser power and pixel dwell time) Kernel TS optimization was performed on Life-Act-GFP in living PC12 cells. The results obtained for three different samples from two separate cultures are compared. a) Imaging parameter combinations during a full Kernel TS optimization trial (80 images, living PC12) without previous knowledge. b,c) Imaging parameter combinations selected for 20 images that were acquired using the previous knowledge obtained with the reference samples GATTAQUANT Nanobeads OG 488 (b) or PSD95-FingR-GFP in living neurons (c) (see Supplementary Figure 15). d) Evolution of the quality and photobleaching objectives for the 80 images taken during Kernel TS optimization, without previous knowledge (the parameters used to obtain those results are shown in panel a). e,f) Evolution of the quality and photobleaching objectives for 20 images taken during Kernel TS optimization, using the previous knowledge obtained during Kernel TS optimization on the reference samples GATTAQUANT Nanobeads OG 488 (e) or PSD95-FingR-GFP in living neurons (f) (Supplementary Figure 15). See Methods for a detailed description of the experimental design and procedures.

Figure interpretation: Panel a) shows that the required pixel dwell time and excitation power that led to good quality images combined to reduced photobleaching vary greatly from one cell to another. For *PC12-1/Culture2* (green), low excitation power (1 % - 0.4 µW) with long pixel dwell time (35-40 µs) resulted in the best trade-offs. For *PC12-2/Culture1* (orange) moderate excitation power (15-18 % - 8.0-9.4 µW) and moderate pixel dwell time (15-25 µs) resulted in the best trade-offs. Finally, for *PC12-1/Culture1* (blue) short pixel dwell time (5-10 µs) and high excitation power (25 % - 13 µW) resulted in the best trade-offs. A good trade-off between image quality and photobleaching was reached for *PC12-1/Culture1* and for *PC12-2/Culture1* toward the last 20 images (pink circles in panel d). In the case of *PC12-1/Culture2* as small amount of low quality images were acquired even toward the end of the sequence and a larger number of images would probably have been necessary to improve further optimize the imaging parameters. For most of the last 20 image, the quality score was around 75 % for all samples while the photobleaching was generally below 70 %. The reduced photobleaching enabled two consecutive usable STED images of LifeAct-GFP in living PC12 cells (e.g. last 5 images of one trial showed in Supplementary Fig. 31). In this case, optimization using prior knowledge from GATTAQUANT Nanobeads or PSD95-FingR-GFP optimization sequences was successful and led to comparable trade-offs to the full optimization sequence without prior knowledge.

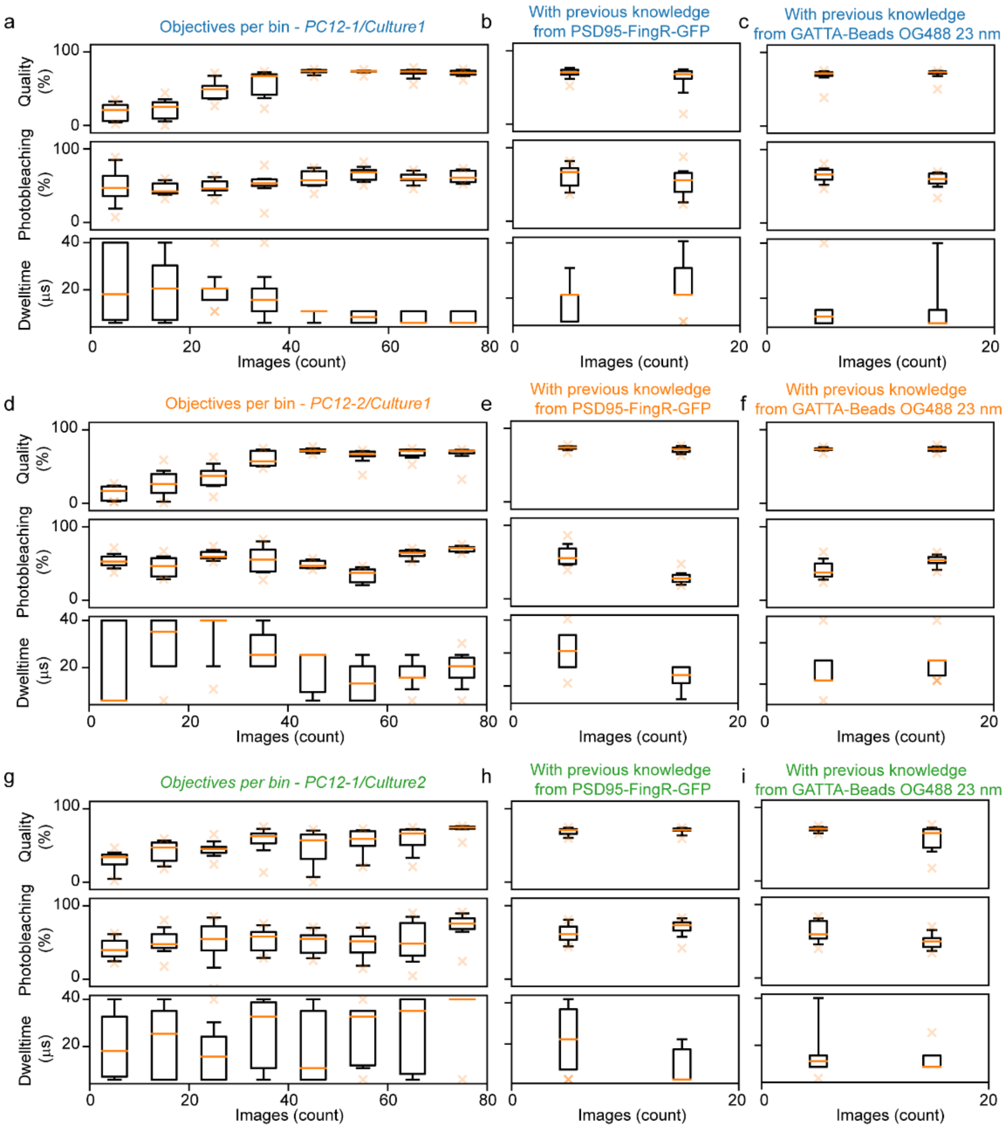

Supplementary Figure 30: Evolution of the objective values during Kernel TS optimization sequences on LifeAct-GFP in 3 different samples of PC12 cells with and without previous knowledge. Alternative representation of the data from fig. A to show the progression of all three objectives quantitatively. Each box corresponds to the bined objective values of 10 images. a,d,g) Complete sequences of 80 images without previous knowledge. b,e,h) Results obtained for the 20 images that were taken using previous knowledge from the reference sample PSD95-FingR-GFP. c,f,i) Results obtained for the 20 images that were taken using previous knowledge from the reference sample GATTAQUANT Nanobeads OG 488.

Figure interpretation: With this representation we can evaluate all three objectives simultaneously. For Life-Act-GFP in PC12 cells, we observe that the pixel dwell time was optimized successfully below 20 usec for the majority of the last 20 images off the full optimization run in *PC12-1/Culture1* and *PC12-2/Culture1*, while using the previous knowledge from the references sample led to slightly higher pixel dwell time values. In the case of LifeAct in PC12 cells, the use of prior knowledge from reference samples would have helped to speed up the optimization process. Orange line indicates the median, box covers the 1st to the 3rd quartiles, and whiskers extend from 10th to 90th percentiles.

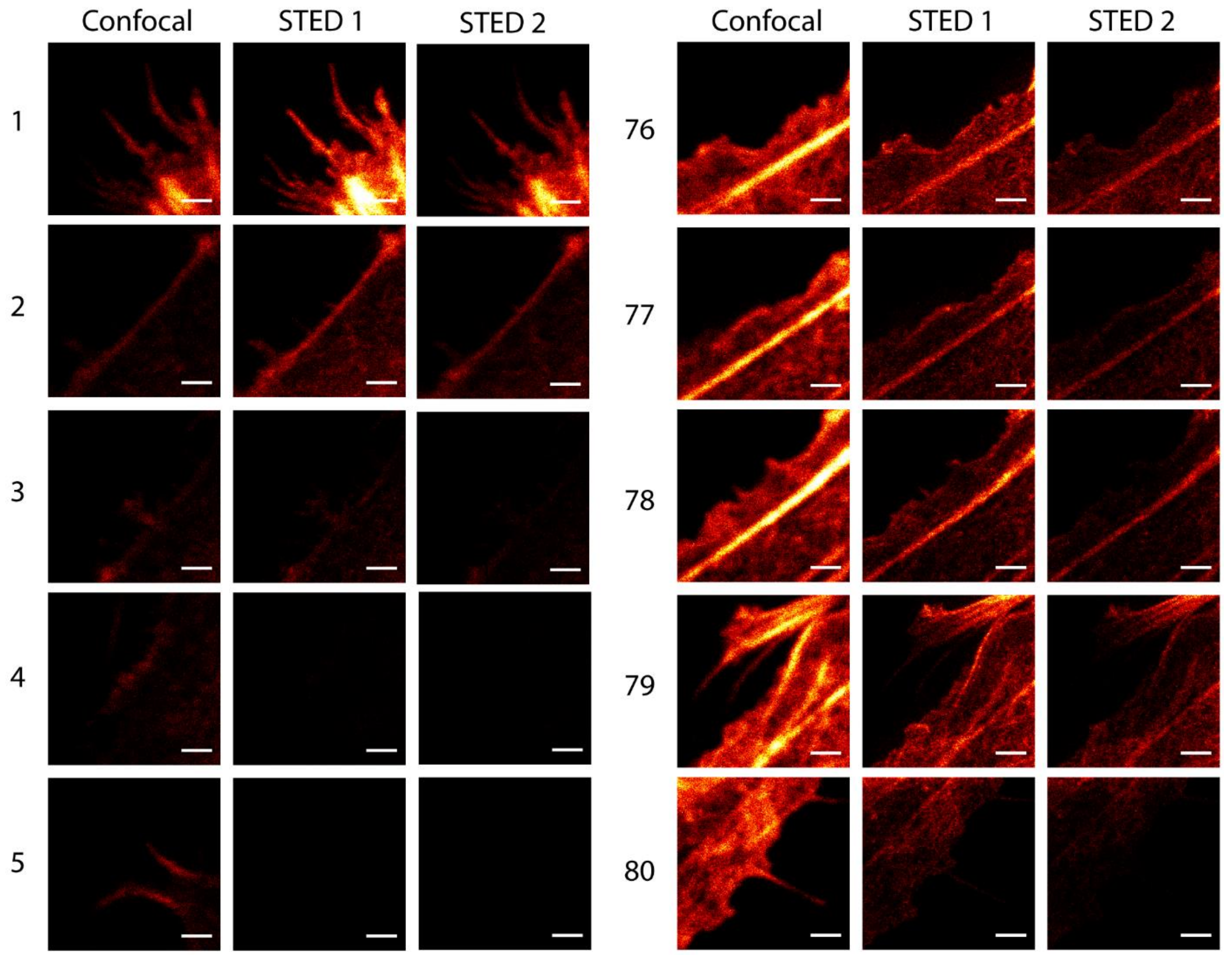

Supplementary Figure 31: Example of images obtained during optimization sequences, on LifeAct-GFP expressed in PC12 cells. Note the improvement in image quality (higher signal to noise ratio and higher resolution) between images 1 to 5 and 76 to 80 during the Kernel TS optimization sequence. Two high quality images of the cell membrane of LifeAct in PC12 cells were obtained for 4 of the last 5 series (but only 1 for the first 5 images). Scale bars 1 µm. The intensity scale was kept constant for all presented STED images of the same sequence.

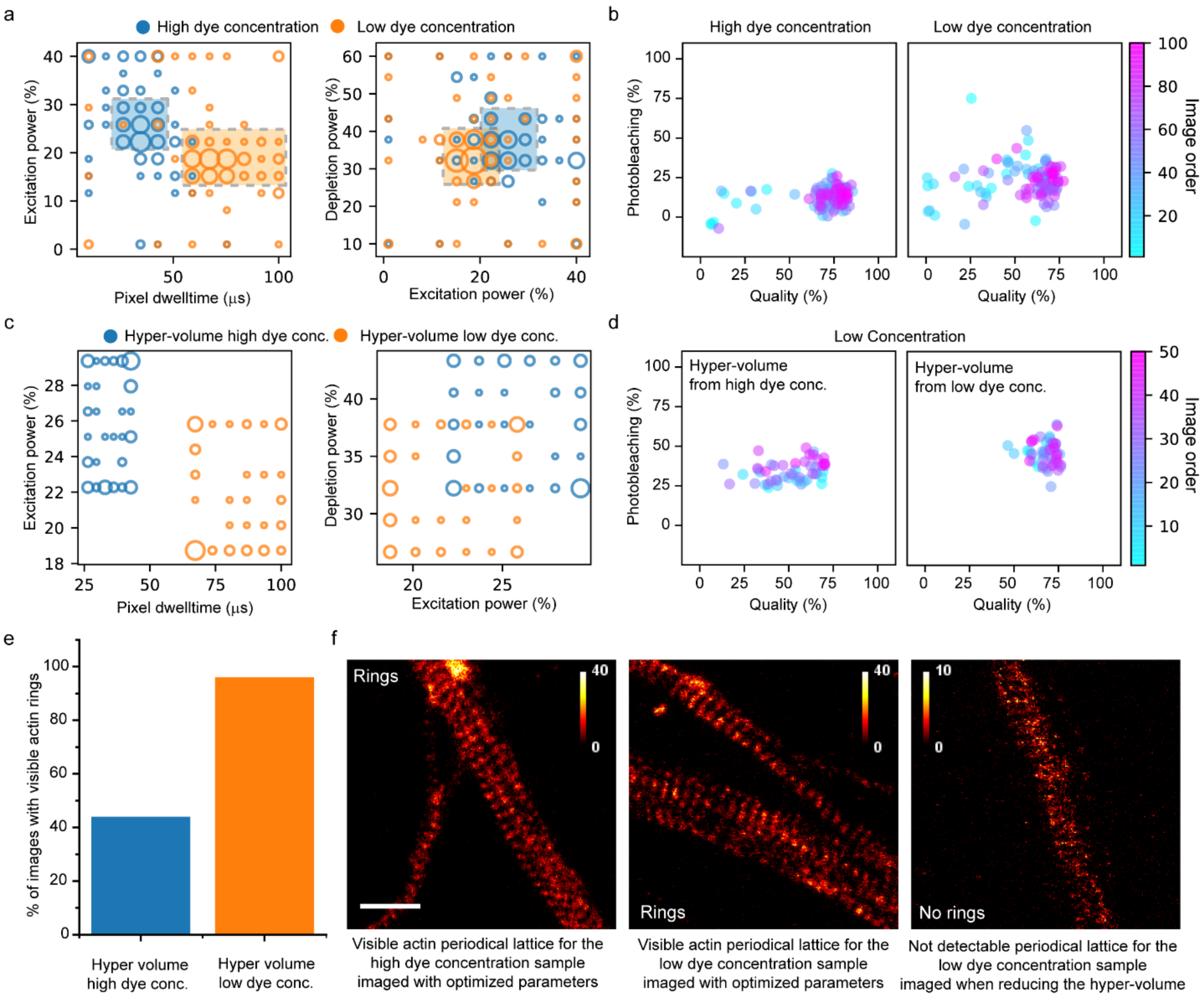

Supplementary Figure 32: Kernel TS optimization using previous knowledge with different staining concentration of Phalloidin-STAR635 on fixed neurons. Three objectives (image quality, photobleaching, imaging speed) and three parameters (excitation laser power, depletion laser power and pixel dwell time) Kernel TS optimization was performed on fixed neurons stained with different concentrations of phalloidin-STAR635. The previous knowledge acquired in those optimization sequences was used as reference to evaluate how this could accelerate the optimization process on another concentration. Images acquired at the beginning and at the end of both optimization sequences are shown in Supplementary Fig. 33. a) Parameter combinations that were sampled with Kernel TS optimization for high concentration (blue) and low concentration (orange). The size of the circles indicate how often a parameter combination was sampled. Shown are two planes of the three dimensional parameter space. b) Evolution of the quality and photobleaching objectives for the 100 images acquired during Kernel TS optimization. While short pixel dwell time (between 10 and 20µs) with high excitation power were possible given high concentration, longer pixel dwell time (between 60 and 100 µs) with low excitation power were necessary to obtain a good trade-off between image quality and bleach with low concentration, as shown in b). c) The reduced hyper volume of parameters was determined from the most sampled combinations that were obtained in the full optimization sequence (light blue and orange rectangles in panel a). The low dye concentration sample was imaged using the reduced hyper-volume from its optimization sequence (orange circles) and from the most sampled combination from the high dye concentration optimization sequence (blue circles). A new optimization sequence on 50 images was performed for both reduced hyper-volumes. d) The reduced hyper-volume from the high concentration

sample led to a small number of high quality images with low photobleaching (19 out of 50 images with Quality >~60 % and Photobleaching <~60 %) (left). In contrast when using the reduced hyper-volume resulting from optimization on the same sample, the trade-off between objectives resulted in 39 out of 50 images of high quality and low photobleaching (right). e) The images that were taken in the optimization sequences with reduced hyper-volumes of parameters were evaluated for the presence of clearly detectable actin rings (see example images in panel f). Rings were detected in only 22 out of 50 images when using the reduced hyper-volume of parameters from the same samples, while for the hyper-volume from the same samples, 44 out of 50 images showed clearly distinguishable actin rings. Scale bar 1µm.

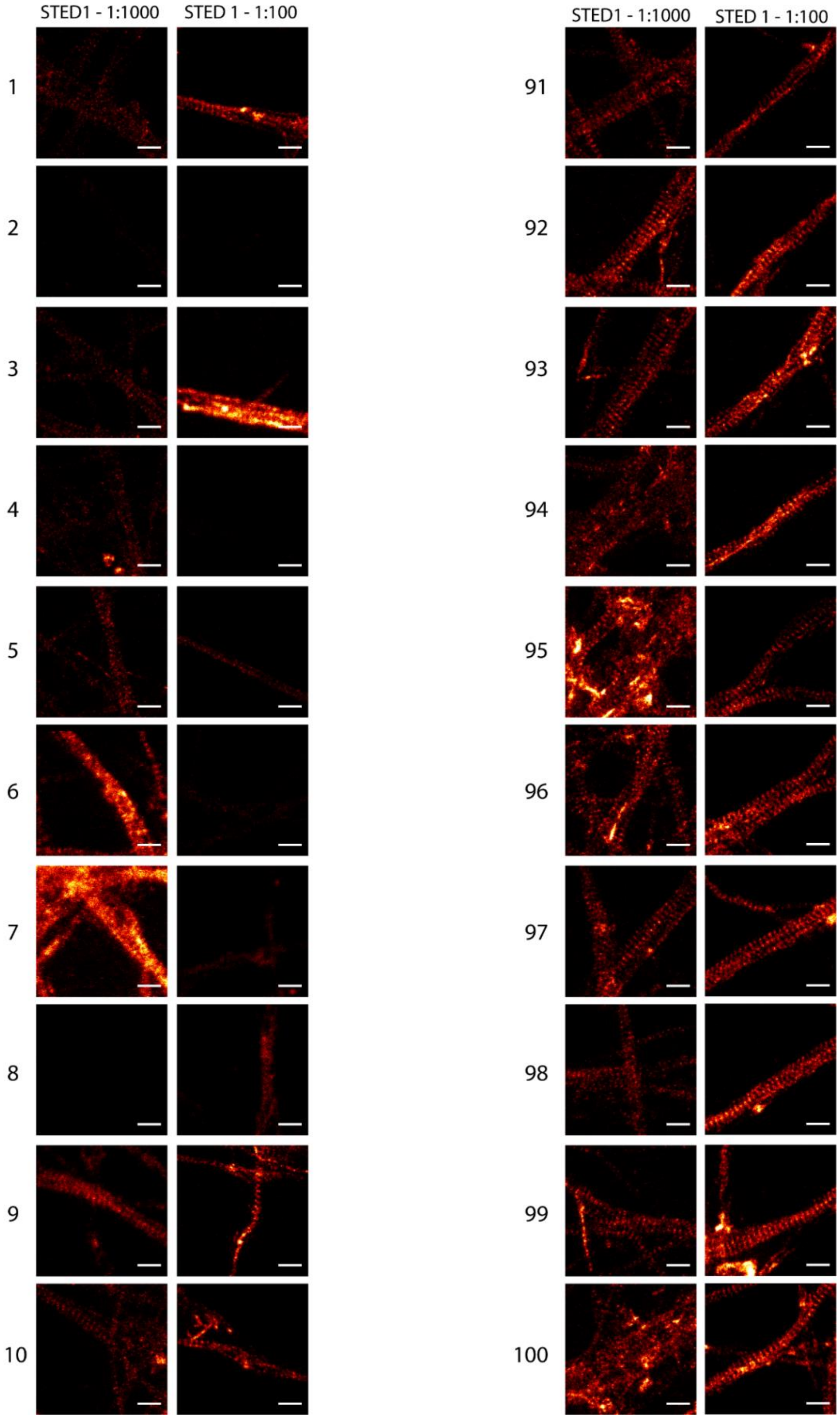

Supplementary Figure 33: Example images of fixed neurons stained with two different concentration of Phalloidin-STAR635 (1:100 - high dye concentration, 1:1000 - low dye concentration). Note the improvement in image quality (higher signal to noise ratio and higher resolution) between images 1 to 10 and 91 to 100 during both Kernel TS optimization sequences with high and low phalloidin-STAR635 staining concentration. For both staining concentrations, the periodical actin lattice was visible in all images from 91 to 100. Scale bars 1 µm. The intensity scale was kept constant for all presented STED images of the same sequence.

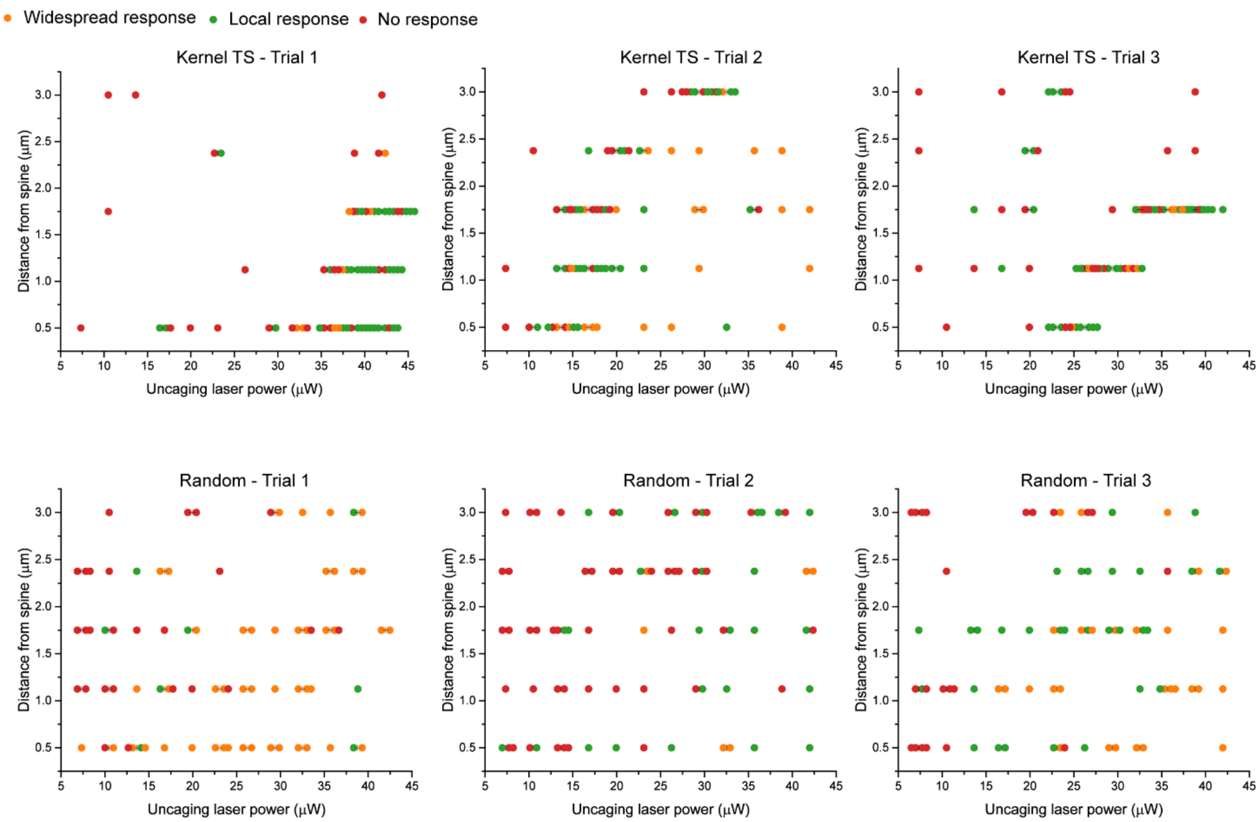

Supplementary Figure 34: Examples of three different trials using Kernel TS optimization or random parameter sampling to obtain localized Ca^{2+} response after glutamate uncaging. This shows the high variability that is observed between samples in the evoked responses. Green dots show measured local Ca^{2+} responses that are desired for the optimization of the photostimulation of single dendritic spines. The minimal distance between uncaging laser spot and spine head, as well as the required laser power to obtain a localized response vary greatly between cells.

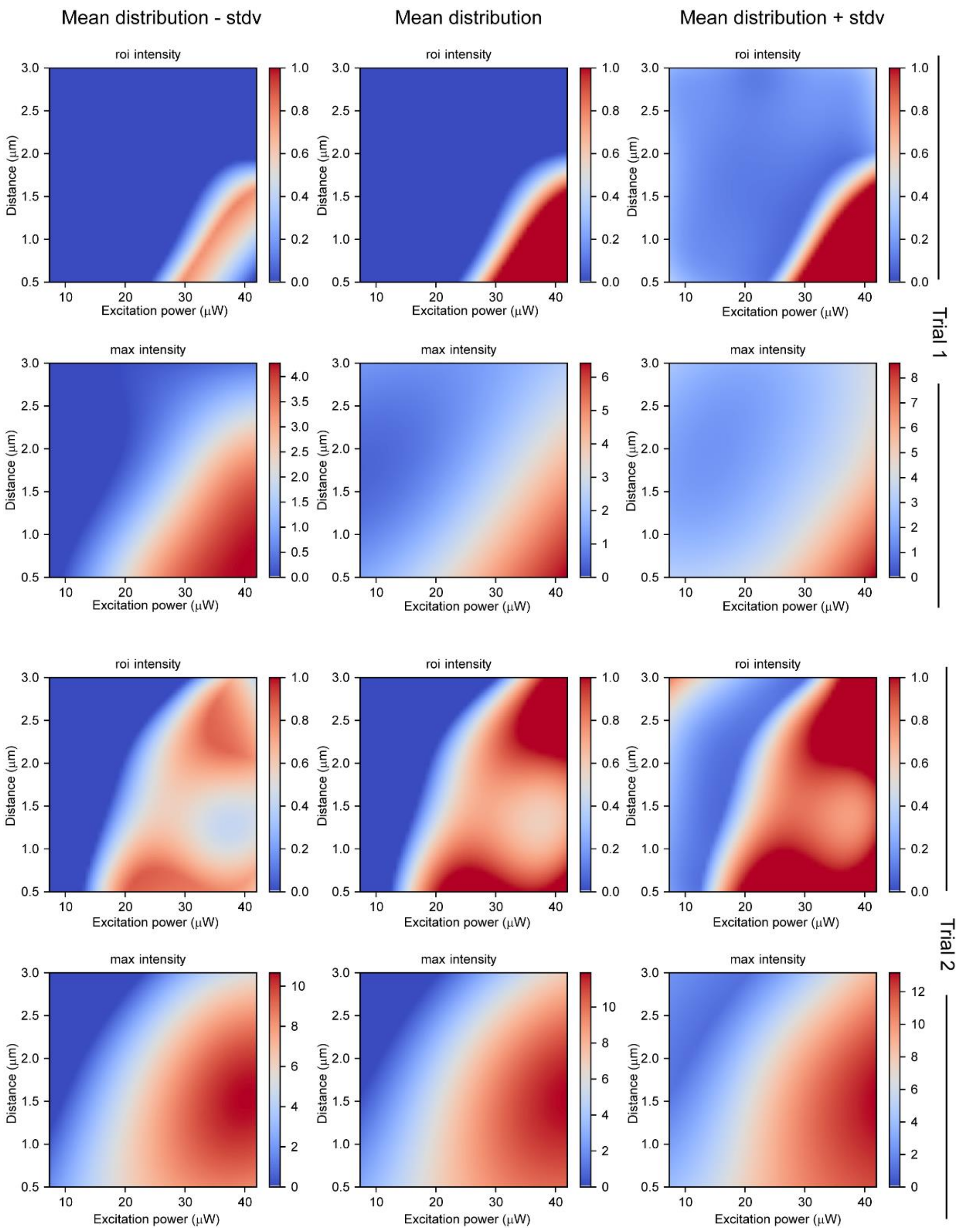

Supplementary Figure 35: Posterior prediction given by kernel regression for both objectives at the end of two different optimization trials of glutamate uncaging. For each trial, the first row shows the expected ROI intensity (middle) within one standard deviation (left and right), and the second row shows the expected max intensity (middle) within one standard deviation (left and right).

Figure interpretation: In the first trial, the highest predicted ROI intensity and max intensity are obtained with a low distance and high excitation power. In the second trial, the highest ROI is rather predicted in regions characterized either by high excitation power and high distance, or medium excitation power and low distance. The amplitude of the highest predicted max intensity is also much higher in the second trial (~10) compared with the first trial (~6). This illustrates the high variability between parameters appearing as relevant depending on the samples.

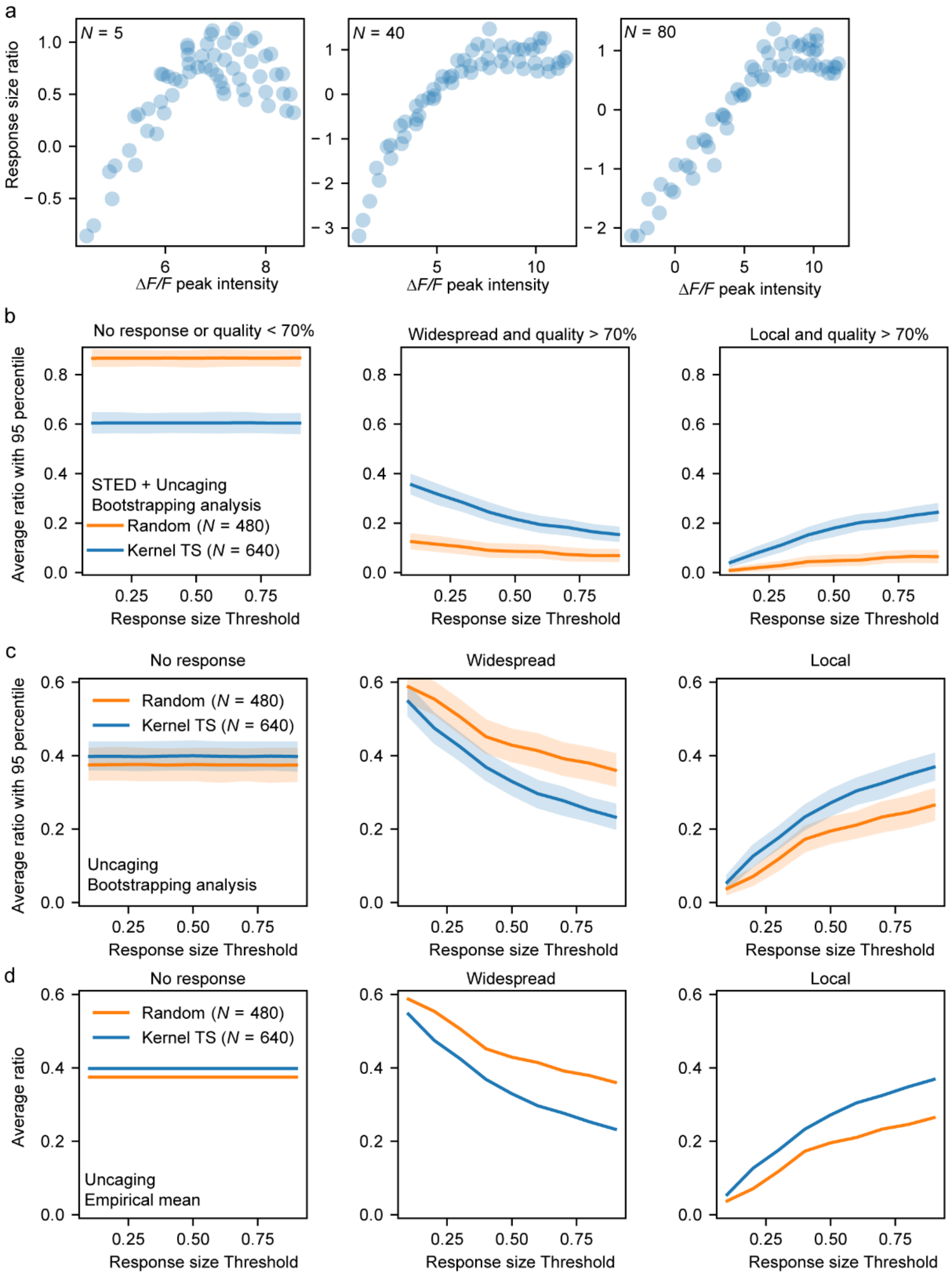

Supplementary Figure 36: Estimate of the objective space and observed ratio of response per threshold during Kernel TS optimization and random parameter sampling of glutamate uncaging. a) Expected options given the kernel regression predictions after N observations. b) Mean ratio of response per threshold with 95% confidence interval obtained by bootstrapping analysis considering glutamate uncaging and STED optimization. c) Mean ratio of response per threshold with 95% confidence interval obtained by bootstrapping analysis considering glutamate uncaging only. d) Empirical mean ratio of response per threshold considering glutamate uncaging only.

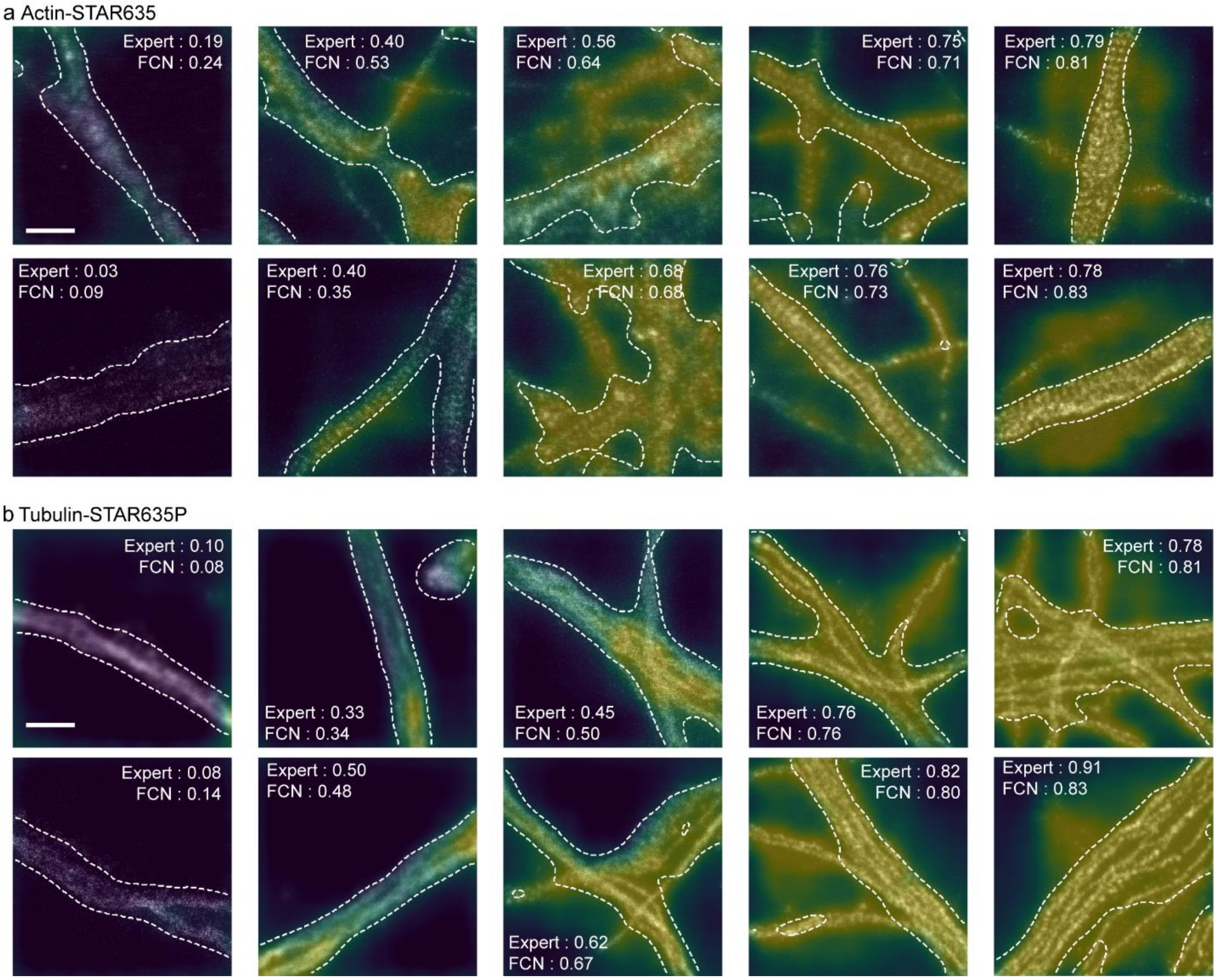

Supplementary Figure 37: Score maps, foreground mask, and scores obtained in fixed cell imaging with different proteins. The FCN model was trained on the Actin dataset (a) and fine-tuned on the Tubulin dataset (b). The colormap is the quality score map. The dotted contour indicates the foreground mask as extracted using Otsu thresholding. Expert and predicted scores are displayed on each image. a) Example images of a) actin stained with phalloidin-STAR635 and b) of tubulin stained with STAR635P. Scale bars 1 µm.

Figure interpretation: We observe that the network focuses on foreground regions to predict the quality score of each image. More specifically, better resolution and higher signal-to-noise ratio, thus better observable structures produce higher quality scores. We observe that the FCN is able to predict scores that are close to the true expert scores.

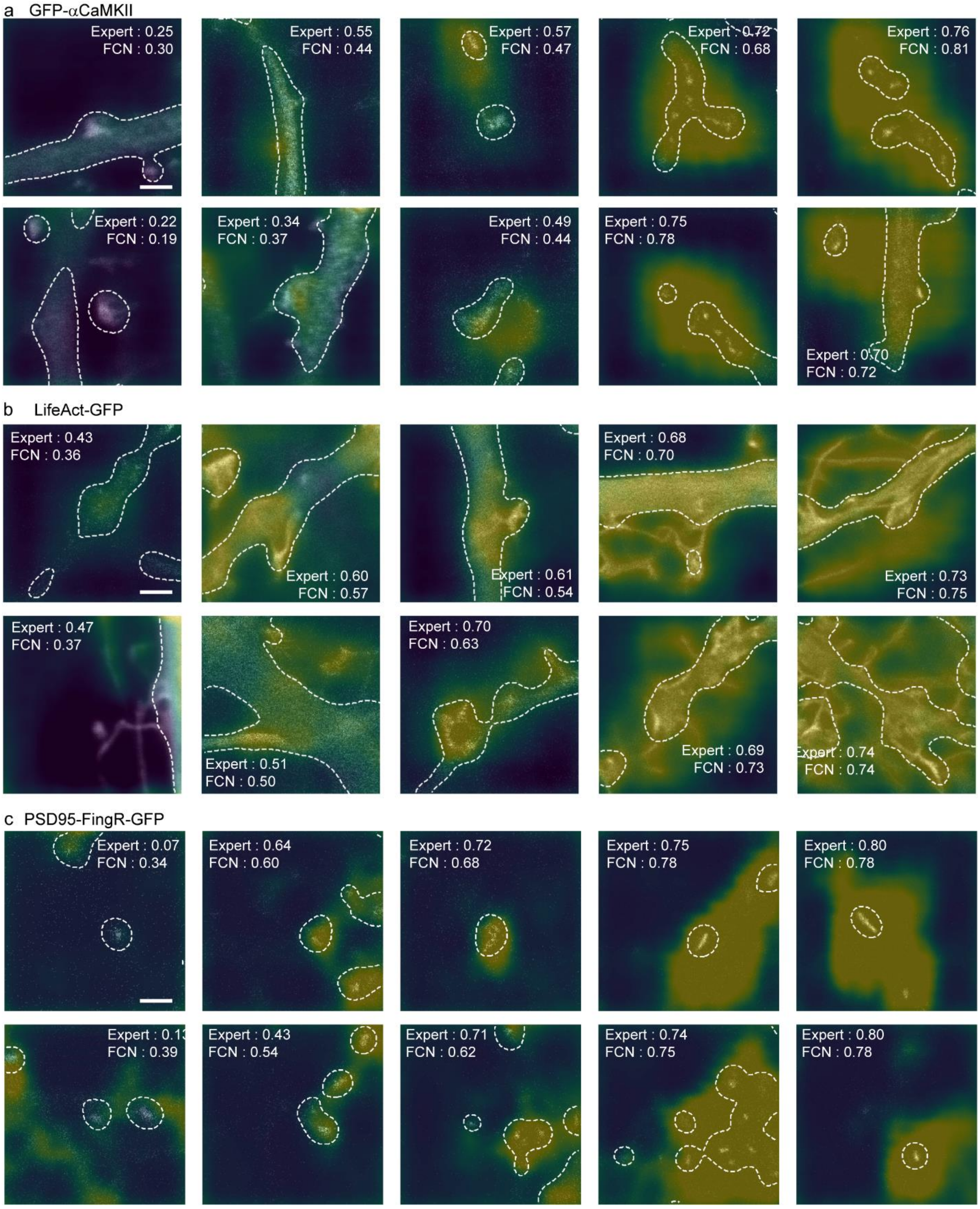

Supplementary Figure 38: Score maps, foreground mask, and scores obtained in live cell imaging with different proteins, using a FCN model trained on the Actin dataset and fine-tuned on each specific protein. The colormap is the quality score map. The dotted contour indicates the foreground, as extracted using Otsu thresholding. Expert and predicted scores are displayed on each image. a) Example images of GFP-α CaMKII, b) LifeAct-GFP, and c) PSD95-FingR-GFP.

Figure interpretation: We observe that the network focuses on foreground regions to predict the quality score of each image. This shows that it is possible to specialize an FCN to different structures, of various sizes. We observe that the fine-tuned FCN models are able to predict quality scores that are close to the true expert scores. Scale bars 1 µm.

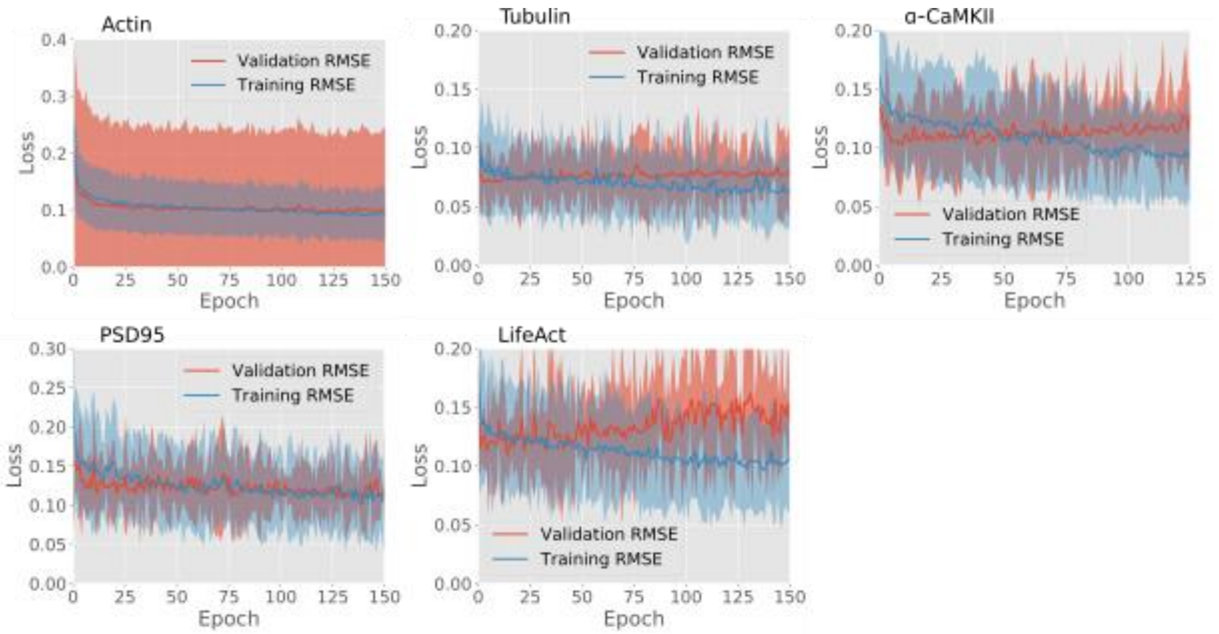

Supplementary Figure 39: Root mean squared error (RMSE) on the training and validation sets during the training of the FCN models on different datasets. Each panel displays the training and validation error on the specific dataset.

Figure interpretation: The FCN model is trained from scratch (e.g. starting with random parameters) using the Actin dataset (top left), which contains around ten times more data than the other datasets (around 2100 compared to a few hundreds). For the other datasets, training from scratch results in severe overfitting, where the FCN model simply learns by heart the small training set without any ability to generalize to new data. Using the model trained on Actin to bootstrap the training on other datasets increases generalization performance in addition of reducing training time. The performance in validation shows that the model generalize well and is able to quickly adapt to a new dataset. Some overfitting emerges on LifeAct and CaMKII after many training epochs. However, this is easy to alleviate by using the validation set to pick the parametrization generalizing the best to unseen data. Note that the inherent variance occurring when an expert labels the data prohibits the network to obtain a perfect match.

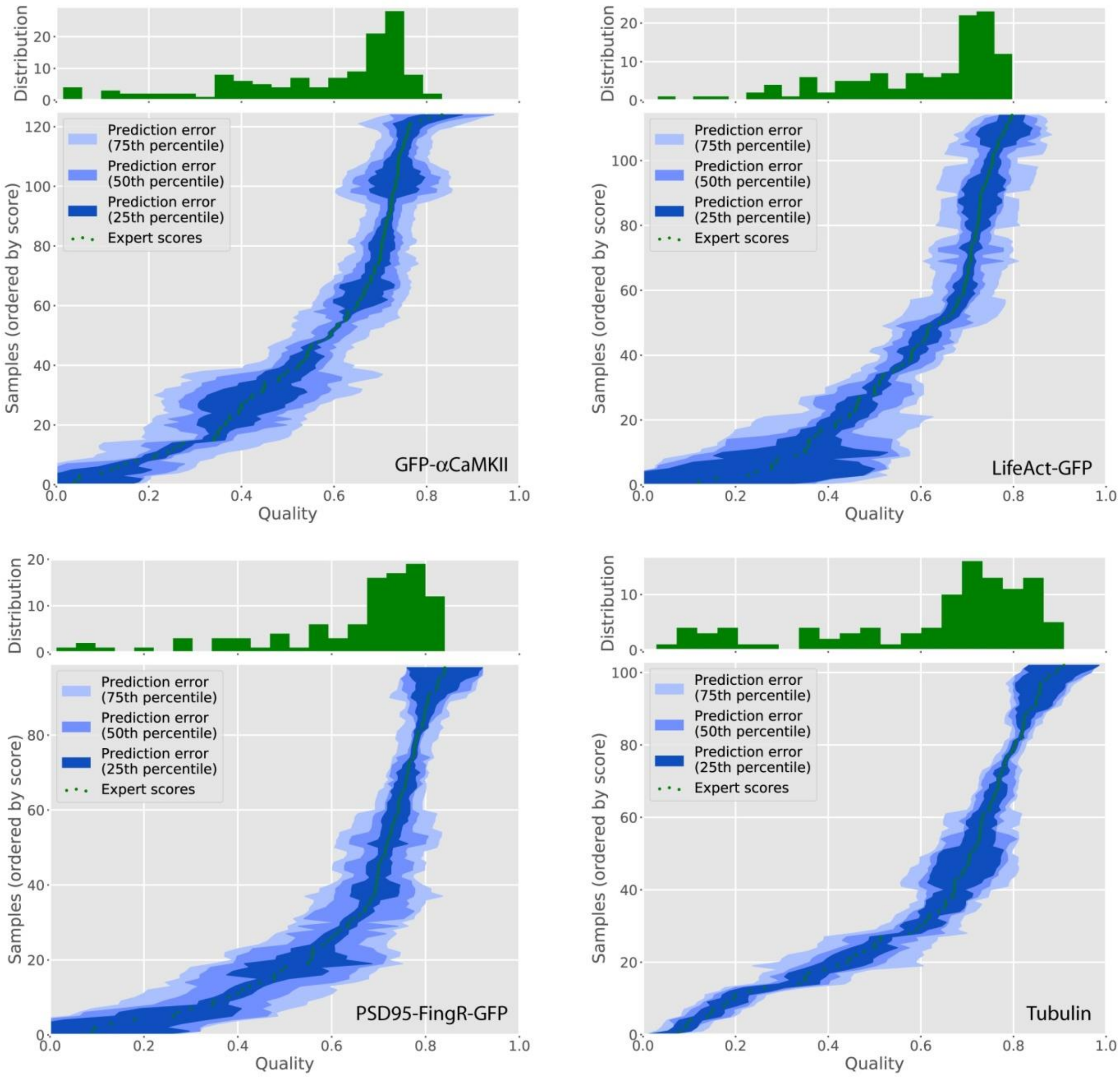

Supplementary Figure 40: Distribution of qualities in the validation sets (top histograms) and error distributions on validation set for the fine-tuned networks. Each panel displays the error distribution of an FCN model pre-trained on the Actin dataset and fine-tuned on the specific dataset. The green dots are the expert scores, sorted by value. The blue areas represent the extent of the errors made by the network at every level of quality.

Figure interpretation: Note how the average error is correlated with the amount of data available at this level of quality. Quite tight around the qualities of interest --- that is, the qualities between 50% and 80% – the error margin becomes higher with very poor quality images. This may be explained by the combination of two factors: dataset imbalance and expert variance. The latter arises from the expert bias, since most experts are accustomed to evaluate relatively good quality images. If an image has poor quality, its exact quality score has little importance since it should be rejected anyway. For this reason, the expert variance is higher in the low scores. The underlying distribution of scores is also skewed towards good quality images, since it was build from actual experiments data, where low quality images have little to no value. The overall median error relative to the expert score is under 10% in most cases.

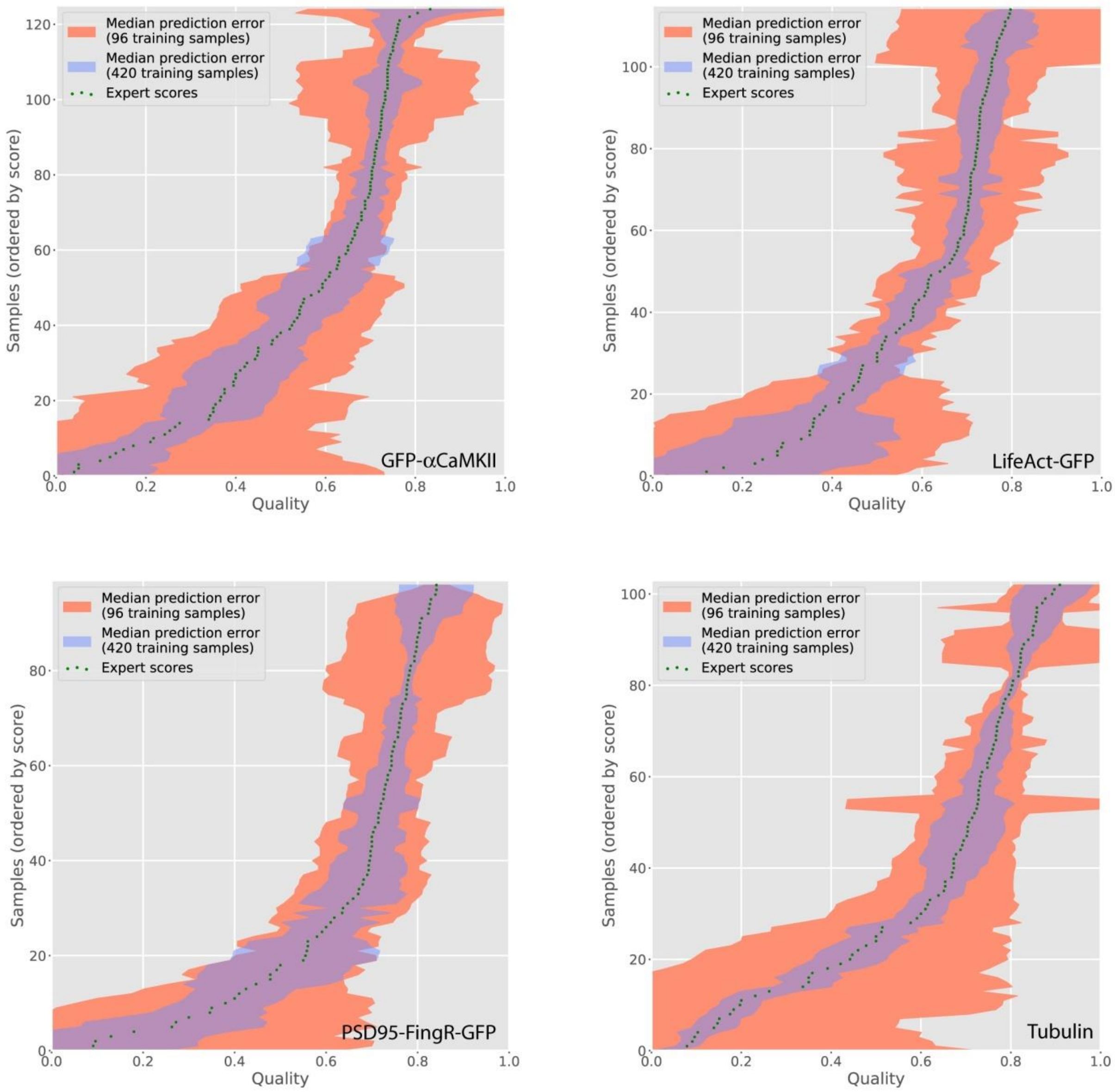

Supplementary Figure 41: Distribution of error distributions on validation set for the networks fine-tuned on 96 images. Each panel displays the error distribution of an FCN model pre-trained on the Actin dataset and fine-tuned on 96 images the specific dataset. The green dots are the expert scores, sorted by value. Orange and blue areas respectively represent the extent of median errors made by the network fine-tuned on 96 images and on the whole dataset, at every level of quality.

Figure interpretation: Note the correlation between the average error and the amount of data available at this level of quality. The network trained with less data exhibits an increased overall error, as expected, but still performs adequately to distinguish good and poor images.

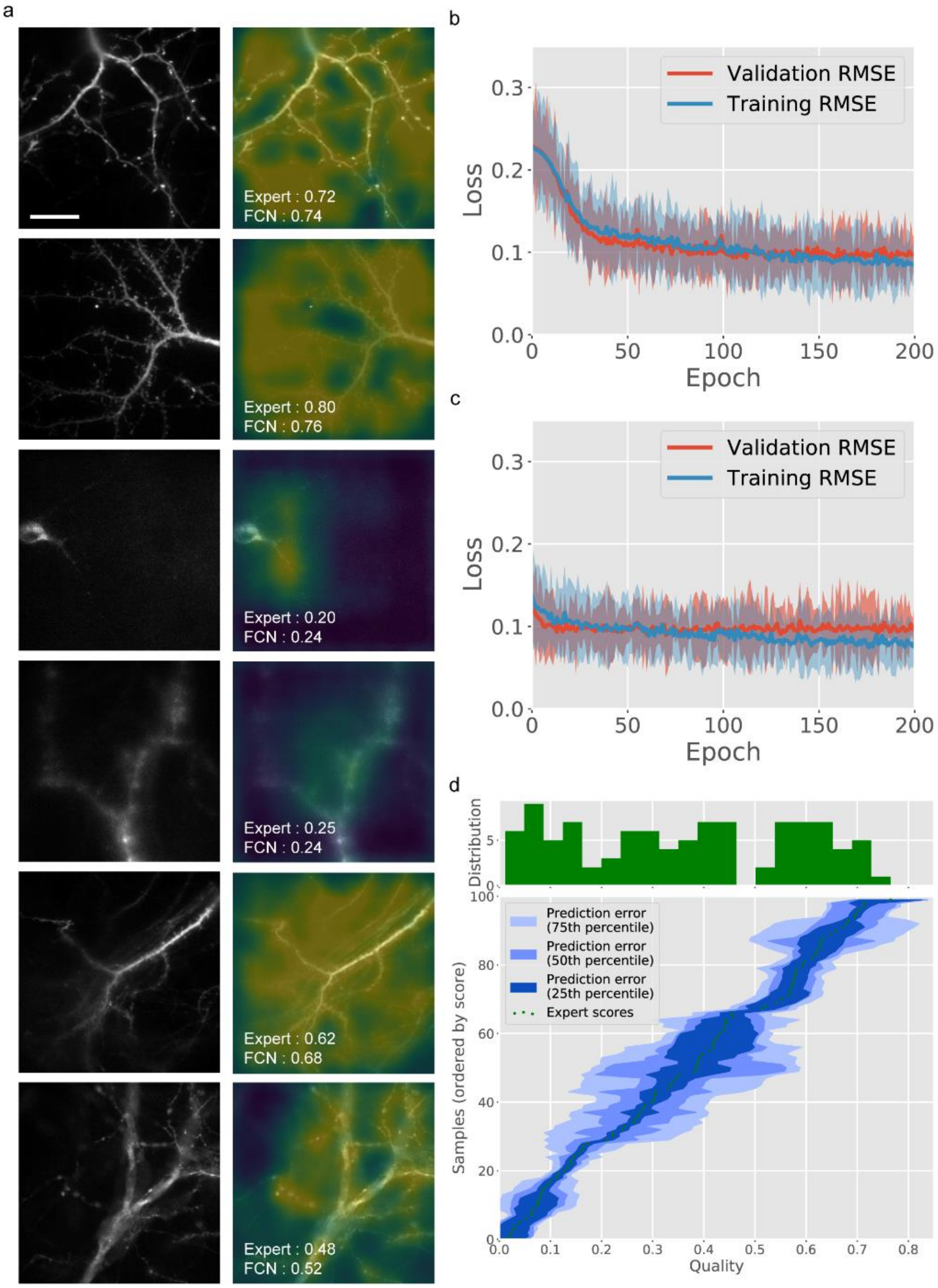
a
Expert : 0.72
FCN : 0.74
Expert : 0.80
FCN : 0.76
Expert : 0.20
FCN : 0.24
Expert : 0.25
FCN : 0.24
Expert : 0.62
FCN : 0.68
Expert : 0.48
FCN : 0.52
b
Validation RMSE
Training RMSE
Loss
Epoch
c
Validation RMSE
Training RMSE
Loss
Epoch
d
Distribution
Prediction error (75th percentile)
Prediction error (50th percentile)
Prediction error (25th percentile)
Expert scores
Samples (ordered by score)
Quality

Supplementary Figure 42: Evaluation of a FCN model trained to rate the quality of images acquired with a widefield microscope. The FCN model is the same than the one tuned for STED images evaluation. a) Images and associated FCN score maps for several test images. b) Root mean squared error (RMSE) on the training and validation sets during the training of a FCN model from scratch (starting with a random parametrization). c) Root mean squared error (RMSE) on the training and validation sets during the training of a FCN model pre-trained on the Actin dataset and fine-tuned on the Widefield dataset. d) Distribution of errors for the network fine-tuned on the Widefield dataset. Scale bar 20 µm.

Figure interpretation: While the final validation performance is roughly the same for both initializations, the convergence is much faster for the pre-trained network. The errors distribution d) offers a better view of the FCN performance. Comparisons with expert decisions (a) also show how the FCN is able to closely match the correct value for all levels of quality. Overall, these results provide strong evidence of the model transferability and shows how the proposed method may be extended to other microscopy-related tasks, even outside the specific scope of STED microscopy.

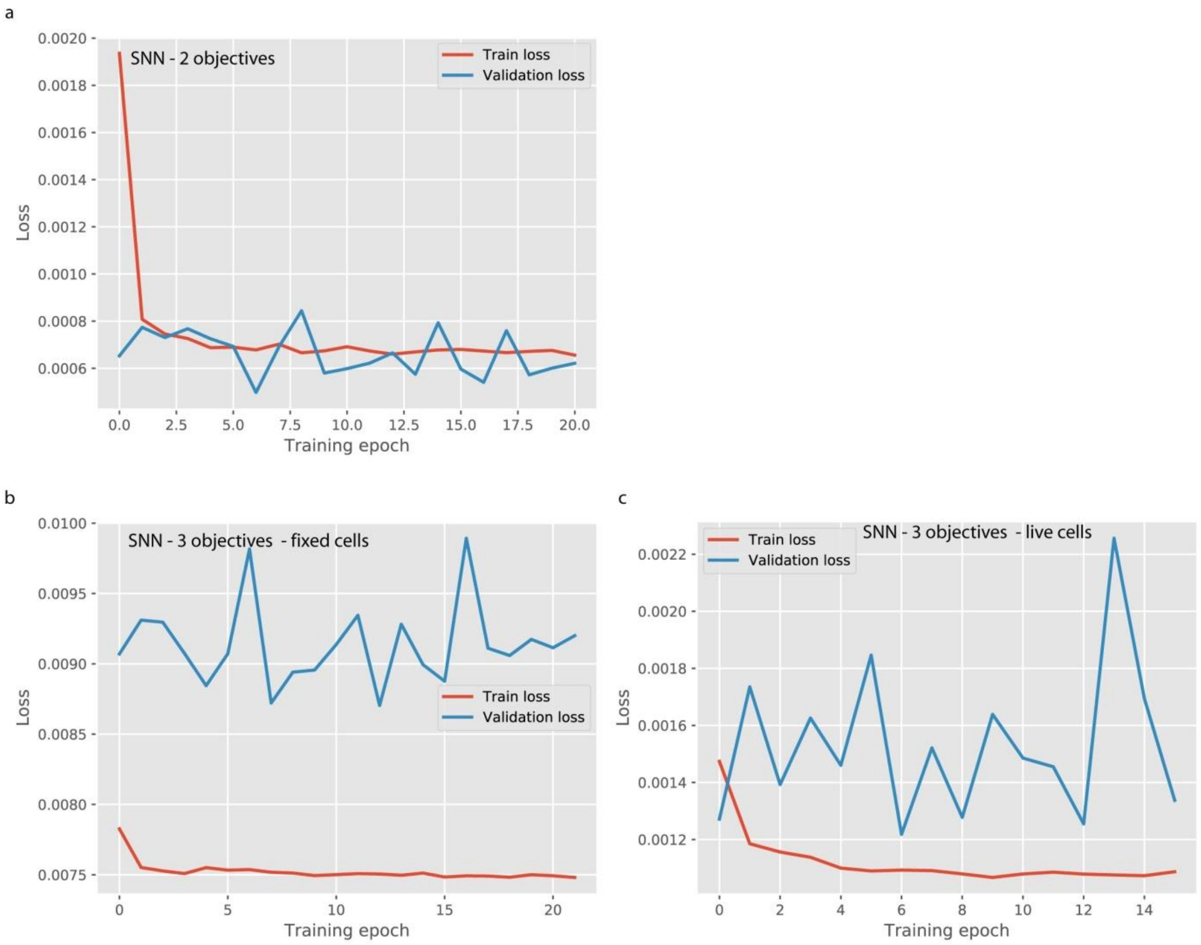

Supplementary Figure 43: Root mean squared error (RMSE) on the training and validation sets during the training of the SNN models. Two models were tried, one for 2-objectives trade-offs (image quality and photobleaching) and one for 3-objectives (image quality, photobleaching and imaging time) trade-offs (both fixed and live cells). a) RMSE of the SNN trained for 2-objectives trade-offs on fixed cells. b) RMSE of the SNN trained for 3-objectives tradeoffs on fixed cells. c) RMSE of the SNN trained for 3-objectives tradeoffs on living cells.

Figure interpretation: Since the SNN exhibits a very small capacity (e.g. number of learnable parameters), the training process requires very little epochs.

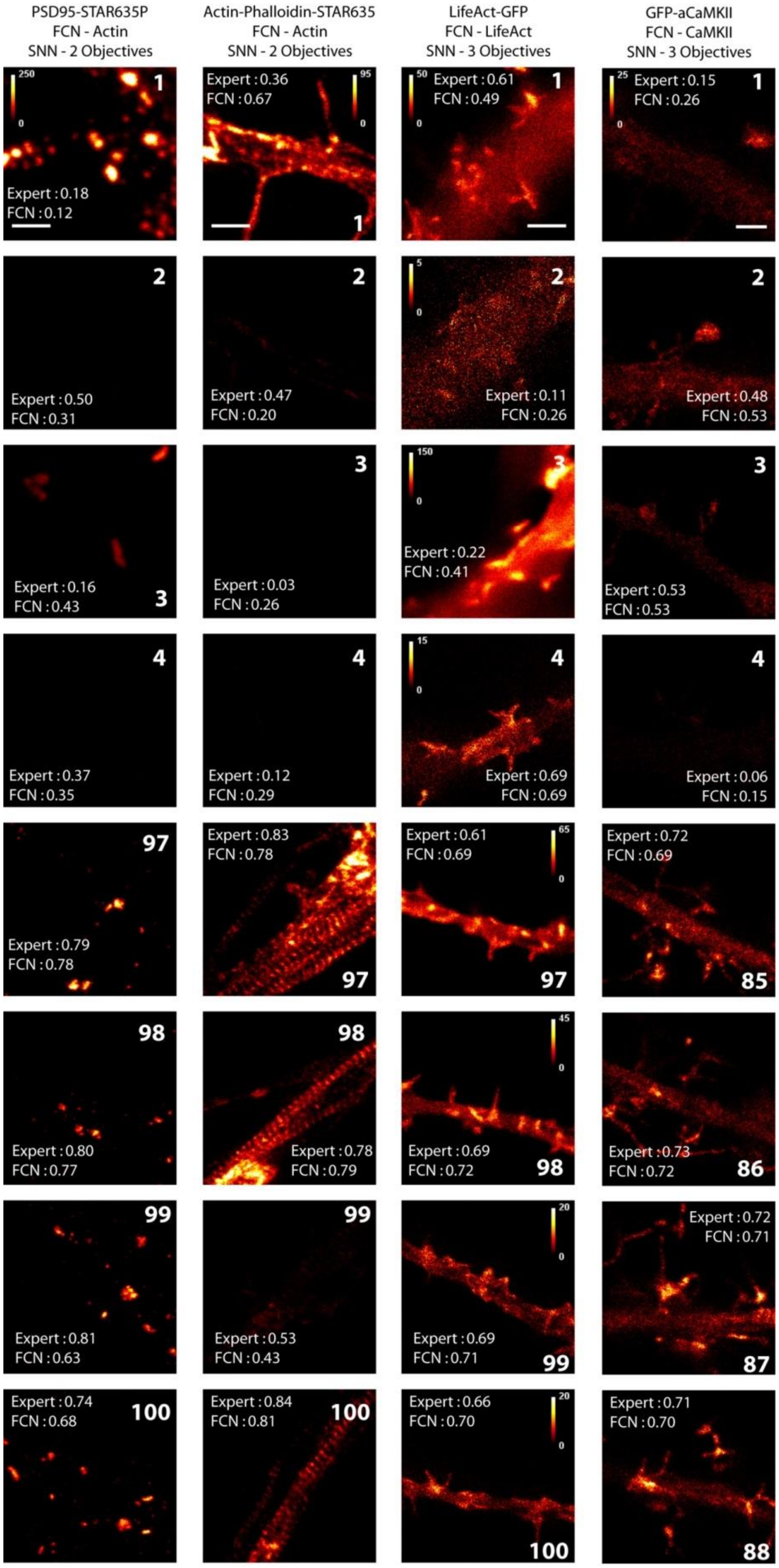

PSD95-STAR635P
FCN - Actin
SNN - 2 Objectives
Actin-Phalloidin-STAR635
FCN - Actin
SNN - 2 Objectives
LifeAct-GFP
FCN - LifeAct
SNN - 3 Objectives
GFP-aCaMKII
FCN - CaMKII
SNN - 3 Objectives
1 Expert : 0.18 FCN : 0.12
1 Expert : 0.36 FCN : 0.67
1 Expert : 0.61 FCN : 0.49
1 Expert : 0.15 FCN : 0.26
2 Expert : 0.50 FCN : 0.31
2 Expert : 0.47 FCN : 0.20
2 Expert : 0.11 FCN : 0.26
2 Expert : 0.48 FCN : 0.53
3 Expert : 0.16 FCN : 0.43
3 Expert : 0.03 FCN : 0.26
3 Expert : 0.22 FCN : 0.41
3 Expert : 0.53 FCN : 0.53
4 Expert : 0.37 FCN : 0.35
4 Expert : 0.12 FCN : 0.29
4 Expert : 0.69 FCN : 0.69
4 Expert : 0.06 FCN : 0.15
97 Expert : 0.79 FCN : 0.78
97 Expert : 0.83 FCN : 0.78
97 Expert : 0.61 FCN : 0.69
85 Expert : 0.72 FCN : 0.69
98 Expert : 0.80 FCN : 0.77
98 Expert : 0.78 FCN : 0.79
98 Expert : 0.69 FCN : 0.72
86 Expert : 0.73 FCN : 0.72
99 Expert : 0.81 FCN : 0.63
99 Expert : 0.53 FCN : 0.43
99 Expert : 0.69 FCN : 0.71
87 Expert : 0.72 FCN : 0.71
100 Expert : 0.74 FCN : 0.68
100 Expert : 0.84 FCN : 0.81
100 Expert : 0.66 FCN : 0.70
88 Expert : 0.71 FCN : 0.70

Supplementary Figure 44: Images obtained at the beginning and at the end of fully-automated optimization on fixed and live neurons. Shown are the four first and last images of 2 objectives (left: PSD95-STAR635P and Actin-Phalloidin-STAR635) and 3 objectives (right: LifeAct-GFP and GFP-α CaMKII) optimization sequences. The quality scores given by an expert user (for validation purpose) after the optimization sequences are compared to the scores given by the networks for all images. Note that one intensity scale was used for the visualization of all images of the same sequence (color bar on the first image) except for LifeAct-GFP (different color bars for each images). Scale bars 1μm.

Figure interpretation: For PSD95-STAR635P, nanometric clusters of the protein with high signal to noise ratio were obtained in the last four images, while either low resolution or low signal to noise ratio was observed in the first four images. For the actin lattice, the periodical pattern could not be detected in none of the four first images, while it was clearly distinguishable in 3 out of the 4 last images. For LifeAct imaging in living neurons, all four last images and only one of the first images were characterized by a quality score above 60 % from both the FCN and the expert. The nanometric distribution of CaMKII in dendritic spines was only visible in the last four images of the optimization sequence, while a lack of resolution and/or signal was observed for the first four images.

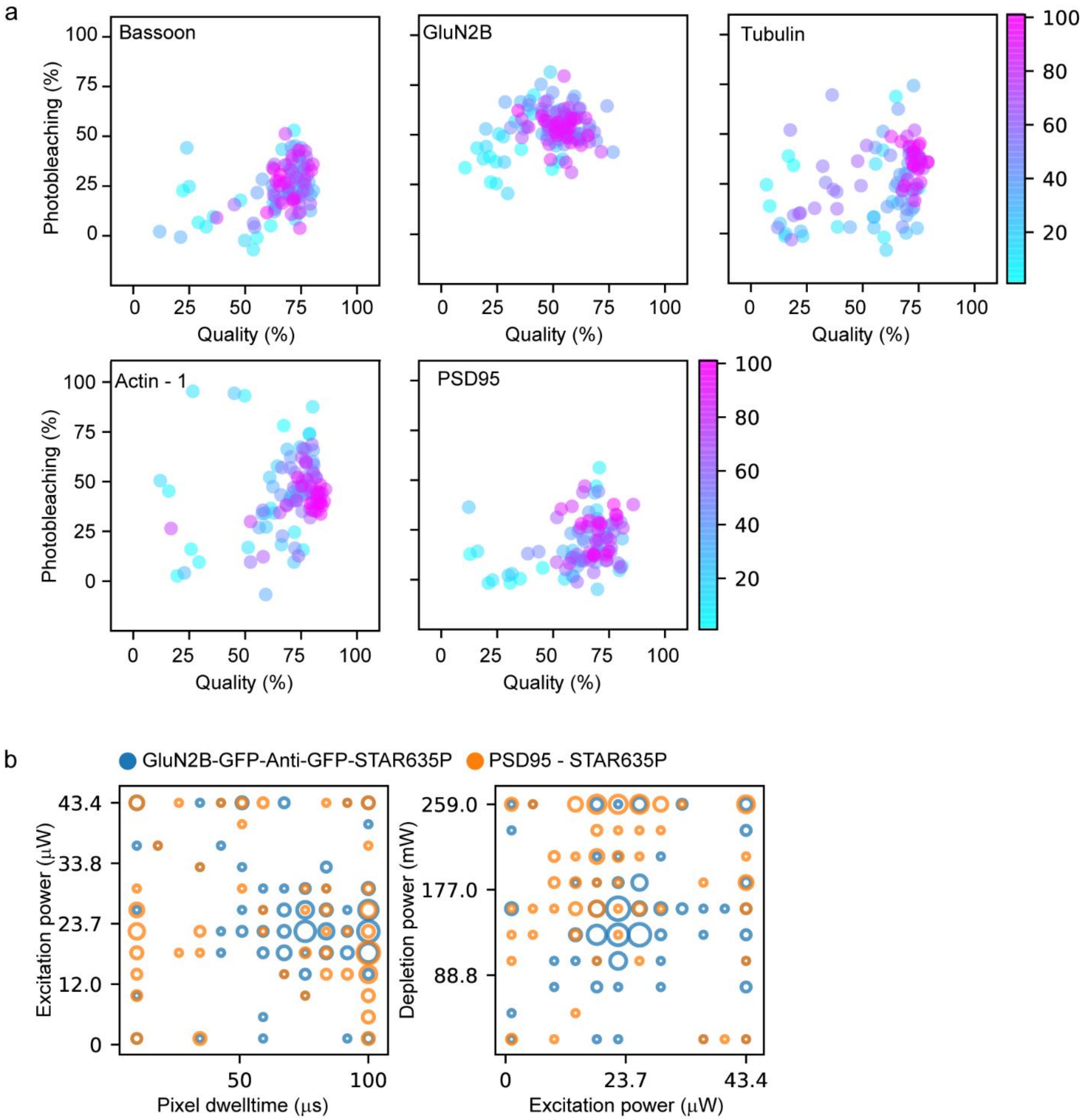

Supplementary Figure 45: Results of fully-automated optimization sequences using the FCN trained on the Actin dataset (see Methods - Datasets) on different proteins. All optimization sequences were performed with the 2 objectives SNN. Three parameters were optimized by Kernel TS: excitation power, depletion power and pixel dwell time. a) Evolution of the quality and photobleaching objectives during optimization on all proteins. b) Parameter configurations selected during optimization using GluN2B and PSD95.

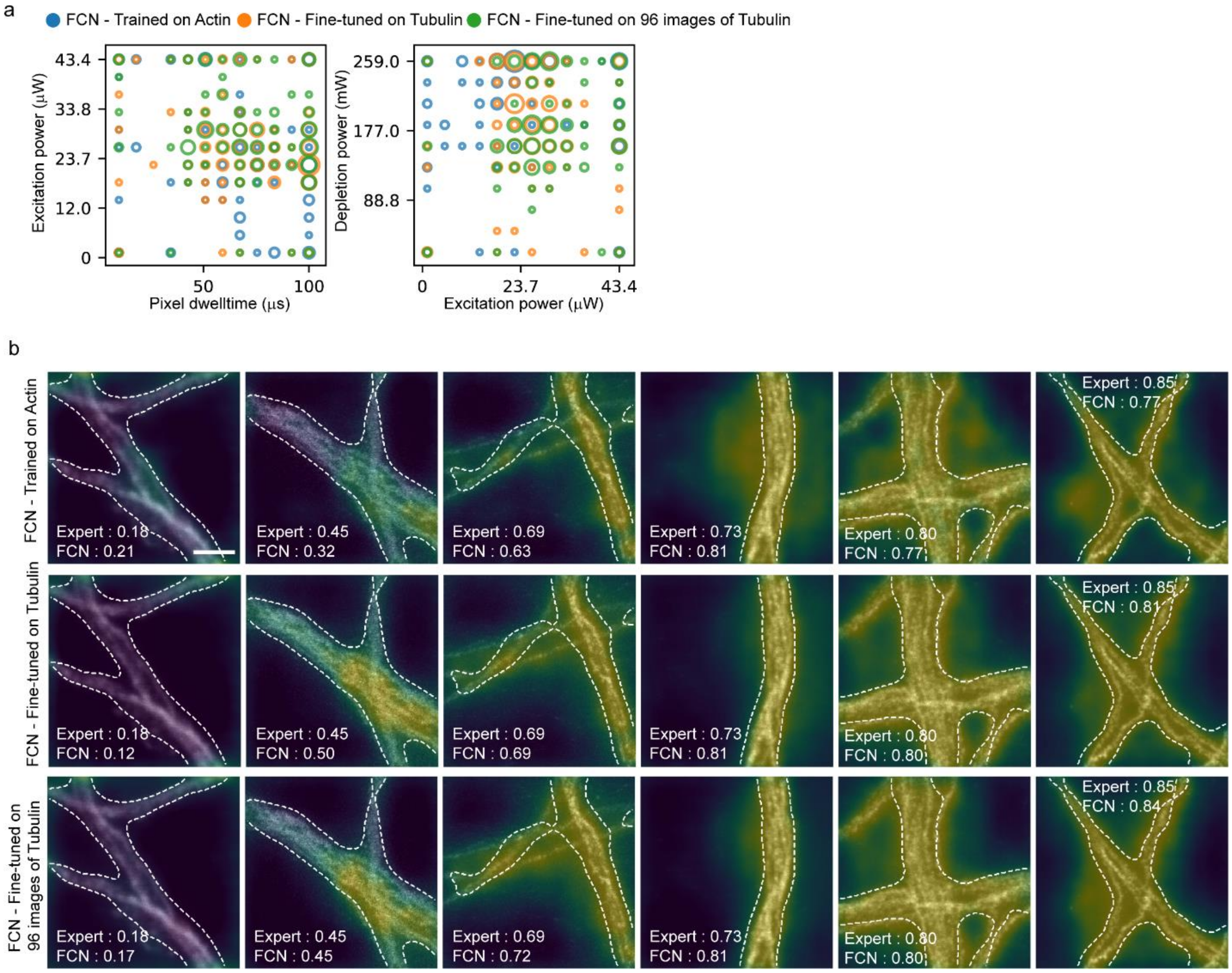

Supplementary Figure 46: Outcome of fully-automated optimization sequences on Tubulin samples using FCN models trained on different datasets: We compare FCN models trained only on the Actin dataset, fine-tuned on the complete Tubulin dataset, and fine-tuned on 96 images of the Tubulin dataset (see Methods). a) Parameter configurations selected during optimization using each FCN model. b) Example of score maps (foreground indicated by dotted contour) and comparison between predicted and expert scores, for each FCN models. Scale bar 1 µm.

Figure interpretation: We observe that all optimization sequences converged to the same region of parameters (a). As expected, it is possible to improve the predicted score of the FCN with respect to the expert by fine-tuning the model (b). Results show that fine-tuning is beneficial, even when performed on a small dataset (hereby 96 images).

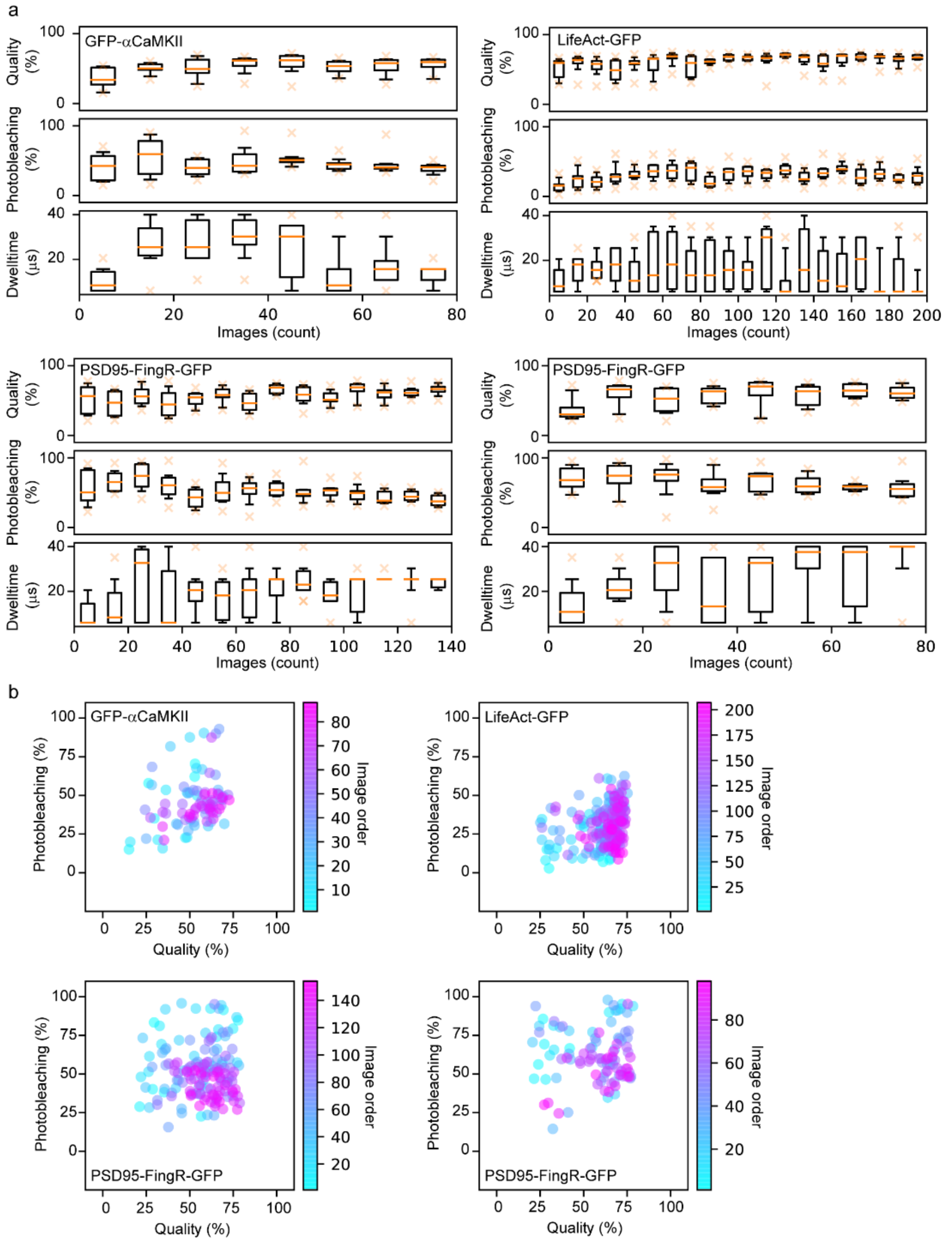

Supplementary Figure 47: Evolution of the objective values during fully-automated optimization sequences of live-cell imaging with different proteins, always using an FCN model fine-tuned on the specific protein. a) Quantitative representation where each box corresponds to the bined objective values of 10 images. b) Alternative representation of the data from a) to visualize the progression of two objectives qualitatively.

Figure interpretation: On a) we observe that the system is able to: 1) improve quality and reduce pixel dwelltime while controlling photobleaching on GFP-CaMKII; 2) reduce variance in quality while controlling photobleaching and pixel dwelltime (LifeAct); 3) improve quality and reduce photobleaching while controlling pixel dwelltime (PSD95 left); and 4) improve quality and reduce photobleaching, at the price of increasing pixel dwelltime (PSD95 right). This shows that the system is able to converge to different objectives trade-offs relevant to the task at hand. This convergence is supported by b) where we can see that the quality and photobleaching objectives converge to a tradeoff as we progress into the optimization sequence.

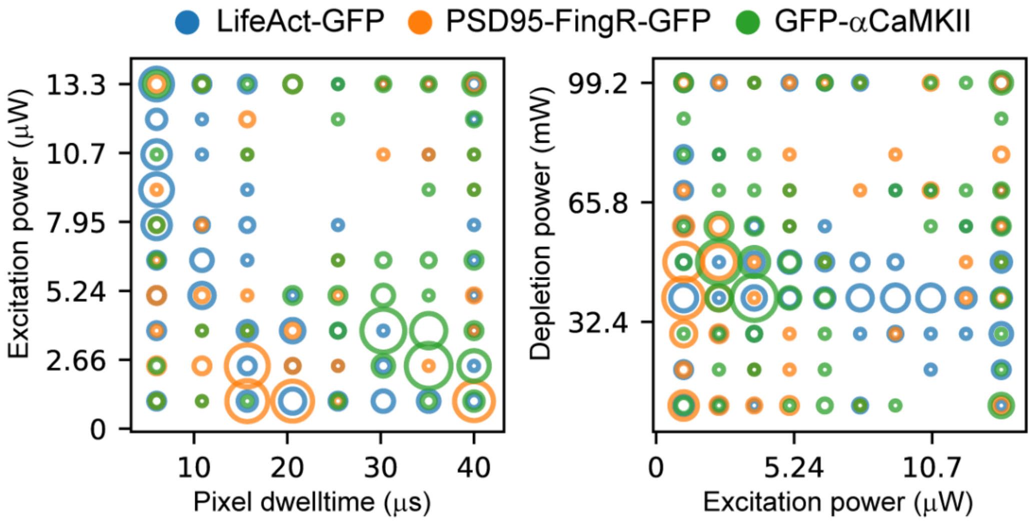

Supplementary Figure 48: Parameter configurations selected during fully-automated optimization sequences of live-cell imaging with different proteins, always using an FCN model fine-tuned on the specific protein.

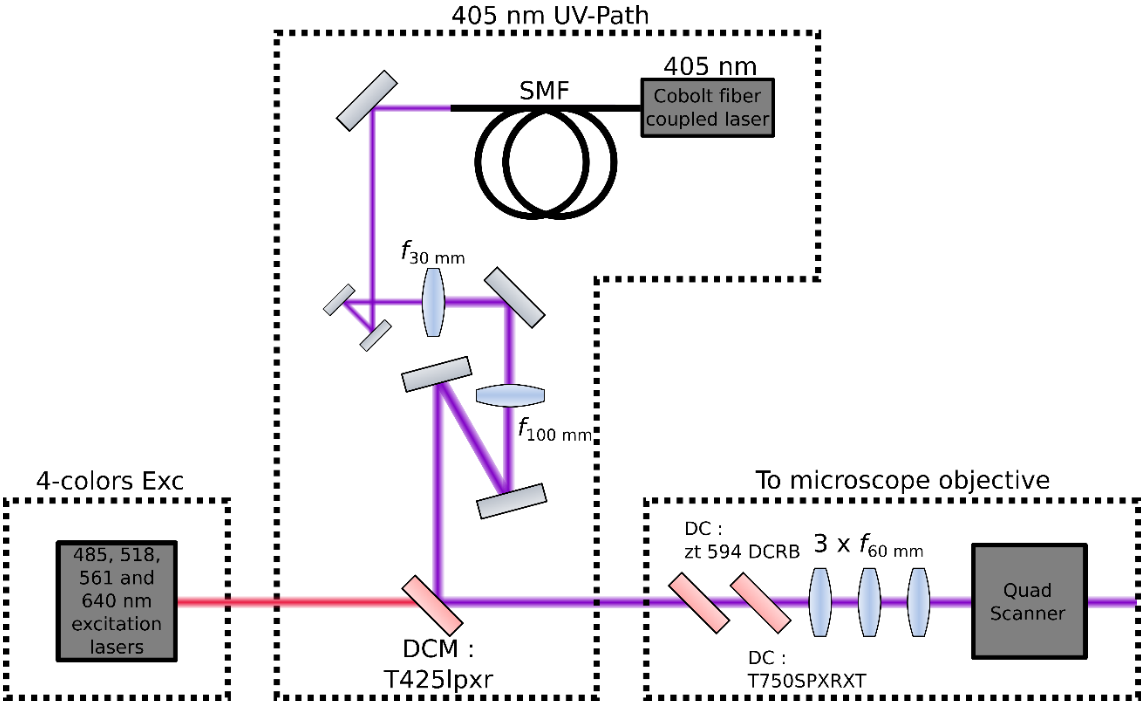

Supplementary Figure 49: Schematic drawing of the custom UV-path integrated to the Abberior STED Microscope. The 405 nm diode laser (Cobolt, 06-01 Series 405 nm) is coupled to an optical fiber (Single Mode Fiber, NA: 0.1, core diameter: 3.1) to route the laser light inside the Abberior microscope box. The 405 nm light is collimated at the end of the optical fiber with an adjustable aspheric collimator (Thorlabs, CFC-8X-A, focal length: 7.5 mm) and undergoes a magnification as it passes through the beam expander. The laser beam is redirected towards the microscope using a dichroic mirror (Chroma, T425lpxr and passes through two laser beam splitters (AHF Analysentechnik, zt 594 DCRB and T750SPXRXT) both responsible for the reflection of the STED beams to the microscope objective. All laser beams are relayed to a four-galvanometer mirror beam scanner (Abberior QUAD scanner). Abbreviations: dichroic mirror (DCM), focal length (*f*), single mode fiber (SMF), dichroic beam splitter (DC), numerical aperture (NA).
